# Pregnancy and parenthood in surgical training: a cross-sectional survey in the UK

**DOI:** 10.1093/bjs/znad204

**Published:** 2023-07-18

**Authors:** Jessica Whitburn, Saiful Miah, Sarah A Howles

**Affiliations:** Nuffield Department of Surgical Sciences, University of Oxford, Oxford, UK; Department of Urology, Addenbrookes Hospital, Cambridge University Hospitals, Cambridge, UK; Nuffield Department of Surgical Sciences, University of Oxford, Oxford, UK

Women were first admitted to medical schools in the late 19th century and, since then, female representation in the medical profession has gradually increased^1^. Female representation within surgical specialties, remains poor, with females making up only 13 per cent of consultant surgeons in England and 27 per cent of the surgical workforce as a whole^2^.

There has been a drive to understand the causes of this gap between the sexes. Studies have reported that surgery may be seen as an unattractive career for female undergraduates due to perceived difficulties in maintaining family life, limited opportunities for flexible training, and a lack of role models^3–6^. Furthermore, data from the USA and Ireland indicate that female surgeons have fewer children, are more likely to delay parenthood, are at greater risk of pregnancy-associated complications, and have higher rates of infertility^7–10^.

To increase understanding of experiences of parenthood and pregnancy-associated risks in surgical training in the UK, an anonymized, online, cross-sectional survey of 416 surgical trainees/residents (346 females and 70 males; approximately 5 per cent response rate) was undertaken between 24 June and 24 December 2022 (*Fig. S1*, Appendix S1, Supplementary Methods). This research was conducted under ethical approval granted via the Central University Research Ethics Committee, University of Oxford (Ref: R80778/RE001).

Surgeons spanned all training grades (5 per cent CT1-2, 45 per cent ST3-5, 35 per cent ST6-8, and 16 per cent out of programme/other). A total of 352 live births during surgical training were reported by 171 childbearing and 41 non-childbearing individuals (*Table 1*). Childbearing and non-childbearing trainee surgeons had similar numbers of children (mean(s.d.) = 0.84(0.99) *versus* 1.04(0.10), median = 1 (95 per cent c.i. 0 to 1) *versus* 1 (95 per cent c.i. 1 to 1); *Table 1*). A total of 56 per cent of childbearing and 40 per cent of non-childbearing surgical trainees delayed parenthood due to their training, and both male and female surgeons underwent infertility investigations more frequently than would be expected in the general UK population (*Table 1*)^11^. The majority (greater than 80 per cent) of trainee surgeons regretted their decision to delay parenthood. The risks of increased infertility and obstetric complications with increasing maternal age are well recognized. Cultural change and open discussion may enable surgical trainees to make better informed decisions about timing of attempts to become a parent.

**Table 1:** Characteristics of the survey participants

	Childbearing surgeon, *n* = 346	Non-childbearing surgeon, *n* = 70	*P*
**Relationship status**			
Single, never married	31 (9)	0 (0)	0.015
Married/civil partnership/significant other	308 (89)	70 (100)	
Divorced/separated and single	7 (2)	0 (0)	
**Race or ethnicity**			
White/Caucasian	273 (79)	46 (66)	0.022
Asian/Asian British	38 (11)	17 (24)	
Mixed/multiple ethnic groups	18 (5)	3 (4)	
Black, African, Caribbean/black British	13 (4)	1 (1)	
Other	4 (1)	2 (3)	
Not stated	0 (0)	1 (1)	
**Age (years)**			
25–29	37 (11)	6 (9)	0.672
30–34	171 (49)	38 (54)	
35–39	122 (35)	22 (31)	
40–44	15 (4)	3 (4)	
>45	1 (1)	1 (1)	
**Surgical specialty**			
Cardiothoracic Surgery	2 (1)	0 (0)	<0.001
Ear, Nose, and Throat Surgery	31 (9)	12 (17)	
General Surgery	42 (12)	3 (4)	
Neurosurgery	6 (2)	1 (1)	
Obstetrics and Gynaecology	107 (31)	1 (1)	
Oral and Maxillofacial Surgery	9 (3)	2 (3)	
Paediatric Surgery	8 (2)	0 (0)	
Plastic Surgery	12 (3)	3 (4)	
Trauma and Orthopaedics	27 (8)	3 (4)	
Urology	75 (22)	42 (60)	
Vascular Surgery	16 (5)	3 (4)	
Other	11 (3)	0 (0)	
**Partner occupation**			
Physician	80 (26)	39 (56)	<0.001
Surgeon	40 (13)	2 (3)	
Other	183 (59)	28 (40)	
Unemployed	4 (1)	1 (1)	
Prefer not to answer	1 (0)	0 (0)	
**Partners working hours/week (excluding domestic obligations)**			
0–19 h	7 (2)	8 (11)	0.001
20–39 h	79 (26)	24 (34)	
40–59 h	184 (60)	37 (52)	
>60 h	38 (11)	1 (1)	
**Number of biological children**			
0	172 (50)	26 (37)	0.283
1	81 (23)	21 (30)	
2	70 (20)	18 (26)	
≥3	23 (7)	5 (7)	
Number of children, mean	0.84	1.04	0.125
Age of child bearer at first live birth (years), mean	31.9	31.2	0.097
Undergone infertility testing	53 (24)	11 (22)	0.855

Values are *n* (%) unless otherwise stated.

Of the 222 childbearing surgical trainees who had been pregnant, 36 per cent had experienced a pregnancy loss, with a pregnancy loss rate of 31 per cent. These findings are comparable to data from non-surgeon partners, which may reflect the fact that nearly 60 per cent of childbearing partners of surgical trainees in this survey were doctors (*Table 2*). In all age groups, rates of pregnancy loss in surgeons were higher than those reported in a large registry-based study (*Table 2*)^13^. Of note, loss rates were 35 per cent in those aged under 35 years at the time of survey completion, a rate that is over three times that which would be expected in this age group (*Table 2*). After pregnancy loss, one in three childbearing surgical trainees did not take any time off work, and only 47 per cent disclosed their loss to a colleague. In general, surgical trainees who did share their loss with colleagues found them to be supportive (*Table S1*).

**Table 2:** Pregnancy loss and complications in surgical training

	Childbearing surgeon	Non-surgeon partner	*P* value or expected pregnancy loss rate*
Individuals who experienced pregnancy loss	80 (36)	19 (40)	0.619
**Gestation at pregnancy loss**			
Losses at <12 weeks	114 (90)	24 (83)	0.287
Losses at 12–24 weeks	13 (10)	5 (17)	
Still births	0 (0)	0 (0)	
**Pregnancy loss rate, %**			
All ages (410^†^)	31	30 (19)	‡
<30 years^§^ (4^†^)	25	‡	10
30–34 years^§^ (115^†^)	35	‡	11
35–39 years^§^ (246^†^)	30	‡	17
≥40 years^§^ (45^†^)	36	‡	32
**Pregnancy complications**			
Pregnancy with complication requiring bedrest	50 (18)	10 (14)	0.596
Pregnancy with one or more minor complications	175 (62)	35 (51)	0.100
Pregnancy with one or more major complications	87 (31)	6 (9)	0.001
Delivery with one or more intrapartum complications	60 (21)	9 (13)	0.132
Delivery with one or more postpartum complications	27 (10)	4 (6)	0.477
**Neonatal complications**			
NICU stay	17 (6)	3 (4)	0.741
Delivery <38 weeks	35 (12)	7 (10)	

Values are *n* (%) unless otherwise stated. *Pregnancy loss rates as reported in ages 25–29, 30–34, 35–39, and 40–44 years in Magnus *et al*.^12^ †Participant age at time of survey completion. ‡Data not available. §Total number of pregnancies in childbearing surgeons in this age group. NICU, neonatal intensive care unit.

The psychological impact of early pregnancy loss is well recognized^14–16^. Surgical training programmes should consider offering bereavement support and mandated leave after pregnancy loss to reduce the burden of guilt associated with taking time off work. Appointment of a lead clinician for pregnancy support may help trainees navigate this difficult area, providing a single point of contact to facilitate changes to working schedules in early pregnancy and acting to offer guidance after loss.

Childbearing surgical trainees experienced minor and major pregnancy-associated complications in 62 and 31 per cent of pregnancies, respectively (*Table 2*). Major pregnancy complications were more likely in childbearing surgical trainees than the partners of non-childbearing surgical trainees (*Table 2*). Approximately 60 per cent of trainees who required time away from work due to pregnancy-associated complications felt their colleagues and training programme were supportive. Twelve childbearing surgical trainees reported a financial loss, ranging from £3000 to £50 000 (median £7500), due to work restriction during pregnancy. Rates of neonatal, intrapartum, and postpartum complications were higher in childbearing surgical trainees than in the partners of surgical trainees; however, statistical significance was not reached, this may be due to the small sample size (*Table 2*). Children of childbearing trainees were of similar weight to those of non-childbearing trainees (*Table S2*).

Over 70 per cent of childbearing surgical trainees worked for 40 h or more each week during pregnancy, and 4 per cent worked more than 60 h per week. Most trainees continued to work at night throughout their pregnancy, and half continued to operate for more than 9 h each week up until maternity leave (*Table S3*). The majority (70 per cent) of childbearing surgical trainees altered their work schedule during pregnancy; 77 per cent felt guilty for burdening their colleagues by reducing their workload. Of those trainees who did not alter their work schedule in pregnancy, 40 per cent made this decision to avoid being ‘considered weak’ and 35 per cent because of concerns surrounding burdening colleagues.

Night shifts and working more than 40 h a week are associated with higher risks of miscarriage and adverse pregnancy outcomes; additionally, adverse pregnancy outcomes are associated with prolonged standing and high fatigue scores^12,17–20^. This study suggests that, even in combination with statutory maternity leave policies, the rest and working time requirements of the European Working Time Directive (EWTD) afford insufficient protection to pregnant surgical trainees. The outcomes from this study mirror findings reported in North America^7,21–23^. Employers should, as a matter of priority, offer altered working schedules to pregnant surgical trainees to protect obstetric health. Furthermore, these data support childbearing surgeons being considered a high-risk obstetric group^8,24^.

The majority (98 per cent) of childbearing surgical trainees wanted to breastfeed their child, and 85 per cent achieved this for over 6 months. The most common reason for surgical trainees stopping breastfeeding early was inadequate work provision; steps should be taken to enable mothers to continue breastfeeding for as long as desired. Other barriers to breastfeeding included insufficient milk supply, conflict with work schedules, and fatigue or illness.

Childbearing surgical trainees took a mean of 10.2 months of parental leave; 73 per cent of non-childbearing surgical trainees took 2 weeks of parental leave. Two thirds of non-childbearing surgical trainees felt that the amount of leave taken was insufficient (27), but cited concerns that longer times would be viewed negatively by colleagues and a reluctance to prolong training. Only a third of non-childbearing surgical trainees found they were able to organize their parental leave without any difficulties (*Table S4*).

On return to work after parental leave, 61 per cent (104) of childbearing surgical trainees reduced their working hours to less than 40 h per week, compared with 15 per cent (6) of non-childbearing surgical trainees. Approximately 10 per cent of both childbearing and non-childbearing surgical trainees altered their work schedules after parental leave to accommodate family life, but continued to work more than 40 h per week. Nearly one in four non-childbearing trainees were unhappy with their work schedule on return to work; the majority did not change their work schedule due to concerns related to the views of their colleagues (*Table S5*). It may be that adopting a Scandinavian model of shared paid parental leave, with intervals ring-fenced for each parent, would lead to cultural change with respect to childcare in the medical workforce.

It is likely that selection bias was present in this study; indeed, most survey responses were from female trainees. In addition, this study could have collated views of surgical trainees and trainers on the impact of providing support and cover to pregnant colleagues. Furthermore, these findings are limited to the experiences of surgical trainees in the UK, so caution should be exercised before initiating changes outside of this setting.

This study highlights the need for urgent action to reduce rates of miscarriage and major pregnancy complications in surgical trainees in EWTD-compliant settings. Altering working schedules and limiting physical exertion may mitigate these risks; however, consideration will need to be taken of the impact of such changes on the surgical workforce as a whole and on the training opportunities and income of pregnant surgeons. Furthermore, cultural change is required to empower pregnant surgical trainees to access modifications to their working schedule and practice, as well as to enable non-childbearing surgical parents to take longer intervals of parental leave and alter work schedules after becoming a parent. Training programmes and national surgical associations must evolve to progress diversity in the surgical workforce and protect the obstetric health of surgeons.

## Supplementary Material

znad204_Supplementary_Data

## Data Availability

Data utilized in the preparation of this manuscript will be made available to researchers upon reasonable request to the corresponding author.

## References

[1] Jefferson L , Bloor K, Maynard A. Women in medicine: historical perspectives and recent trends. Br Med Bull 2015;114:5–15 25755293 10.1093/bmb/ldv007

[2] *Medical and Dental Staff by Gender, Specialty and Grade AH2667* from *28 February 2010 to 28 February 2019*. https://digital.nhs.uk/data-and-information/find-data-and-publications/supplementary-information/2019-supplementary-information-files/medical-and-dental-staff-by-gender-specialty-and-grade-ah2667. Published 6 June 2019.

[3] Fysh THS , Thomas G, Ellis H. Who wants to be a surgeon? A study of 300 first year medical students. BMC Med Educ 2007;7:2 17239248 10.1186/1472-6920-7-2 PMC1784089

[4] Fitzgerald JEF , Tang SW, Ravindra P, Maxwell-Armstrong CA. Gender-related perceptions of careers in surgery among new medical graduates: results of a cross-sectional study. Am J Surg 2013;206:112–119 22902099 10.1016/j.amjsurg.2012.04.009

[5] Kerr HL , Armstrong LA, Cade JE. Barriers to becoming a female surgeon and the influence of female surgical role models. Postgrad Med J 2016;92:576–580 27528701 10.1136/postgradmedj-2015-133273

[6] Bellini MI , Graham Y, Hayes C, Zakeri R, Parks R, Papalois V. A woman’s place is in theatre: women’s perceptions and experiences of working in surgery from the Association of Surgeons of Great Britain and Ireland women in surgery working group. BMJ Open 2019;9:e024349 10.1136/bmjopen-2018-024349 PMC6326292 30617103

[7] Lerner LB , Stolzmann KL, Gulla VD. Birth trends and pregnancy complications among women urologists. J Am Coll Surg 2009;208:293–297 19228542 10.1016/j.jamcollsurg.2008.10.012

[8] Hamilton AR , Tyson MD, Braga JA, Lerner LB. Childbearing and pregnancy characteristics of female orthopaedic surgeons. J Bone Joint Surg 2012;94:e77 22637217 10.2106/JBJS.K.00707

[9] Eskenazi L , Weston J. The pregnant plastic surgical resident: results of a survey of women plastic surgeons and plastic surgery residency directors. Plast Reconstr Surg 1995;95:330–335 7824613

[10] Rogers AC , Wren SM, McNamara DA. Gender and specialty influences on personal and professional life among trainees. Ann Surg 2019;269:383–387 29099401 10.1097/SLA.0000000000002580

[11] Wilkes S , Chinn DJ, Murdoch A, Rubin G. Epidemiology and management of infertility: a population-based study in UK primary care. Fam Pract 2009;26:269–274 19502575 10.1093/fampra/cmp029

[12] Magnus MC , Wilcox AJ, Morken NH, Weinberg CR, Håberg SE. Role of maternal age and pregnancy history in risk of miscarriage: prospective register based study. BMJ 2019;364:l869 30894356 10.1136/bmj.l869 PMC6425455

[13] Snijder CA , Brand T, Jaddoe V, Hofman A, Mackenbach JP, Steegers EAP et al Physically demanding work, fetal growth and the risk of adverse birth outcomes. The Generation R Study. Occup Environ Med 2012;69:543–550 22744766 10.1136/oemed-2011-100615

[14] Farren J , Jalmbrant M, Ameye L, Joash K, Mitchell-Jones N, Tapp S et al Post-traumatic stress anxiety and depression following miscarriage or ectopic pregnancy: a prospective cohort study. BMJ Open 2016;6:e011864 10.1136/bmjopen-2016-011864 PMC5129128 27807081

[15] Farren J , Mitchell-Jones N, Verbakel JY, Timmerman D, Jalmbrant M, Bourne T. The psychological impact of early pregnancy loss. Hum Reprod Update 2018;24:731–749 30204882 10.1093/humupd/dmy025

[16] Kulathilaka S , Hanwella R, de Silva VA. Depressive disorder and grief following spontaneous abortion. BMC Psychiatry 2016;16:100 27071969 10.1186/s12888-016-0812-y PMC4830021

[17] Cai C , Vandermeer B, Khurana R, Nerenberg K, Featherstone R, Sebastianski M et al The impact of occupational shift work and working hours during pregnancy on health outcomes: a systematic review and meta-analysis. Am J Obstet Gynecol 2019;221:563–576 31276631 10.1016/j.ajog.2019.06.051

[18] Jin LZ , Hjollund NH, Andersen AMN, Olsen J. Shift work, job stress, and late fetal loss: the National Birth Cohort in Denmark. J Occup Environ Med 2004;46:1144–1149 15534501 10.1097/01.jom.0000145168.21614.21

[19] Begtrup LM , Specht IO, Hammer PEC, Flachs EM, Garde AH, Hansen J et al Night work and miscarriage: a Danish nationwide register-based cohort study. Occup Environ Med 2019;76:302–308 30910992 10.1136/oemed-2018-105592

[20] Behbehani S , Tulandi T. Obstetrical complications in pregnant medical and surgical residents. J Obstet Gynaecol Can 2015;37:25–31 25764033 10.1016/S1701-2163(15)30359-5

[21] Rangel EL , Castillo-Angeles M, Easter SR, Atkinson RB, Gosain A, Hu YY et al Incidence of infertility and pregnancy complications in US female surgeons. JAMA Surg 2021;156:905–915 34319353 10.1001/jamasurg.2021.3301 PMC9382914

[22] Phillips EA , Nimeh T, Braga J, Lerner LB. Does a surgical career affect a woman’s childbearing and fertility? A report on pregnancy and fertility trends among female surgeons. J Am Coll Surg 2014;219:944–950 25260684 10.1016/j.jamcollsurg.2014.07.936

[23] Malcolm RJ , Pilkington M, Merchant SJ, Velez MP, Brogly SB. Work-related factors and pregnancy outcomes in female surgeons. Ann Surg 2021;2:e069 10.1097/AS9.0000000000000069 PMC10455228 37636550

[24] Miller NH , Katz VL, Cefalo RC. Pregnancies among physicians. A historical cohort study. J Reprod Med 1989;34:790–796 2795562

