# Supplementary material for: Pregnancy and parenthood in surgical training: a cross-sectional survey in the UK
Source: Br J Surg. 2023 Jul 18;110(12):1628–31. doi: 10.1093/bjs/znad204 (PMC10638525; doi:10.1093/bjs/znad204)
Supplement: znad204_Supplementary_Data [file znad204_supplementary_data.docx]

**Title: Pregnancy and Parenthood in Surgical Training: A Cross-sectional Survey in the United Kingdom**

Authors: Jessica Whitburn^1^, Saiful Miah^2^, Sarah A. Howles^1^

^1^Nuffield Department of Surgical Sciences, University of Oxford, Oxford, OX3 9DU, UK

^2^Department of Urology, Addenbrookes Hospital, Cambridge University Hospitals, Hill Road, Cambridge, CB2 0QQ

**Corresponding author.** Sarah Anne Howles, Nuffield Department of Surgical Sciences, University of Oxford, Oxford, OX3 9DU, UK **ORCID ID**: 0000-0001-6342-0895 **Twitter**: @drsarahhowles

**Supplementary Materials - Index**

| **Supplementary Methods** |  |
| --- | --- |
| Study design and data collection | *page 2* |
| Data analysis | *page 3* |
| **Supplementary Figures and Tables** |  |
| Supplemental Figure 1: Flow chart of survey participants and exclusions | *page 4* |
| **Supplemental Table 1: Time off after pregnancy loss** | *page 4* |
| **Supplemental Table 2: Birth weight of children of surgical trainees** | *page 5* |
| **Supplemental Table 3: Working patterns during pregnancy** | *page 5* |
| **Supplemental Table 4: Parental leave for non-childbearing surgical trainees** | *page 6* |
| **Supplemental Table 5: Return to work after parental leave** | *page 6* |
| **Supplementary Appendixes** |  |
| Internet survey | *page 7* |
|  |  |

**Supplementary Methods**

**Study design and data collection**

A descriptive cross-sectional survey was performed in surgical trainees (residents) in the UK. The methods are reported in accordance with the Checklist for Reporting Results of Internet Surveys^16^. A 192-item survey entitled ‘Pregnancy and Parenthood during Surgical training’ was generated (additional supplementary material). As no existing instrument was validated to evaluate the study aims, questions were developed based on previous surveys and literature review^12,15,17,18^. The resultant survey explores all aspects of pregnancy, childbearing, and parental leave, including information regarding level of training and working hours. Answers were recorded using a combination of binominal, multiple choice, and Likert scales. No qualitative data was collected. The survey was tested in two male and four female surgeons in the local region and two rounds of revisions made based on feedback. The web-based survey tool, JISC (2022, Bristol, UK), was used to administer the questionnaire, online completion was facilitated via a weblink.

The survey was distributed via a nationally recognised/endorsed surgical specialty trainee group (British Association of Urological Surgeons Section of Trainees) and publicised on Twitter via the ‘Pregnancy and parenthood in surgical training’ account; reminders to complete the survey were posted on the Twitter account in July, September, October and November. The survey was open for data collection from 24^th^ June – 24^th^ December 2022. All surgical trainees working in the UK were invited to participate. Individuals who were not in surgical training were excluded to increase homogeneity of working practices respondents were exposed to. If a surgeon was a parent but not themselves childbearing, they were asked to answer questions regarding their partner’s pregnancies. These individuals served as a socio-demographically similar control group for pregnancy outcomes and are referred to as non-surgeon partners within the study. Male surgeons with female surgeon partners in training were excluded from analysis to prevent the same data being captured twice. Data was visually inspected for potential double entries, no data was excluded on this basis. Participation was voluntary and uncompensated; the participant information leaflet explained that survey completion would act as implied consent. Individual trainees were not identifiable from the survey. Eligibility for inclusion was based solely on participant answers, no checks were made to confirm that participants were surgical trainees. Ethical approval was gained via the Central University Research Ethics Committee, University of Oxford (Ref: R80778/RE001).

Minor pregnancy-associated complications were defined as hyperemesis, musculoskeletal issues, gestational diabetes, and gastro-oesophageal reflux. Major pregnancy-associated complications were defined as pre-eclampsia or hypertension, placental abruption or bleeding in pregnancy, placenta praevia or accrete, intrauterine growth restriction (IUGR), and placental insufficiency (including oligohydramnios). Parental leave is defined as time taken off work to look after a child; in the UK, pregnant childbearing parents are entitled to 52 weeks of leave, non-childbearing parents may have the right to leave when a partner/surrogate is having a baby or child is being adopted.

**Data analysis**

Data were analysed in GraphPad Prism (version 9.5.0, California, USA). Significance testing was performed using χ^2^ or Fishers exact test for non-parametric binary data and Student t test for continuous variables. All tests of significance were 2-tailed. Pregnancy loss rate was calculated using the formula: (total number of pregnancy losses/total number of pregnancies) x 100.

Data was not available regarding the ages of non-surgeon partners at time of pregnancy loss.

**Supplementary Figures and Tables**

**Supplemental Figure 1: Flow chart of survey participants and exclusions**

*Data from https://bmjopen.bmj.com/content/12/2/e055516 and https://www.rcog.org.uk/media/wuobyggr/rcog-workforce-report-2022.pdf . All questionnaires were completed to the end, individual data gaps were recorded as ‘unknown’.

**Supplemental Table 1: Time off after pregnancy loss**

|  | **Childbearing surgeon, n (%)** |
| --- | --- |
| **Time off after pregnancy loss** | |
| No time off | 29 (33) |
| 1-7 days | 37 (43) |
| 1-2 weeks | 14 (16) |
| 2-3 weeks | 0 (0) |
| 3-4 weeks | 5 (6) |
| >4 weeks | 2 (2) |
| **I felt my colleagues were supportive of time off after pregnancy loss** | |
| Strongly agree | 8 (9) |
| Agree | 24 (28) |
| I didn’t tell them | 46 (53) |
| Disagree | 6 (7) |
| Strongly disagree | 3 (3) |
| **I felt my supervisors/training programme director were supportive of time off after pregnancy loss** | |
| Strongly agree | 14 (16) |
| Agree | 22 (25) |
| I didn’t tell them | 41 (47) |
| Disagree | 7 (8) |
| Strongly disagree | 3 (3) |

**Supplemental Table 2: Birth weight of children of surgical trainees**

|  | **Childbearing surgeon, n (%)** | **Non-surgeon partner, n (%)** | **p value** |
| --- | --- | --- | --- |
| **Birth weight** | | | |
| <2.5kg | 10 (4) | 6 (8) | 0.21 |
| 2.5-3kg | 54 (19) | 15 (22) |  |
| 3-4kg | 188 (66) | 41 (59) |  |
| >4kg | 30 (11) | 5 (7) |  |
| No answer | 1 (0) | 2 (3) |  |

Insufficient data to allow statistical analysis of birth weight stratified by birth order. Data stratified by gender not available.

**Supplemental Table 3: Working patterns during pregnancy**

|  | **Childbearing surgeon, n (%)** | **Non-surgeon partner, n (%)** | **p value** |
| --- | --- | --- | --- |
| **Average hours worked per week** | | | |
| <40 | 78 (28) | 33 (48) | 0.003 |
| 40-60 | 190 (67) | 36 (52) |  |
| >60 | 10 (4) | 0 |  |
| No answer | 5 (2) | 0 |  |
| **Frequency of overnight on call/nightshifts** | | | |
| None | 53 (19) |  |  |
| 2-4/month | 147 (52) |  |  |
| 4-6/month | 66 (23) |  |  |
| >6/month | 15 (5) |  |  |
| No answer | 2 (1) |  |  |
| **Hours operating last trimester** | | | |
| 0-8 | 140 (49) |  |  |
| 9-12 | 75 (27) |  |  |
| 13-16 | 41 (14) |  |  |
| >16 | 26 (9) |  |  |
| No answer | 1 (0.4) |  |  |
| **Average hours worked per week in last trimester** | | | |
| <30 | 78 (28) |  |  |
| 30-40 | 96 (34) |  |  |
| 41-60 | 105 (37) |  |  |
| >60 | 2 (1) |  |  |
| No answer | 2 (1) |  |  |

**Supplemental Table 4: Parental leave for non-childbearing surgical trainees**

|  | **Non-childbearing surgeon, n (%)** |
| --- | --- |
| **Parental leave (weeks)** | |
| None | 2 (5) |
| 1-2 | 31 (76) |
| 3-8 | 4 (10) |
| >8 | 3 (7) |
| No answer | 1 (2) |
| **Was it easy to arrange parental leave?** | |
| Yes, no difficulties | 15 (37) |
| Some difficulties but organised to my satisfaction | 13 (32) |
| No, it was not easy to arrange | 9 (22) |
| N/A | 3 (7) |
| No answer | 1 (2) |

**Supplemental Table 5: Return to work after parental leave**

|  | **Childbearing surgeon, n (%)** | **Non-childbearing surgeon, n (%)** |
| --- | --- | --- |
| **Changes to work pattern on return to work** | | |
| No change, happy with work schedule | 29 (17) | 21 (51) |
| No change, unhappy with work schedule | 17 (10) | 9 (22) |
| Altered work schedule, ≥ 40 hours/week | 18 (11) | 4 (10) |
| Altered work schedule, <40 hours/week | 104 (61) | 6 (15) |
| No answer | 3 (2) | 1 (2) |
| **If working <40 hours, what % worked** | | |
| 50% | 3 (3) | 1 (17) |
| 60% | 40 (38) | 1 (17) |
| 70% | 6 (6) | 0 (0) |
| 80% | 47 (45) | 4 (67) |
| 90% | 5 (5) | 0 (0) |
| Not confirmed | 1 (1) | 0 (0) |
| No answer | 1 (2) | 0 (0) |

**
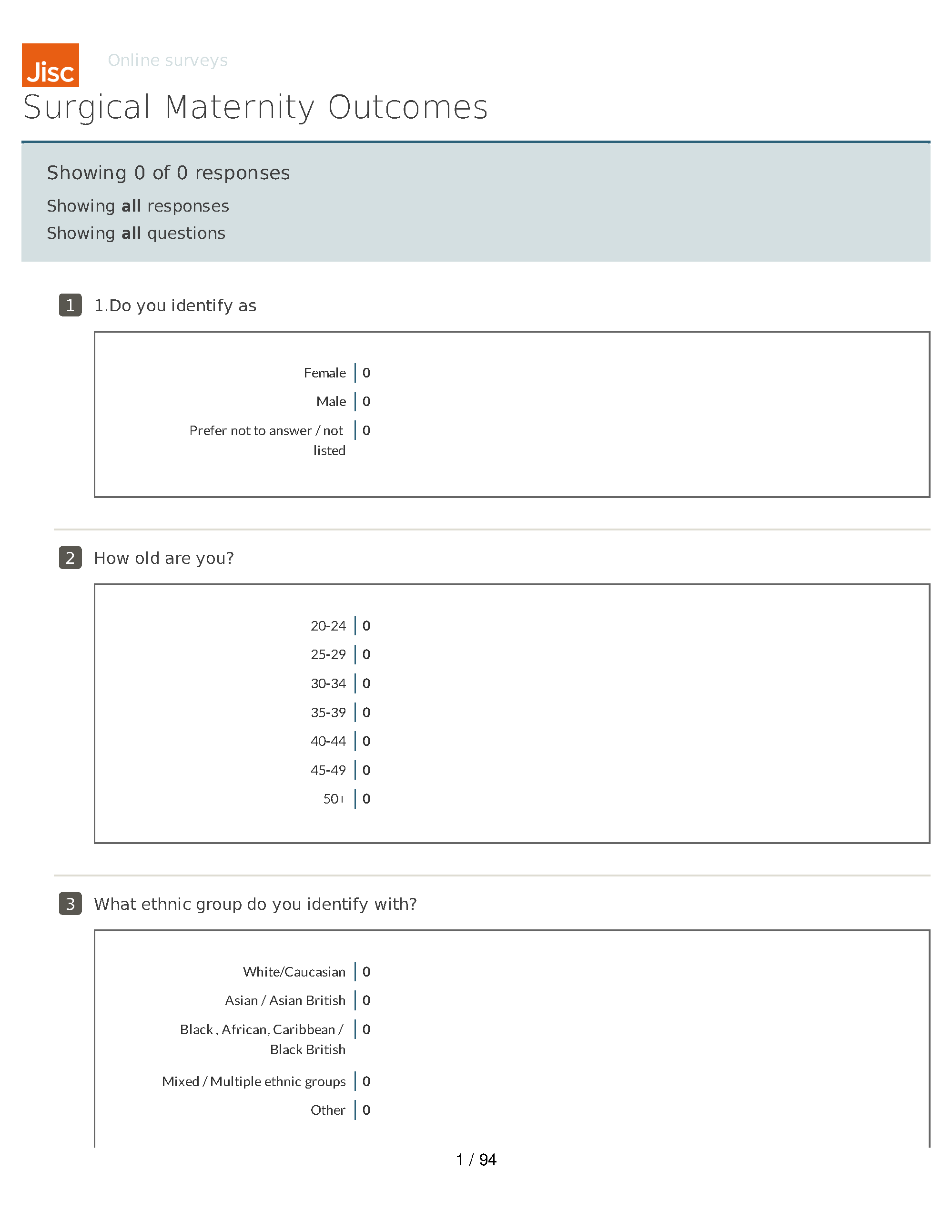
**

**
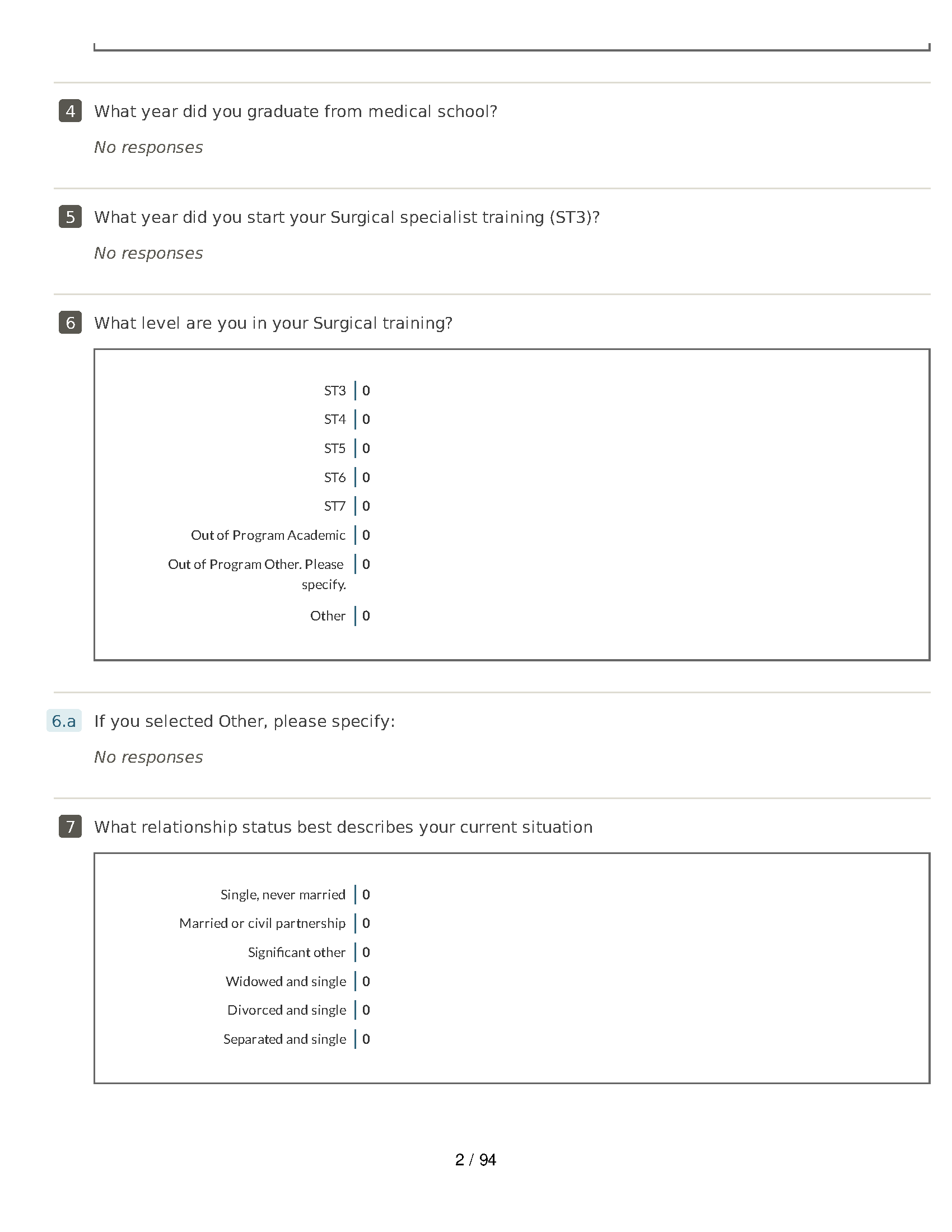

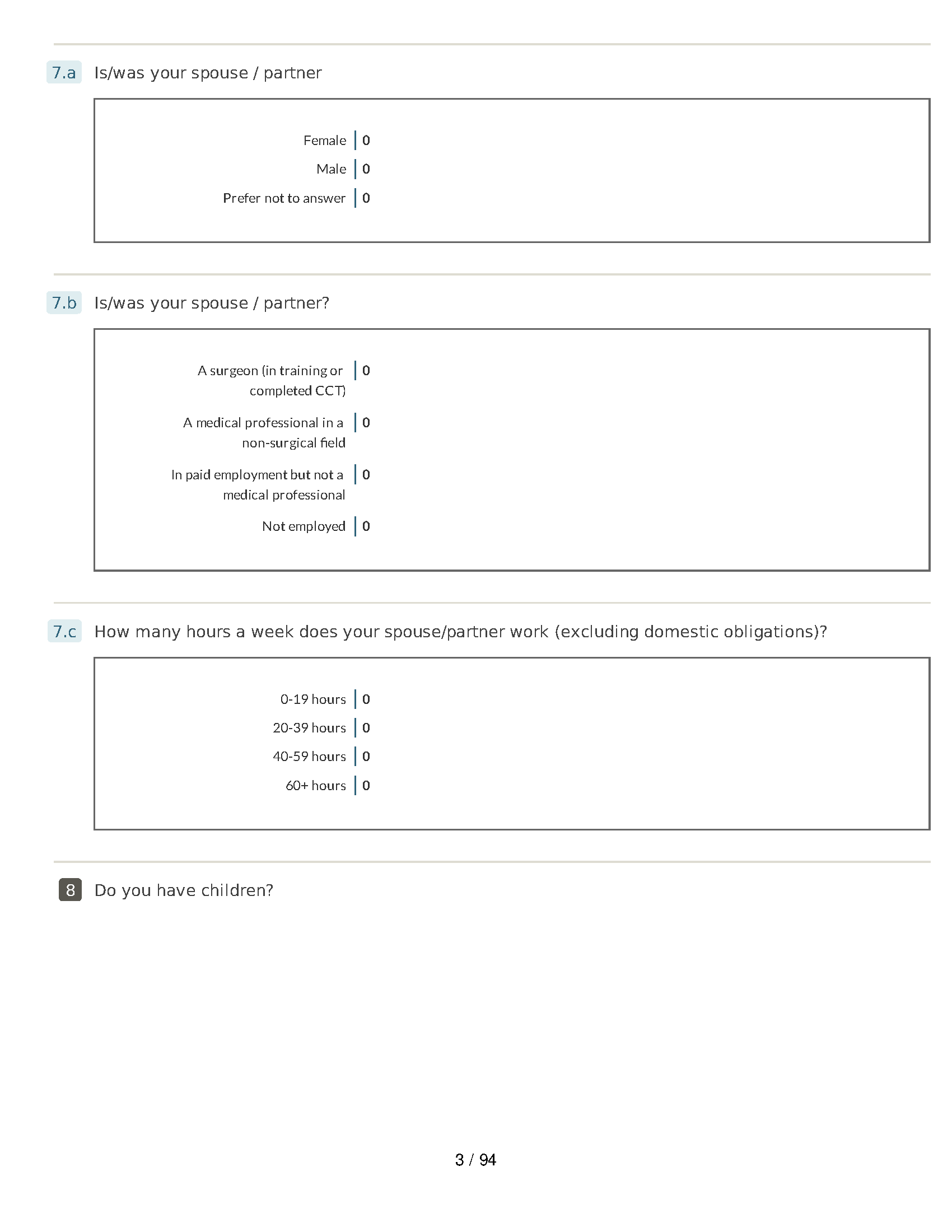

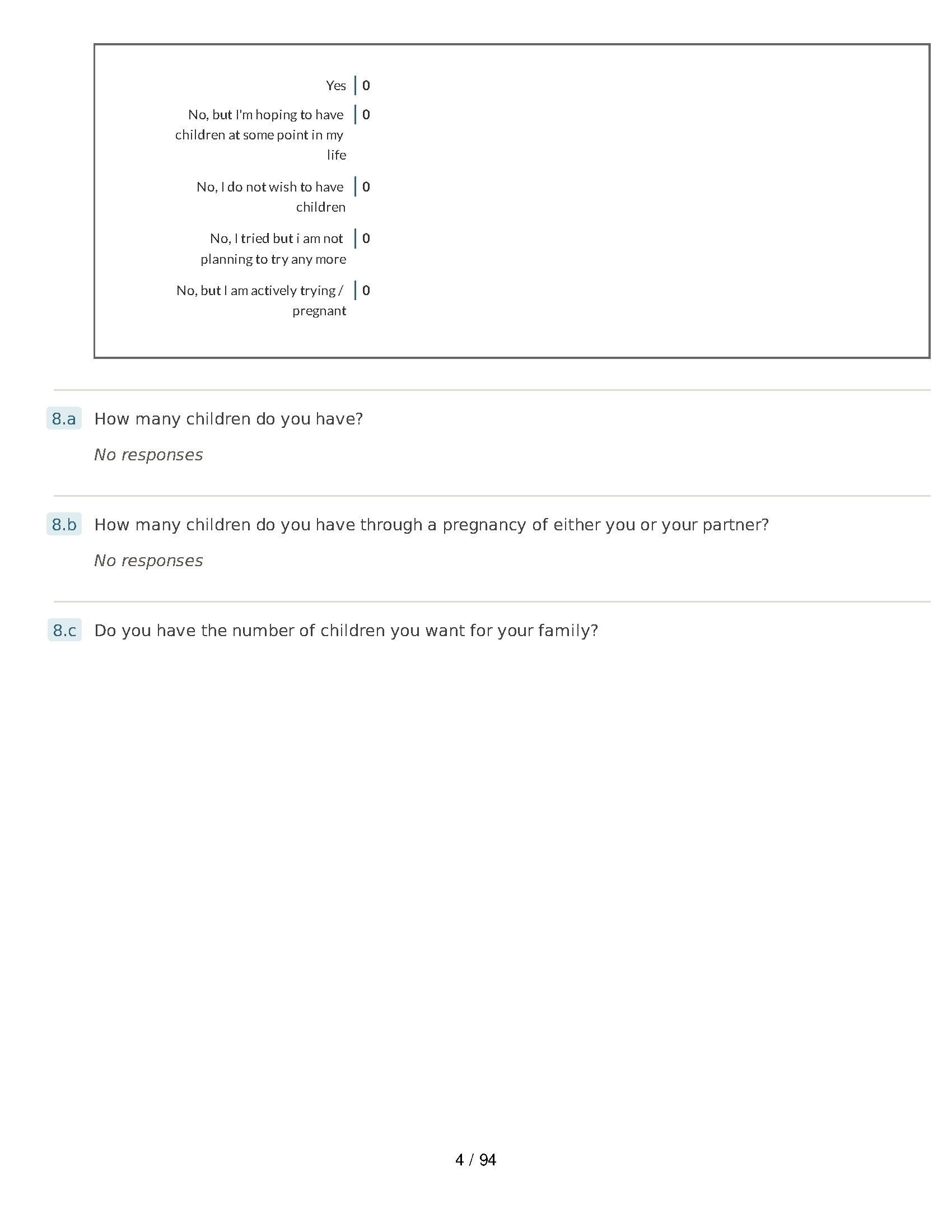

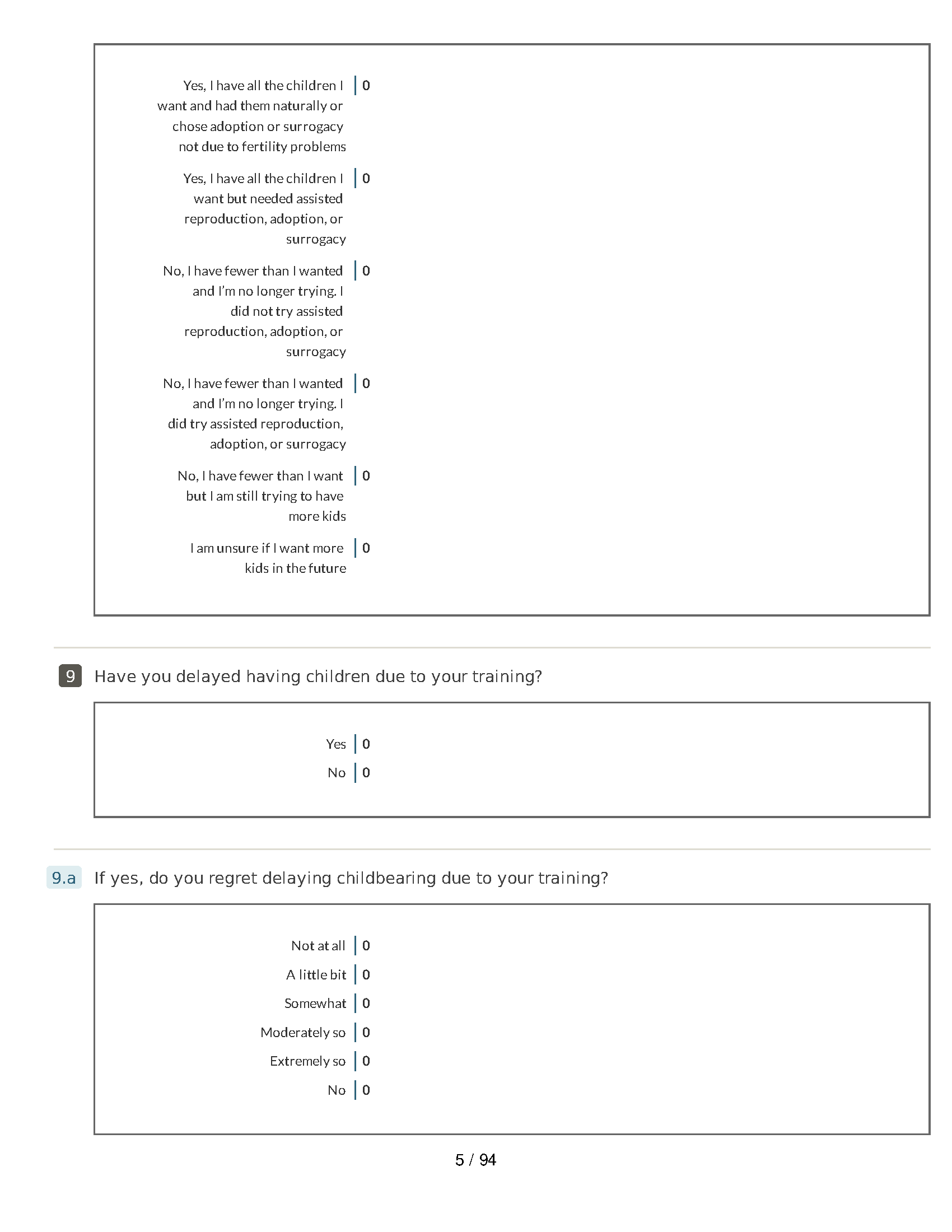

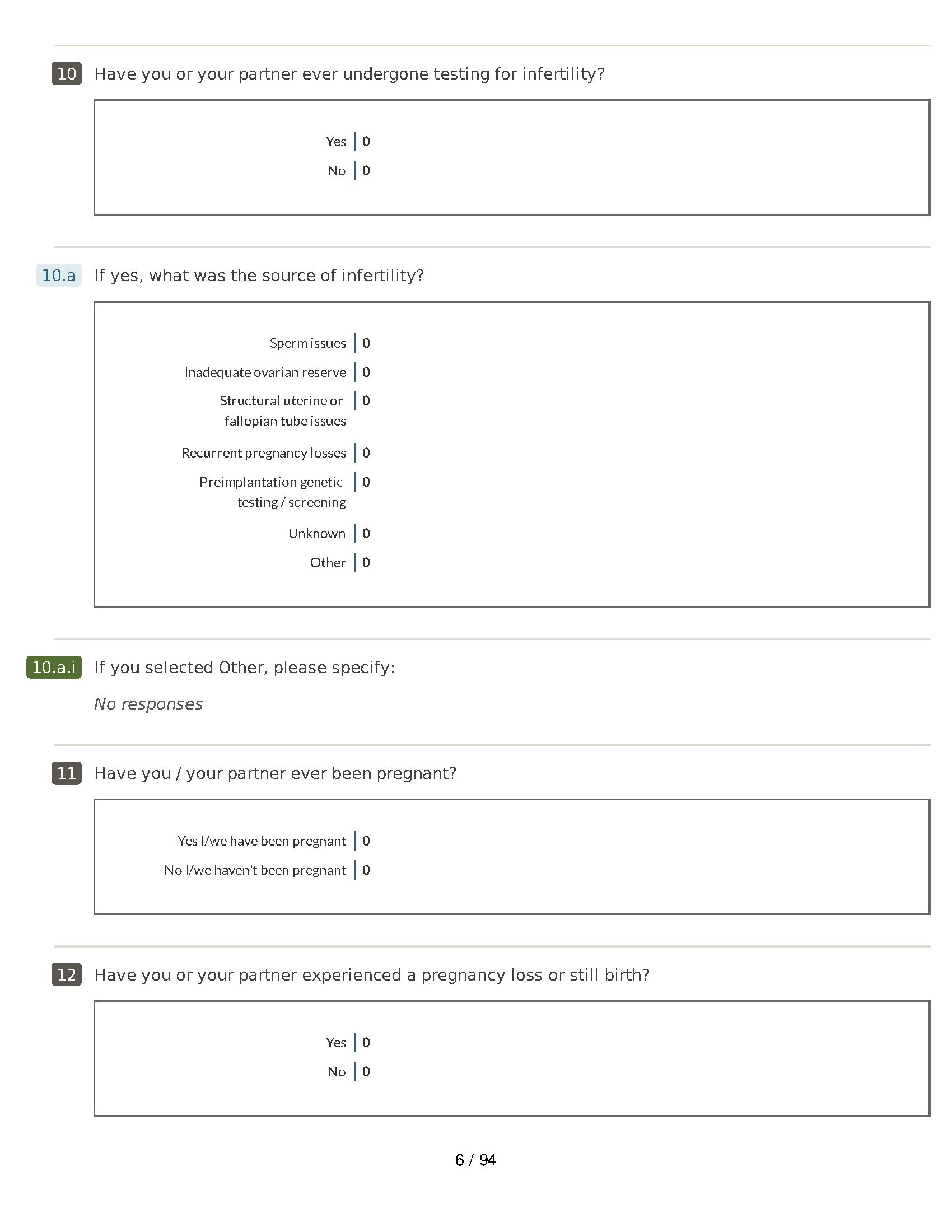

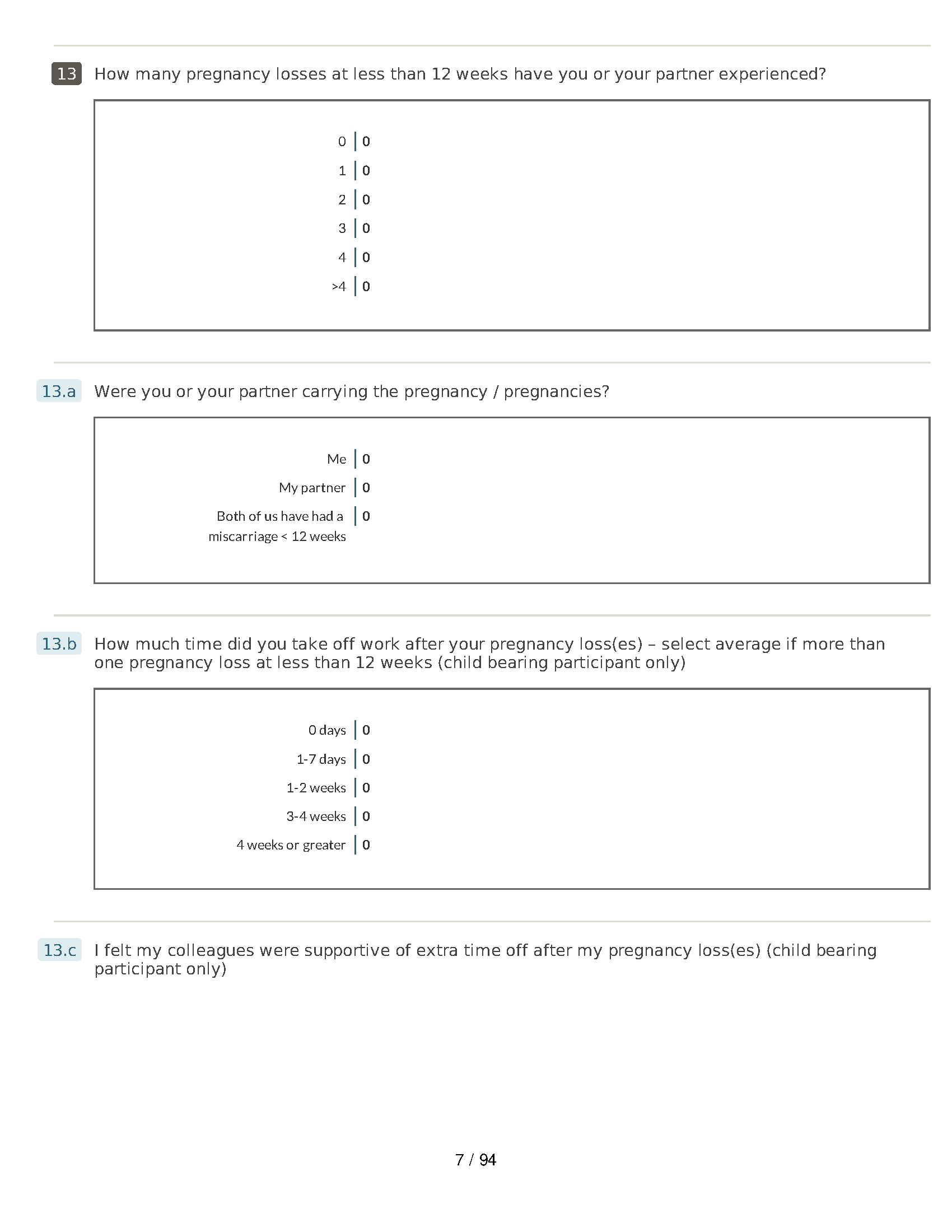

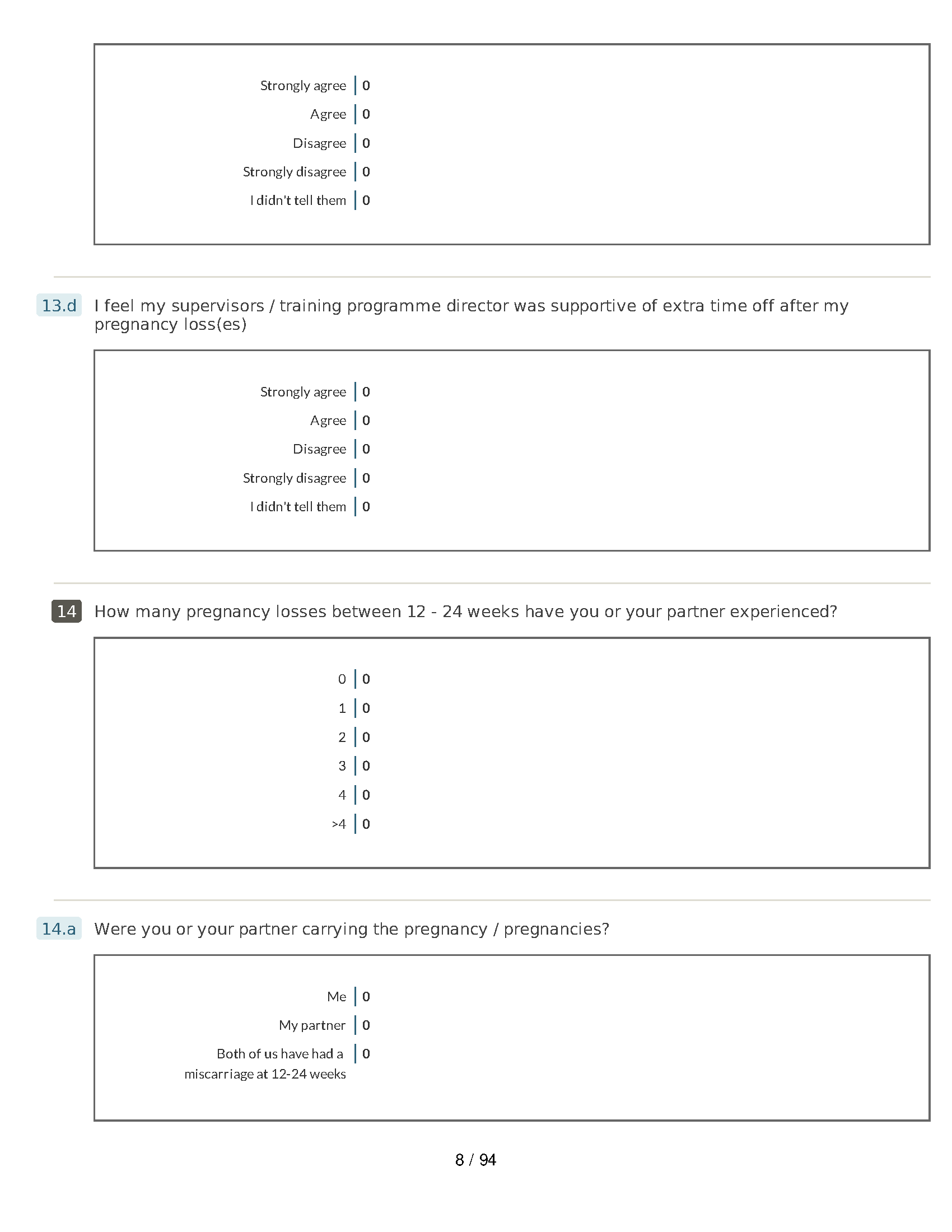

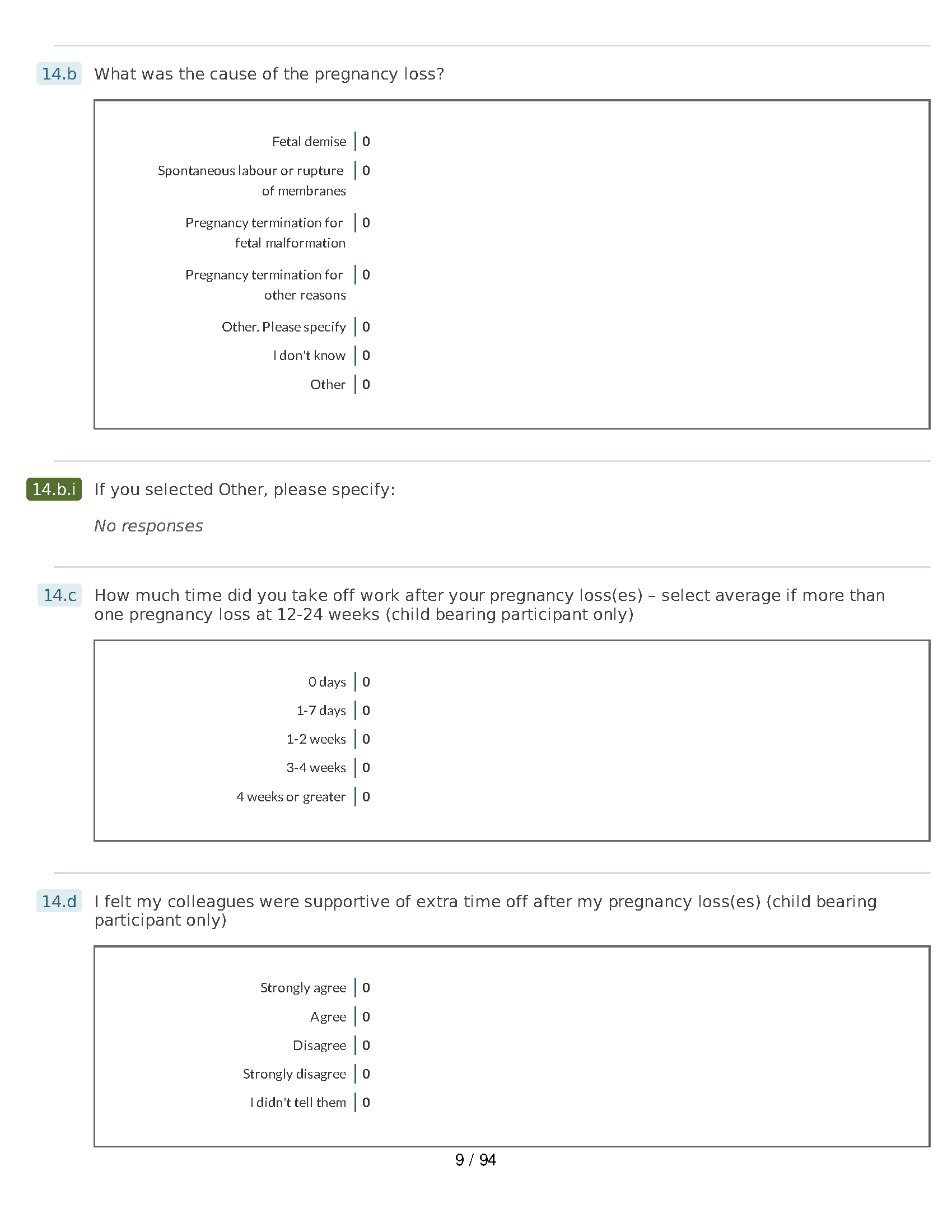

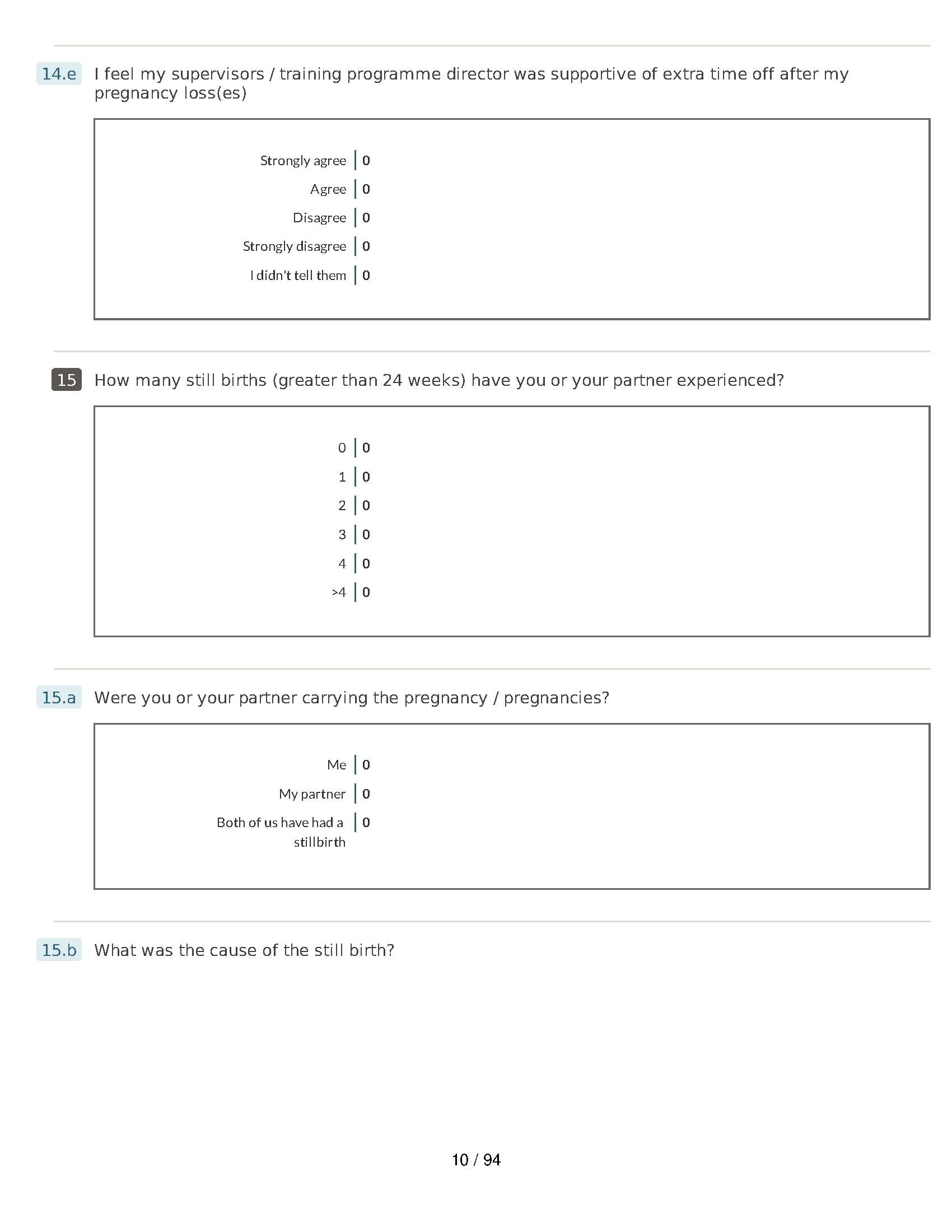
**

**
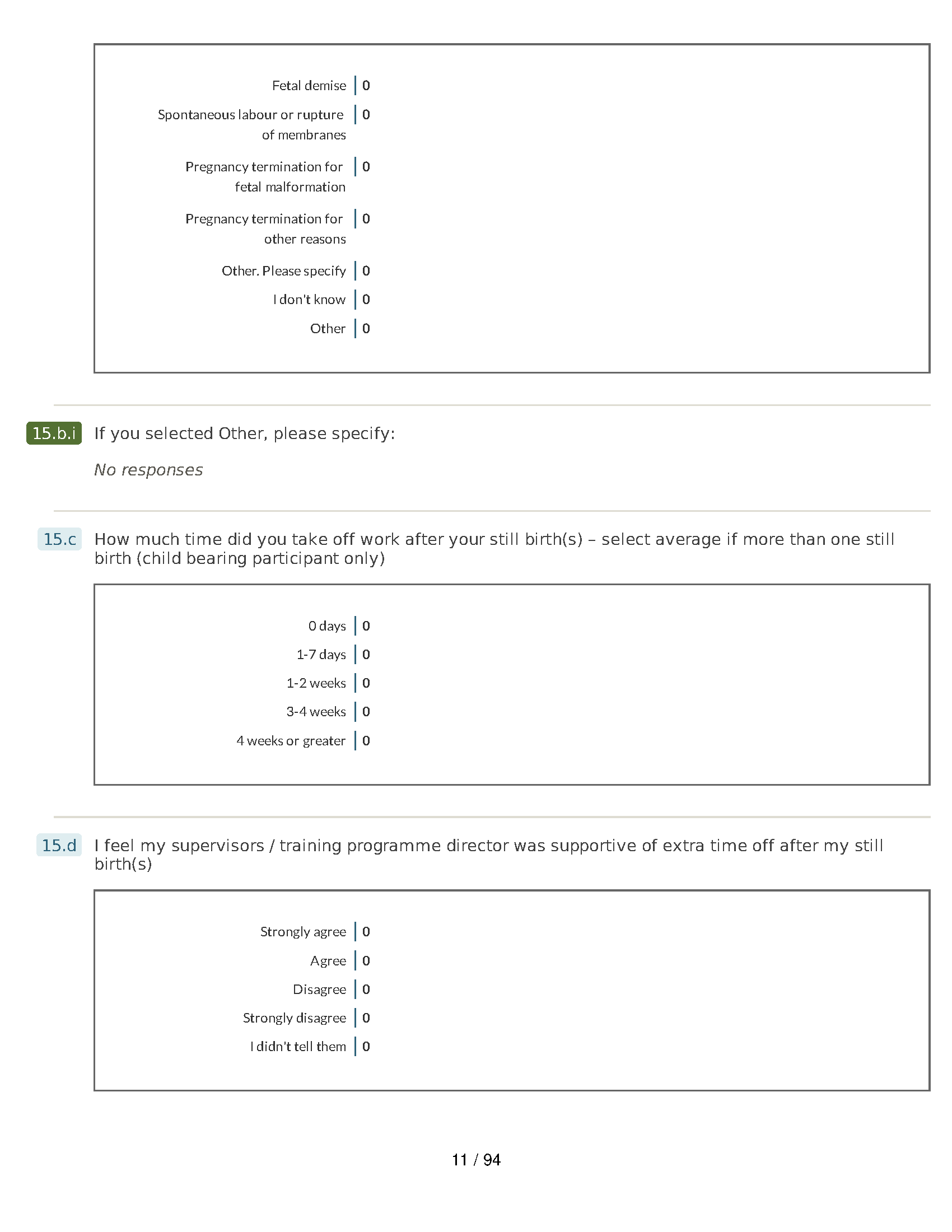

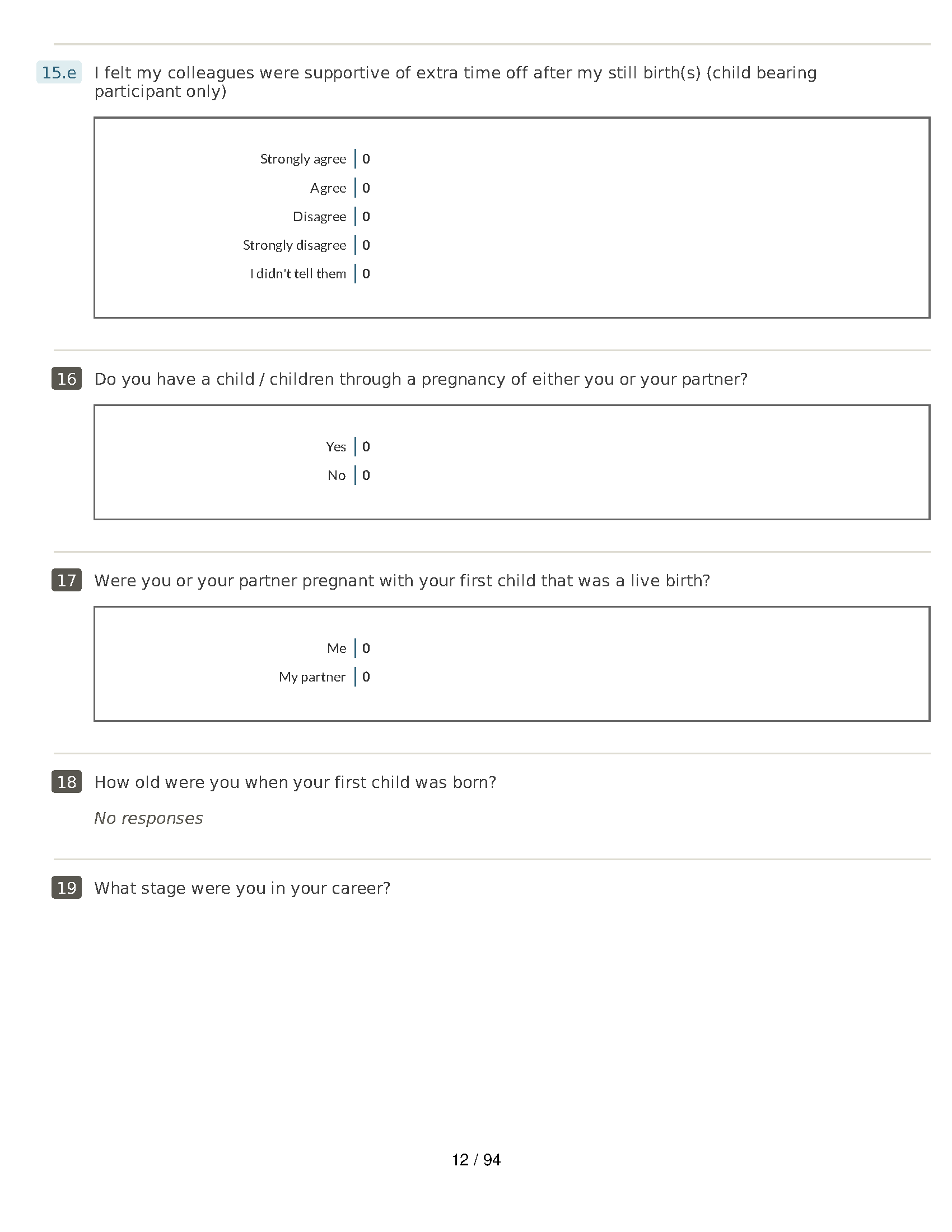

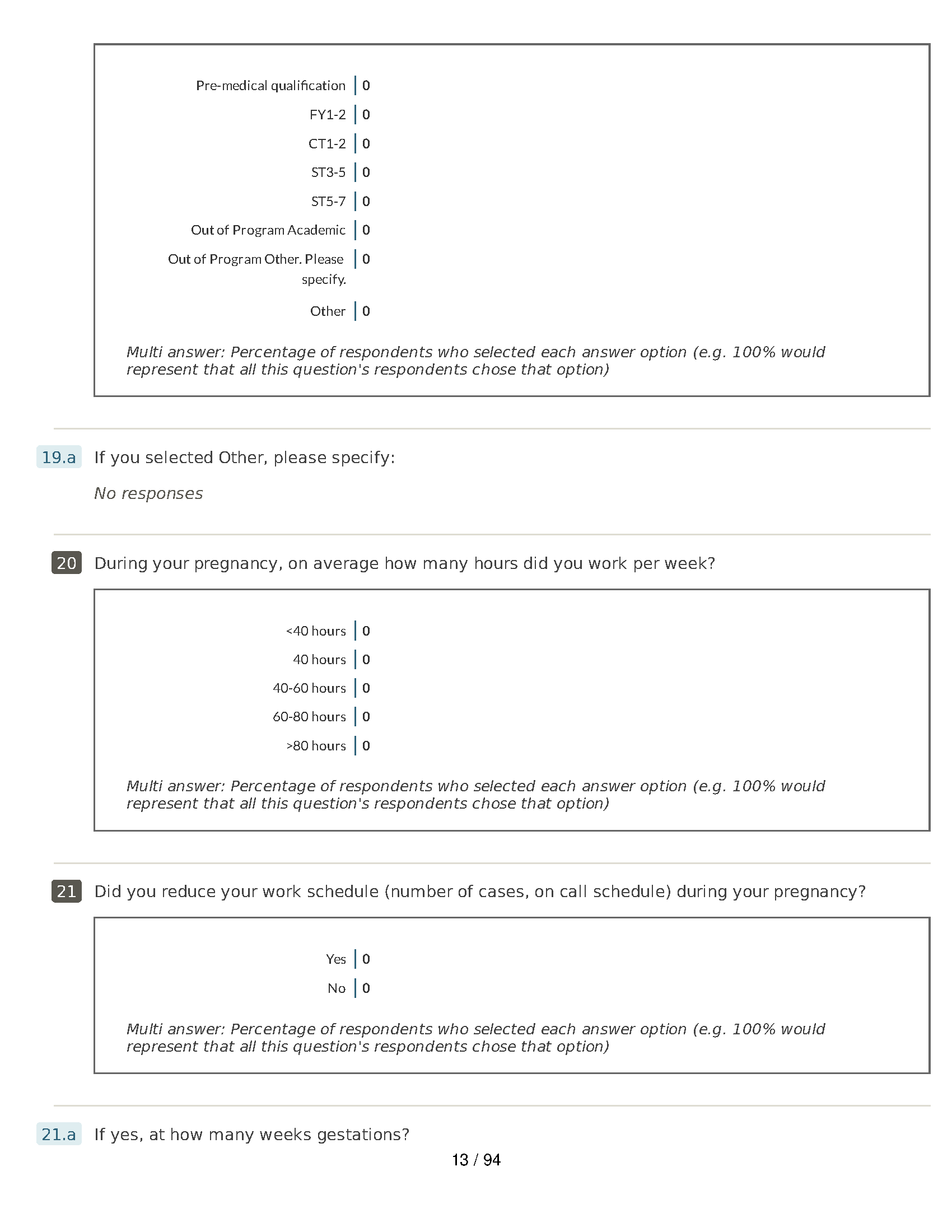

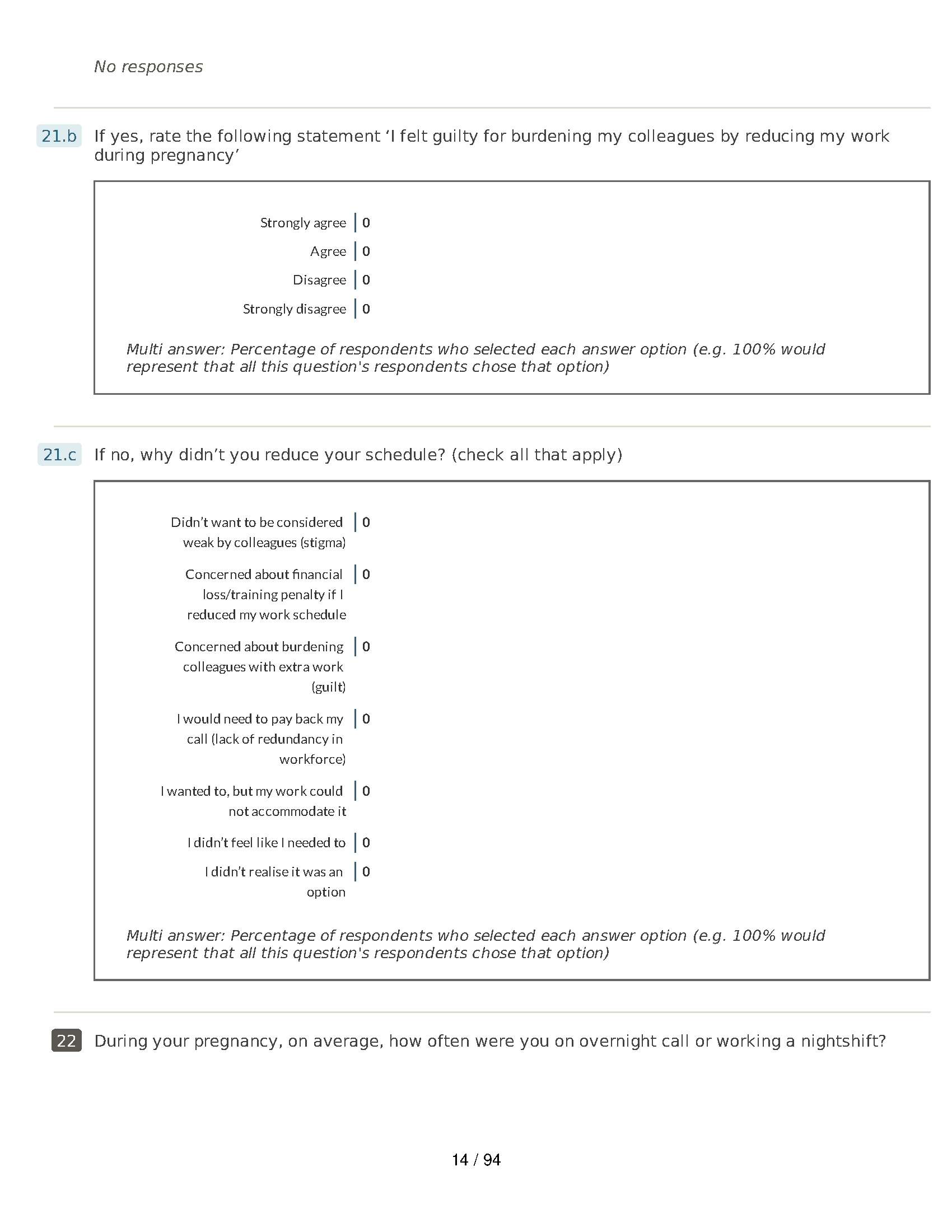

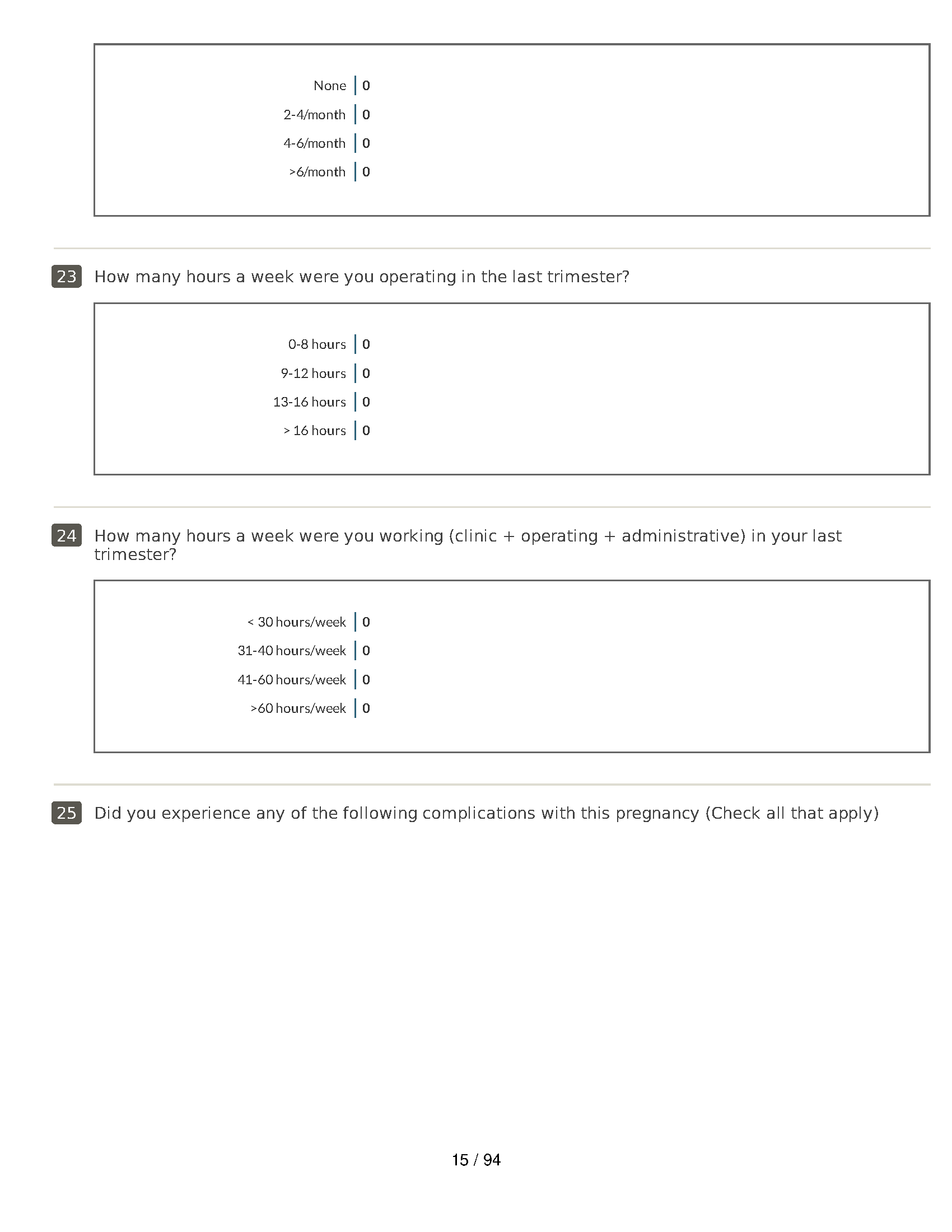

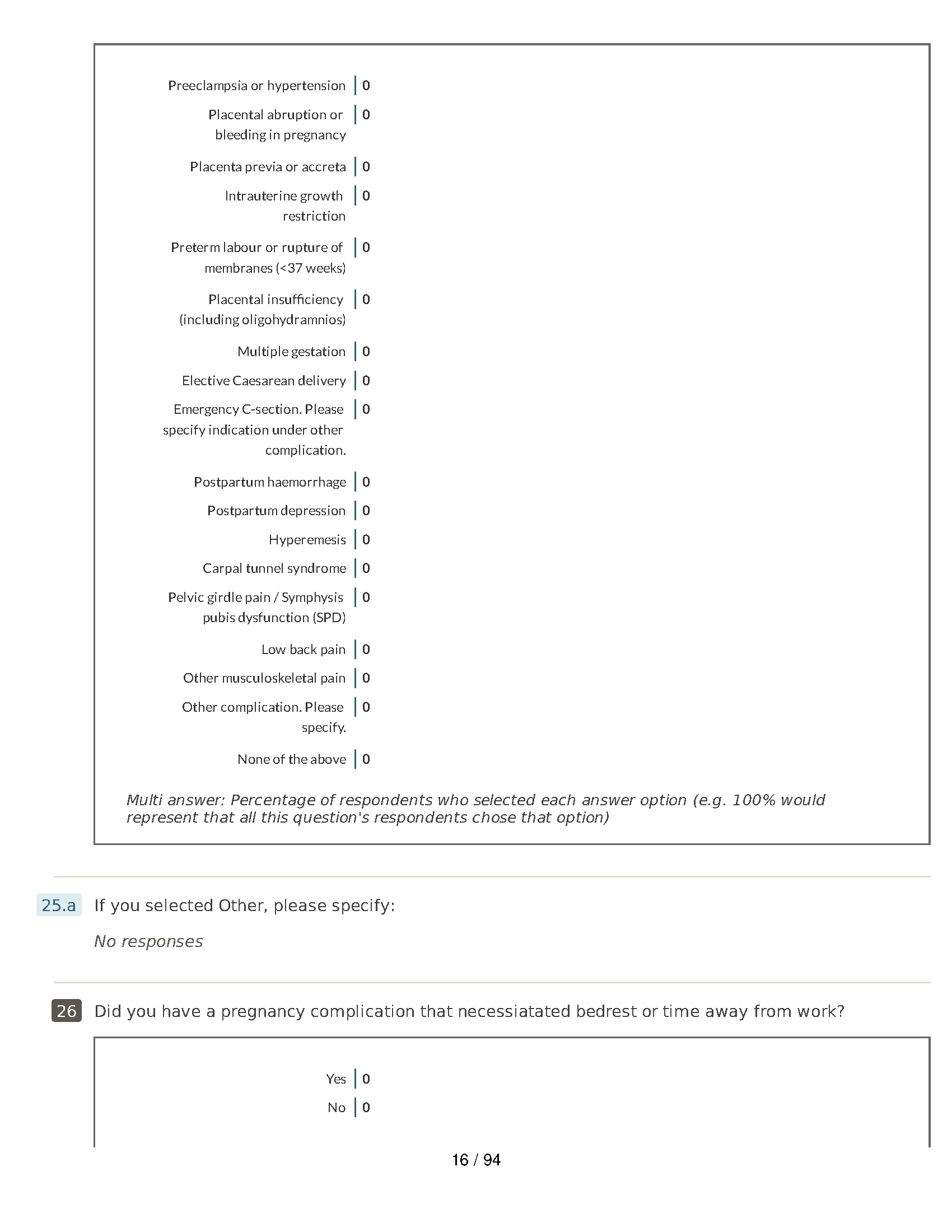

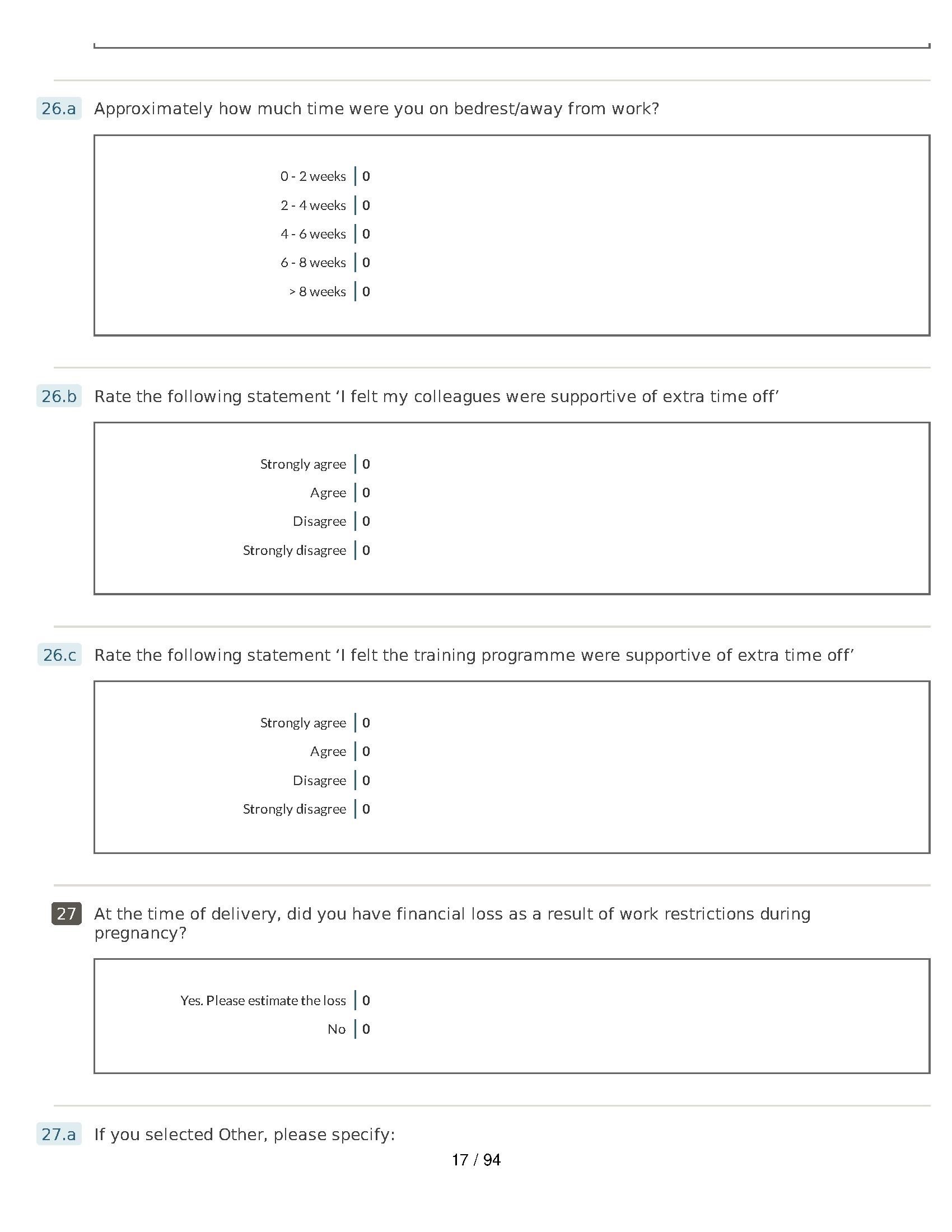

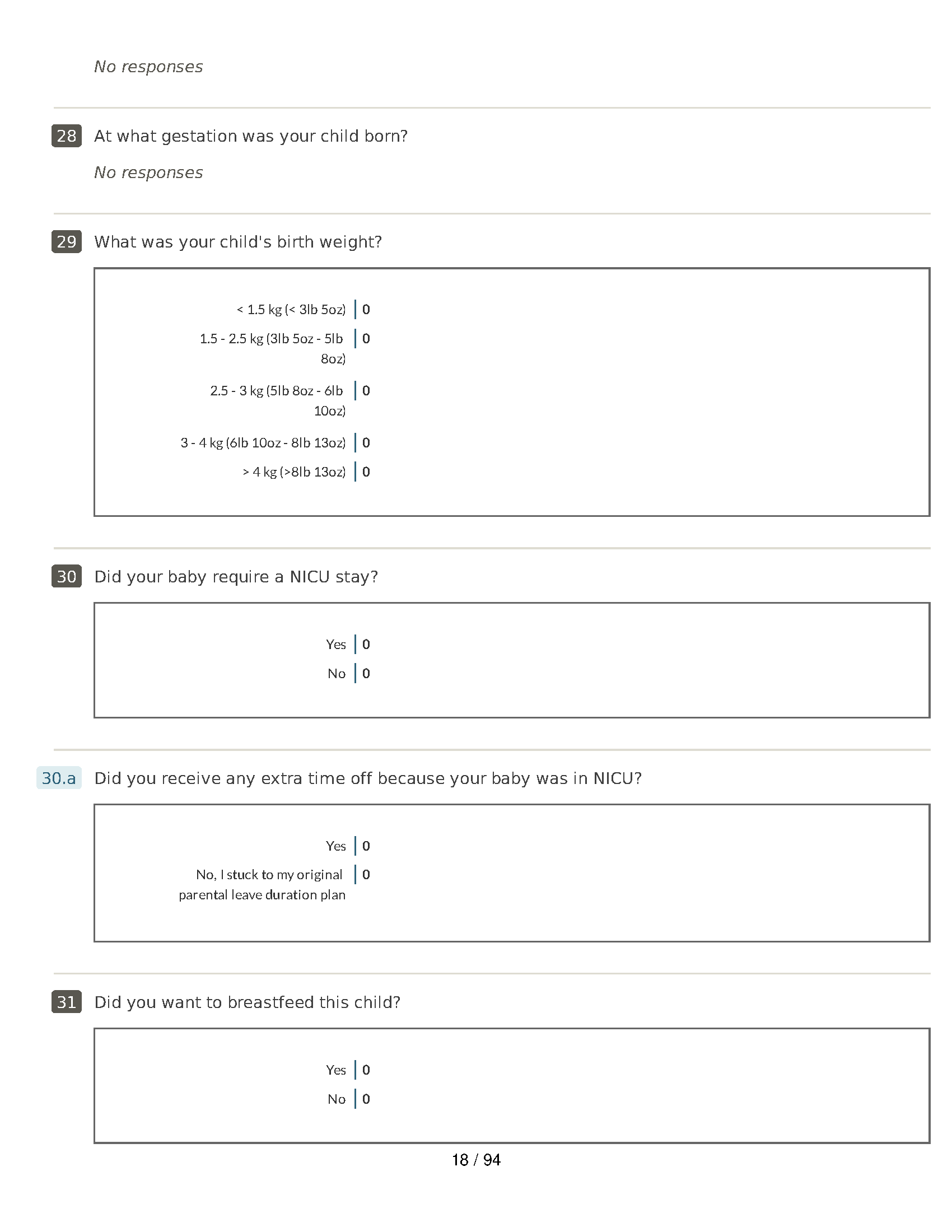

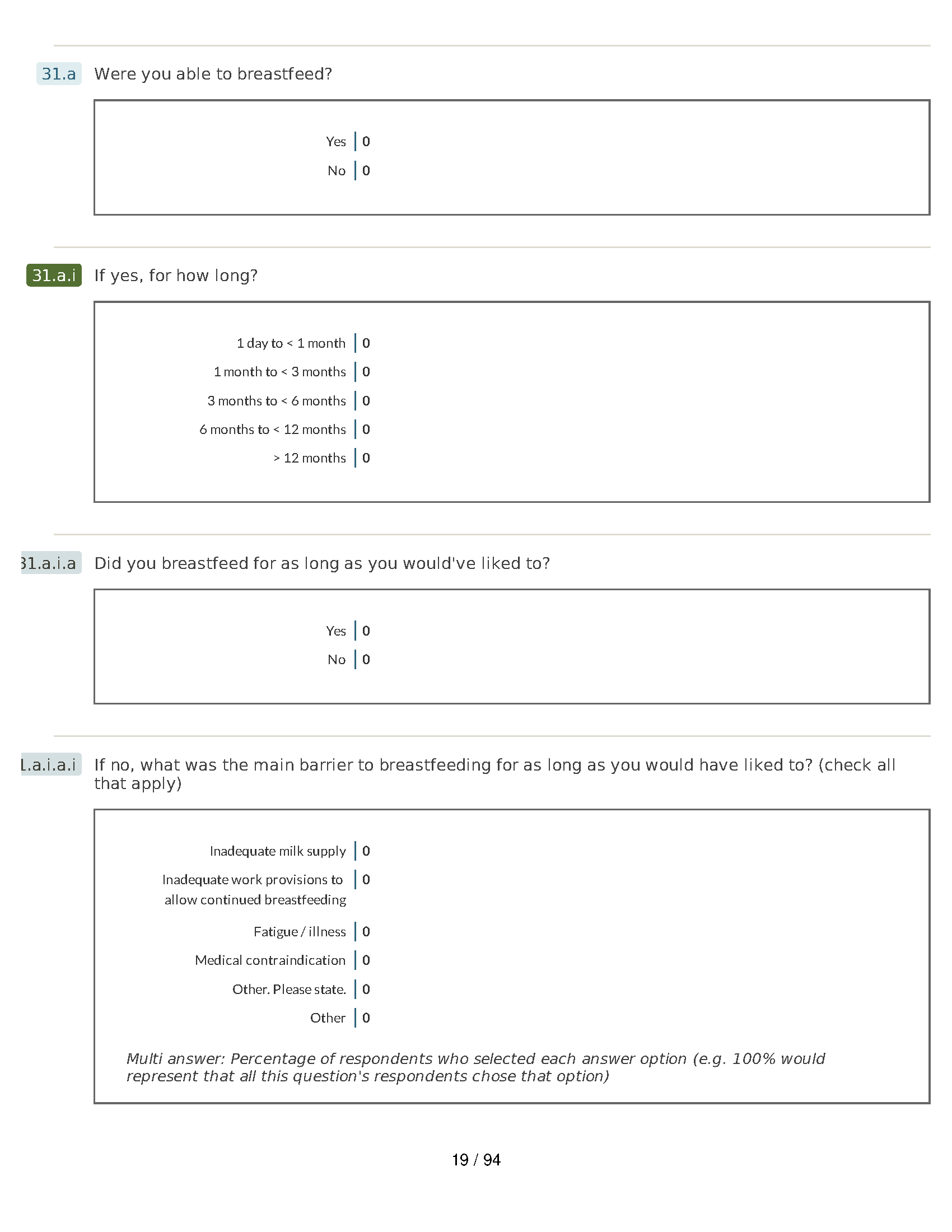

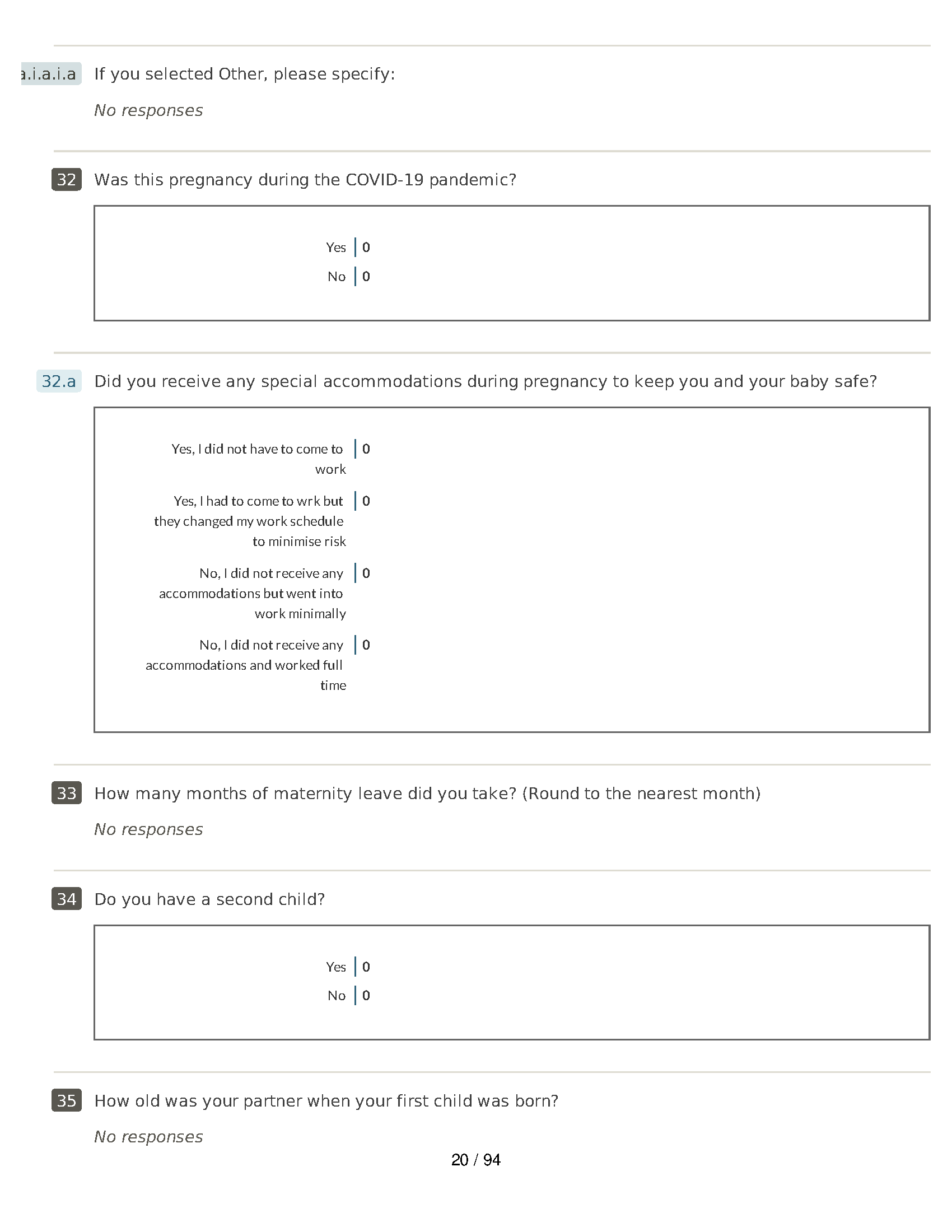

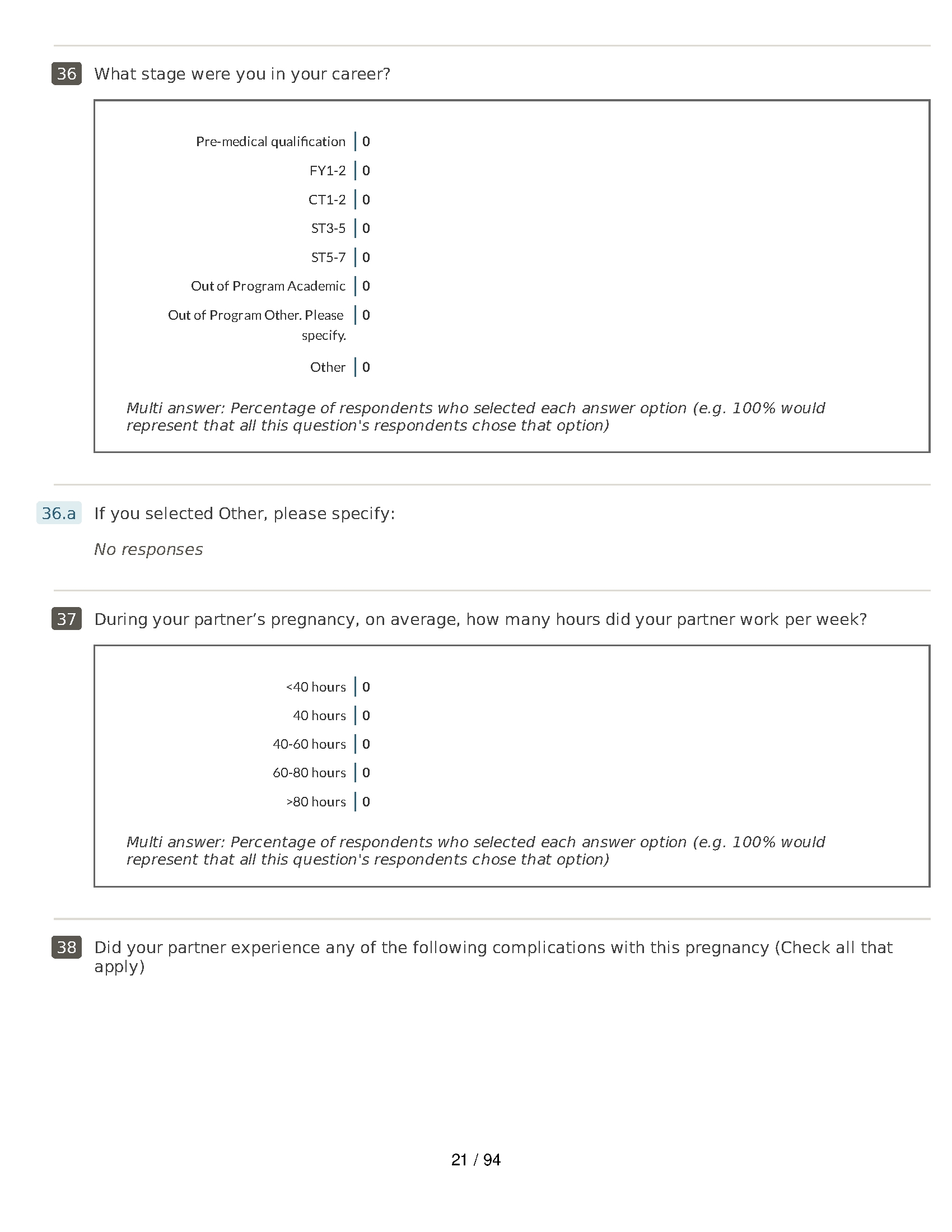

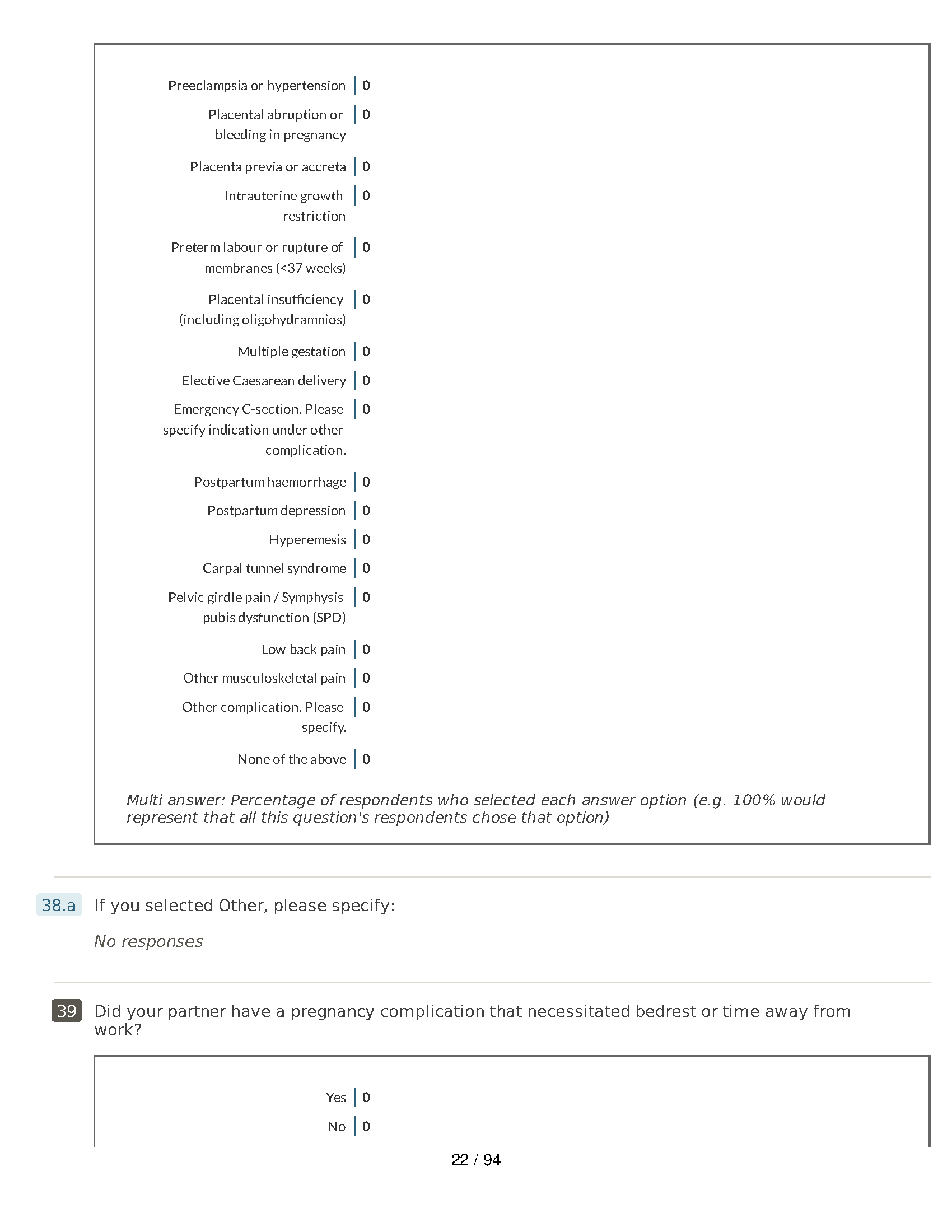

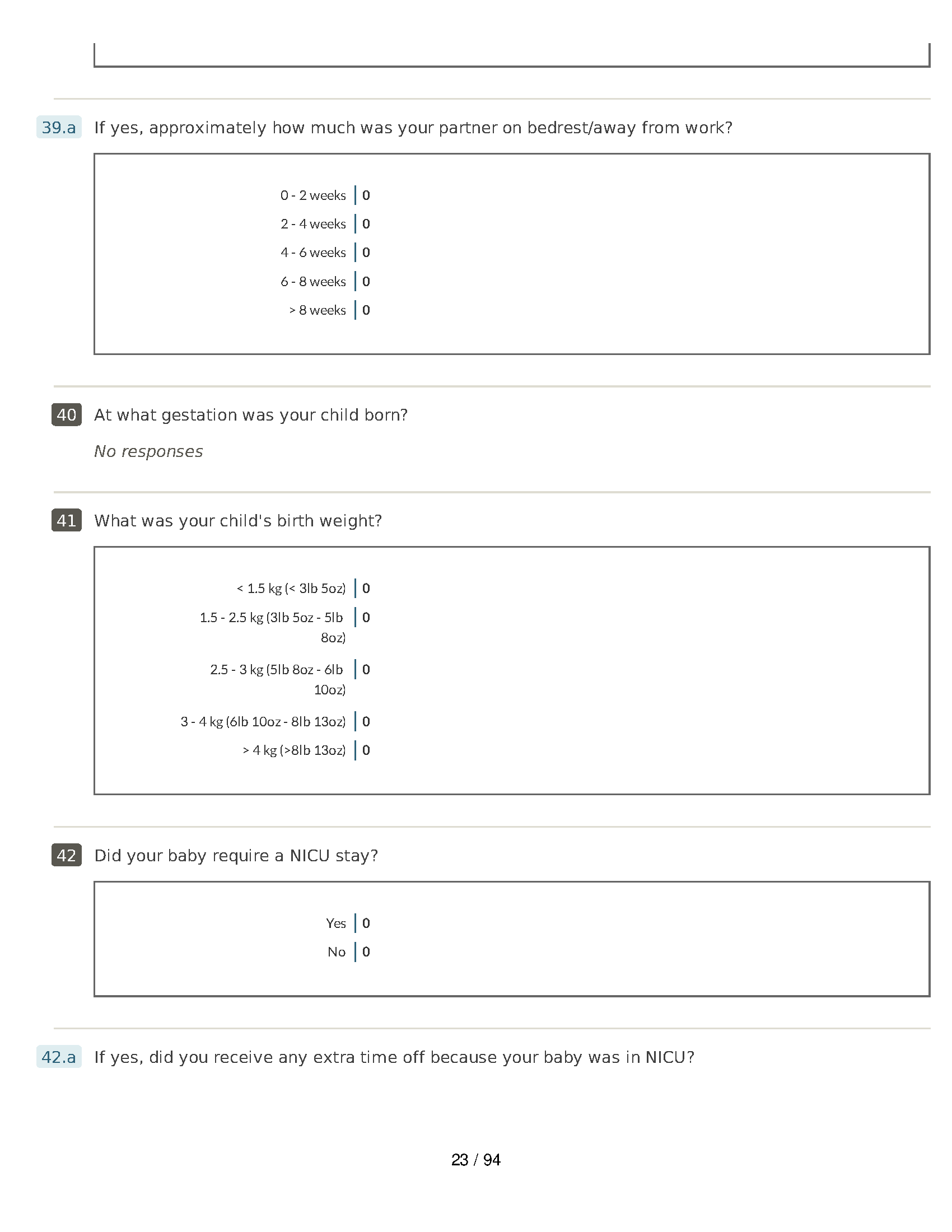

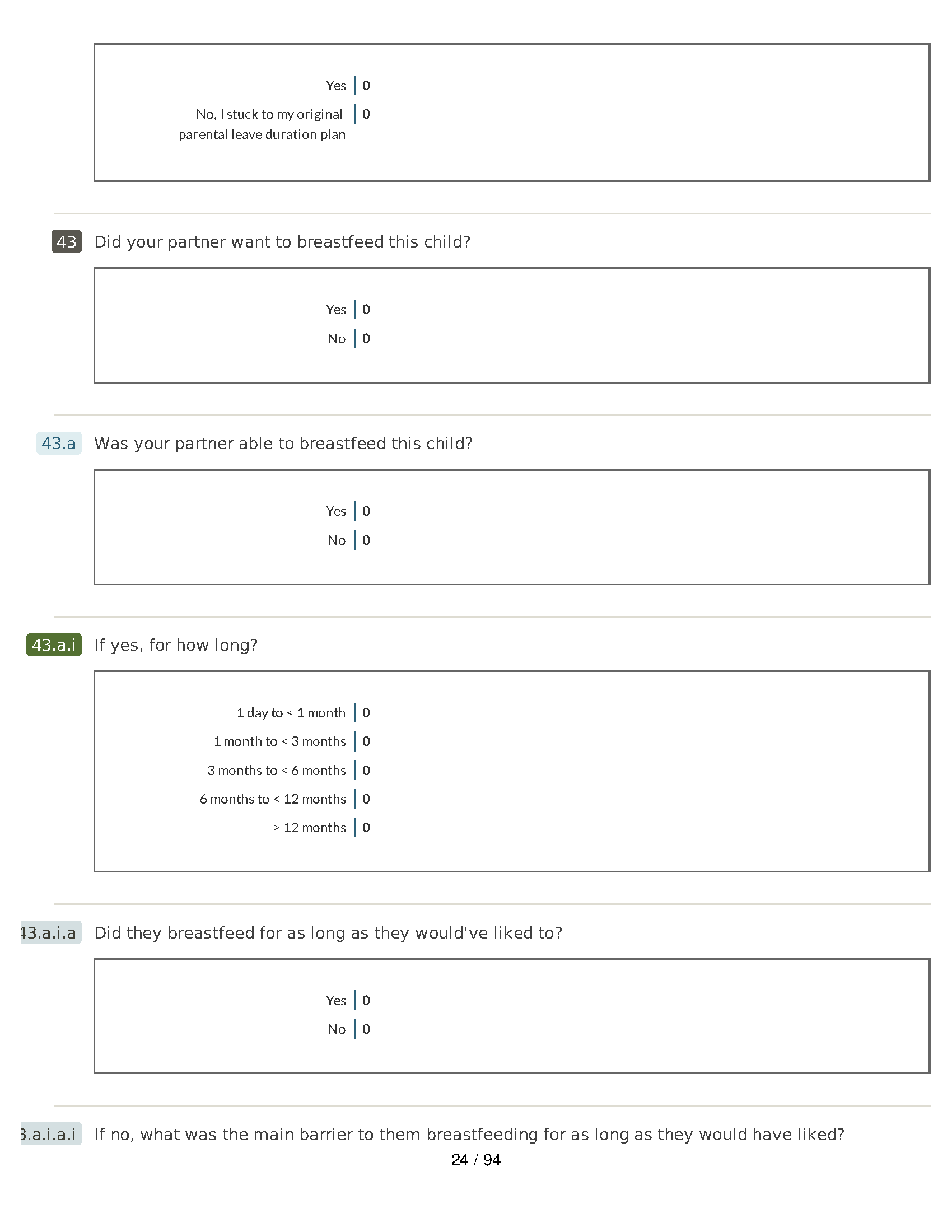

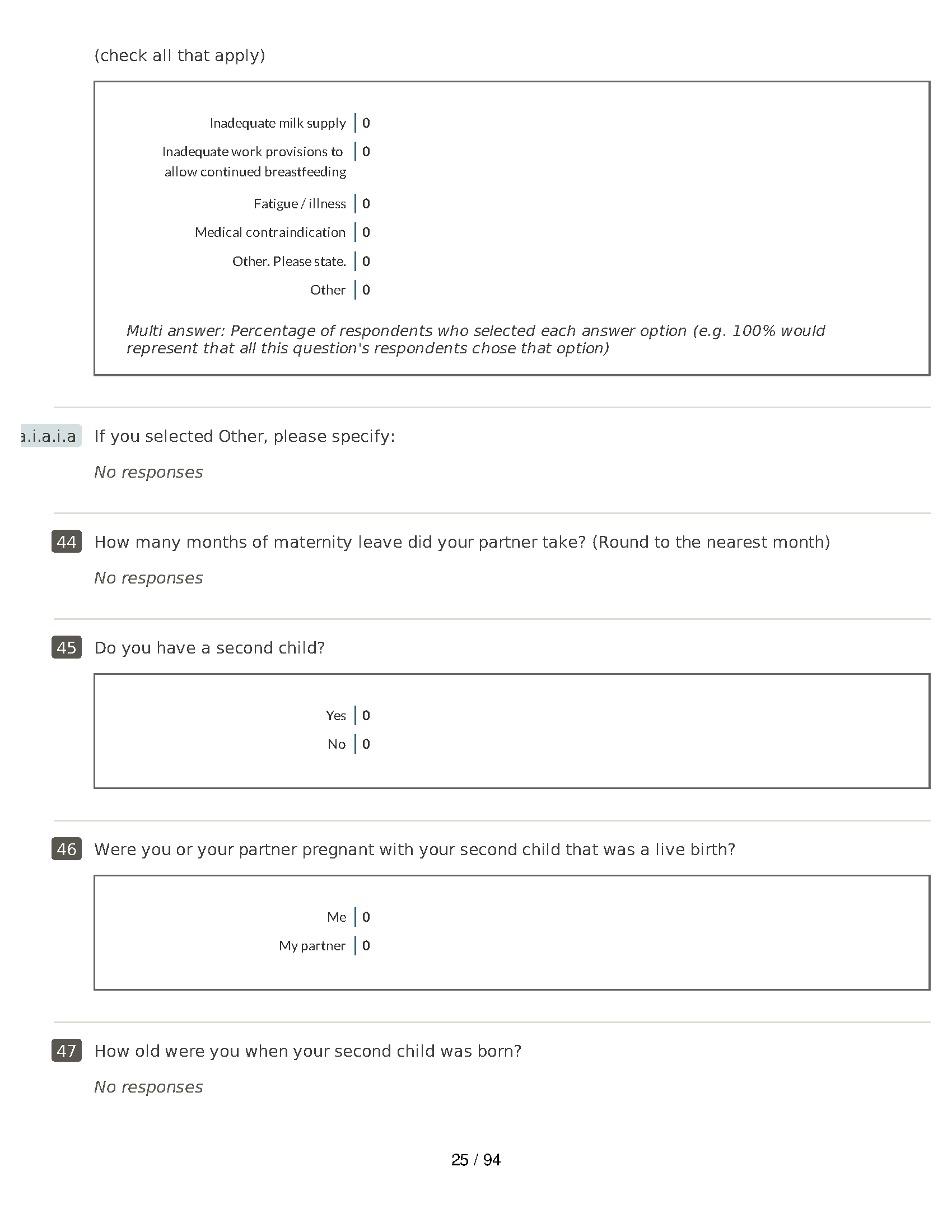

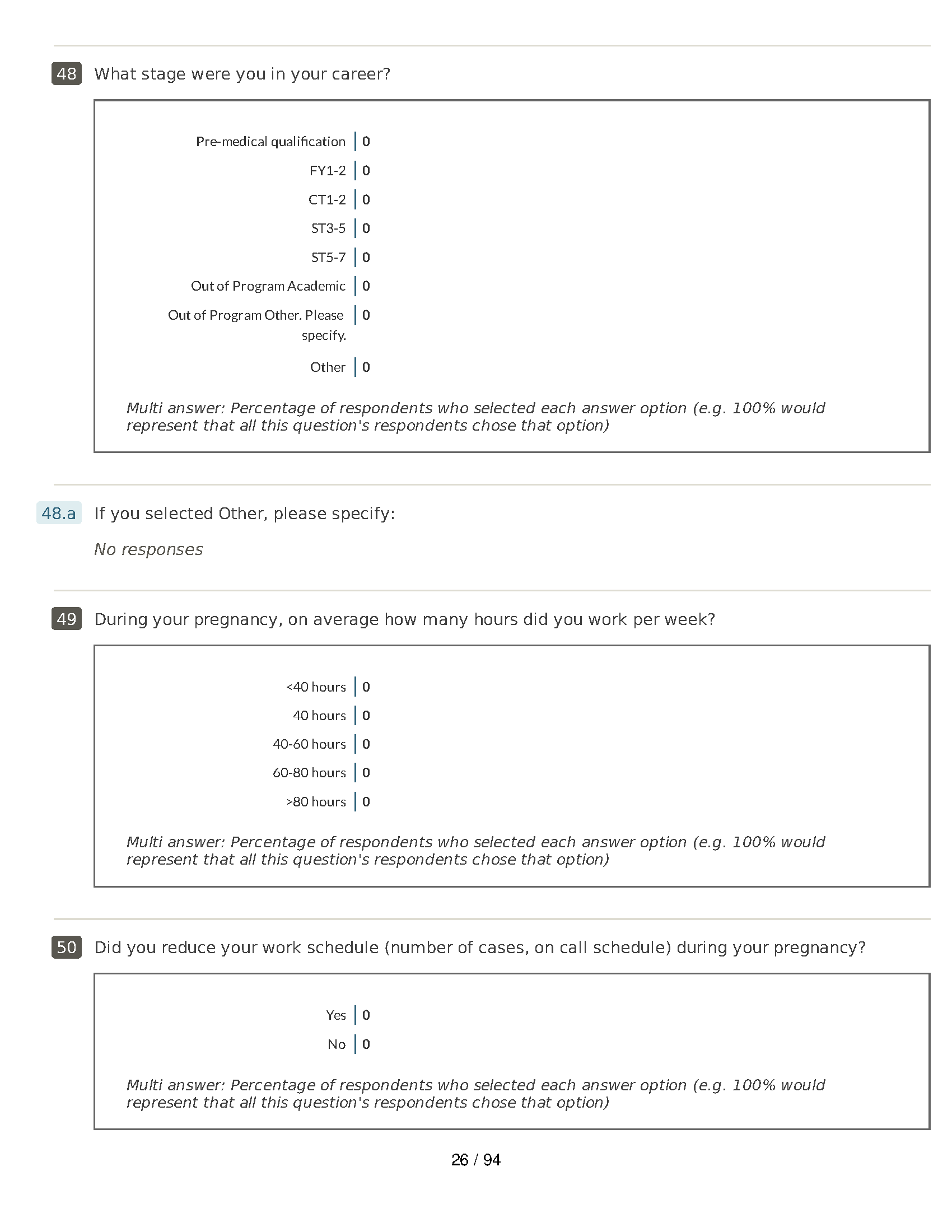

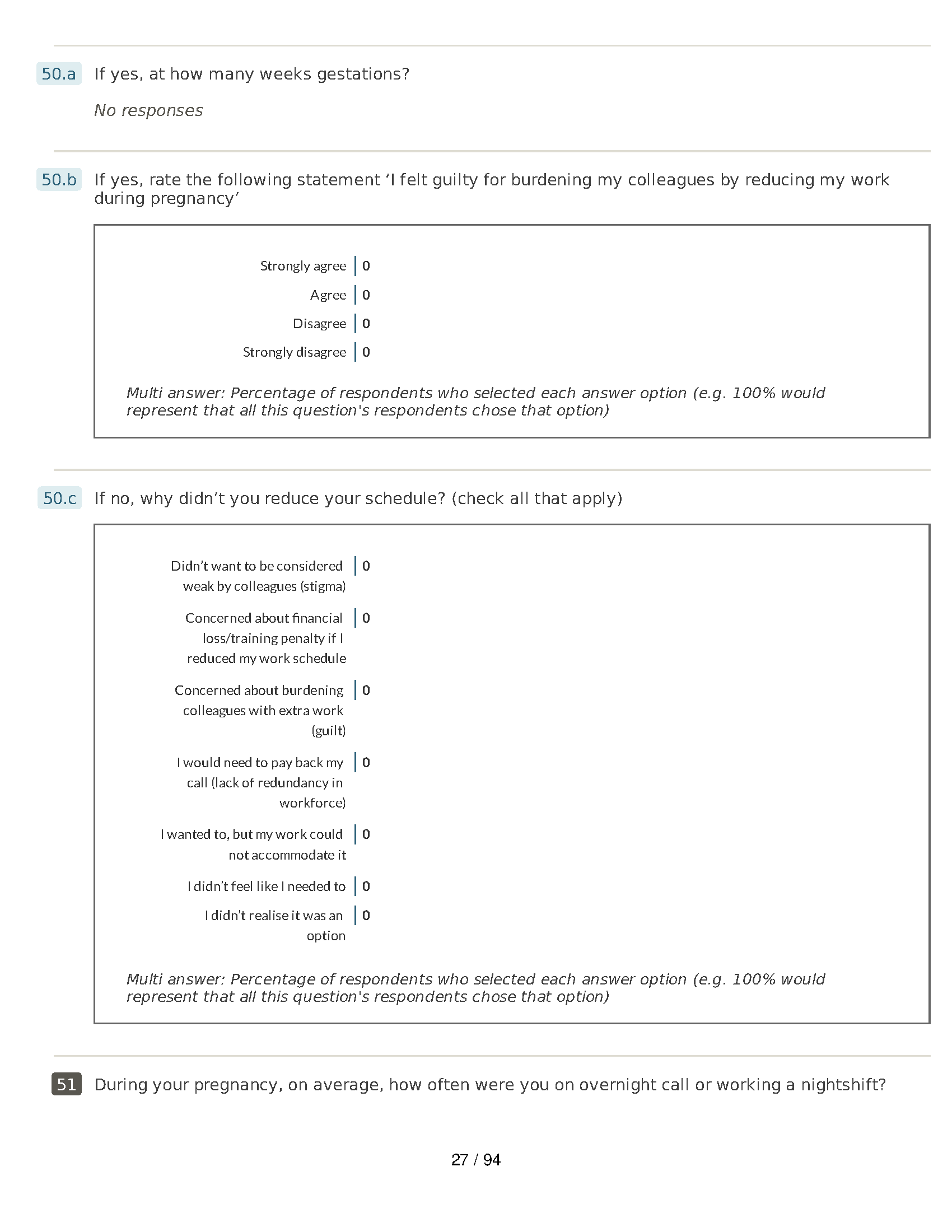

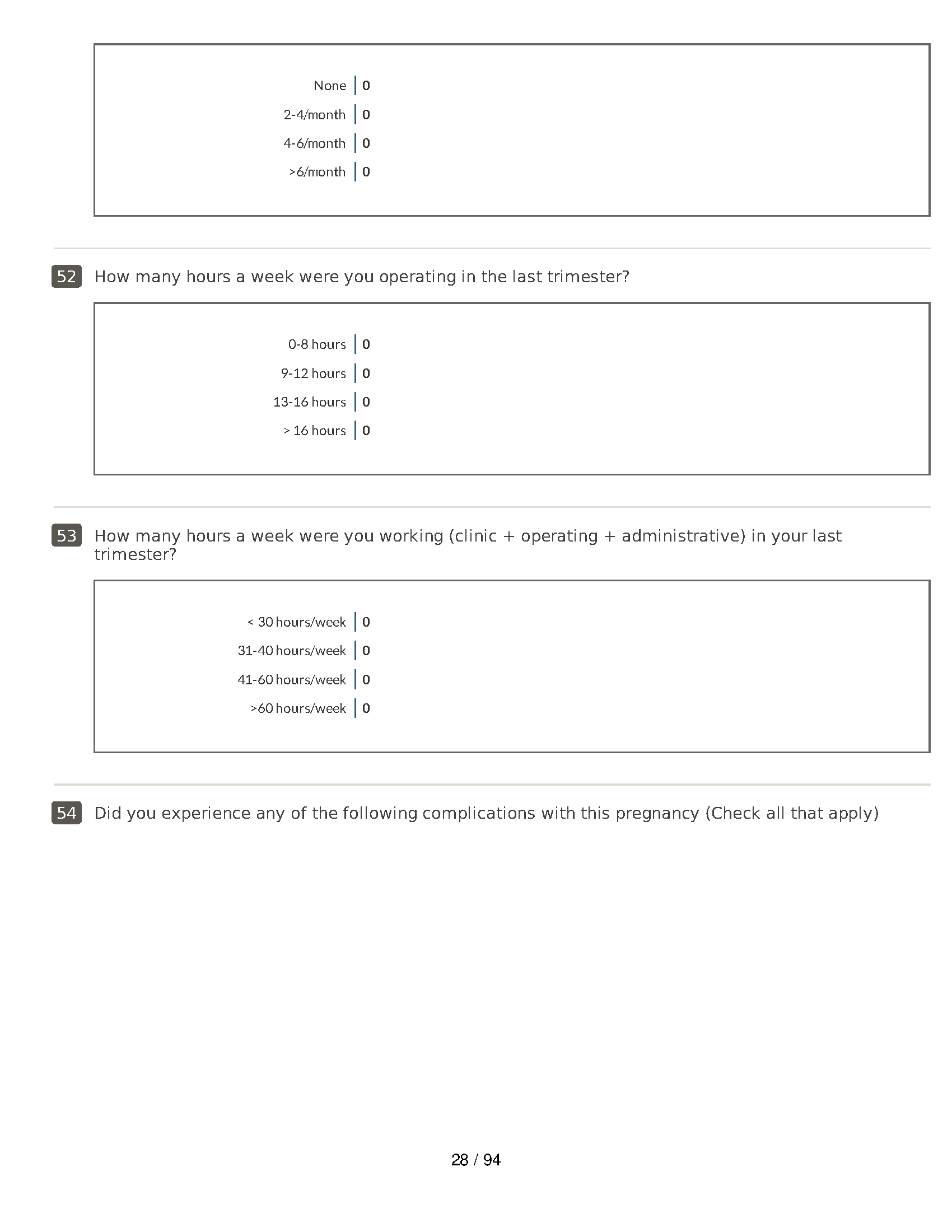

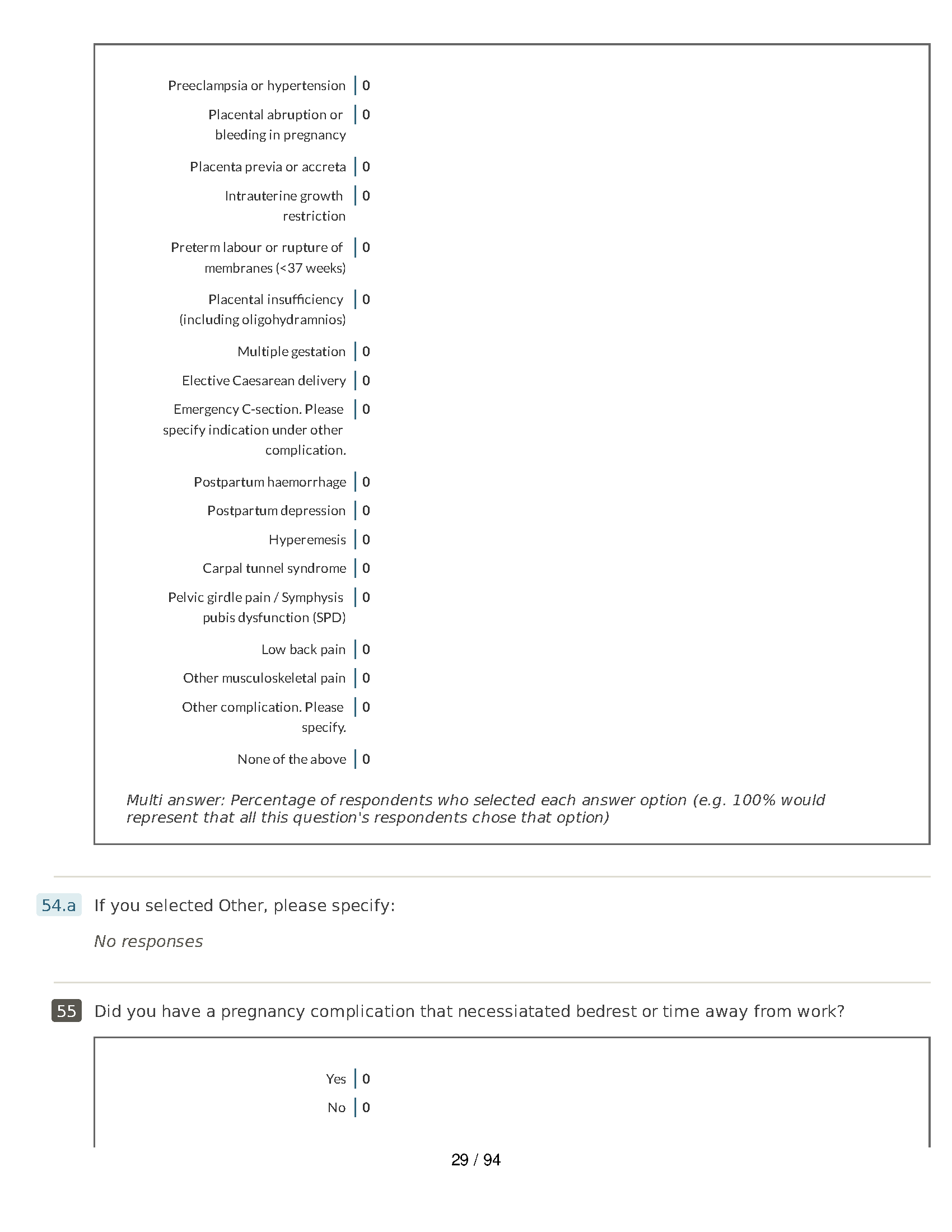

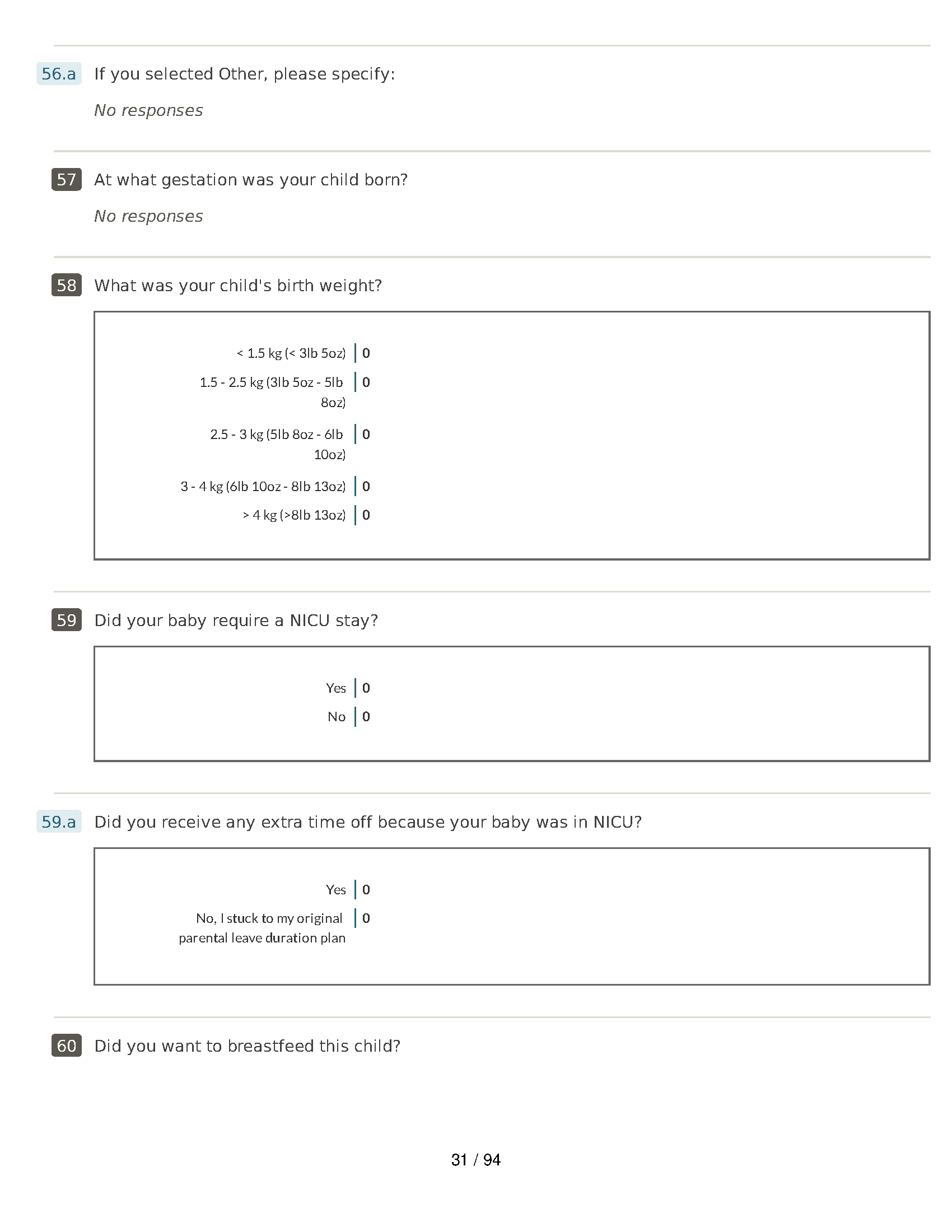

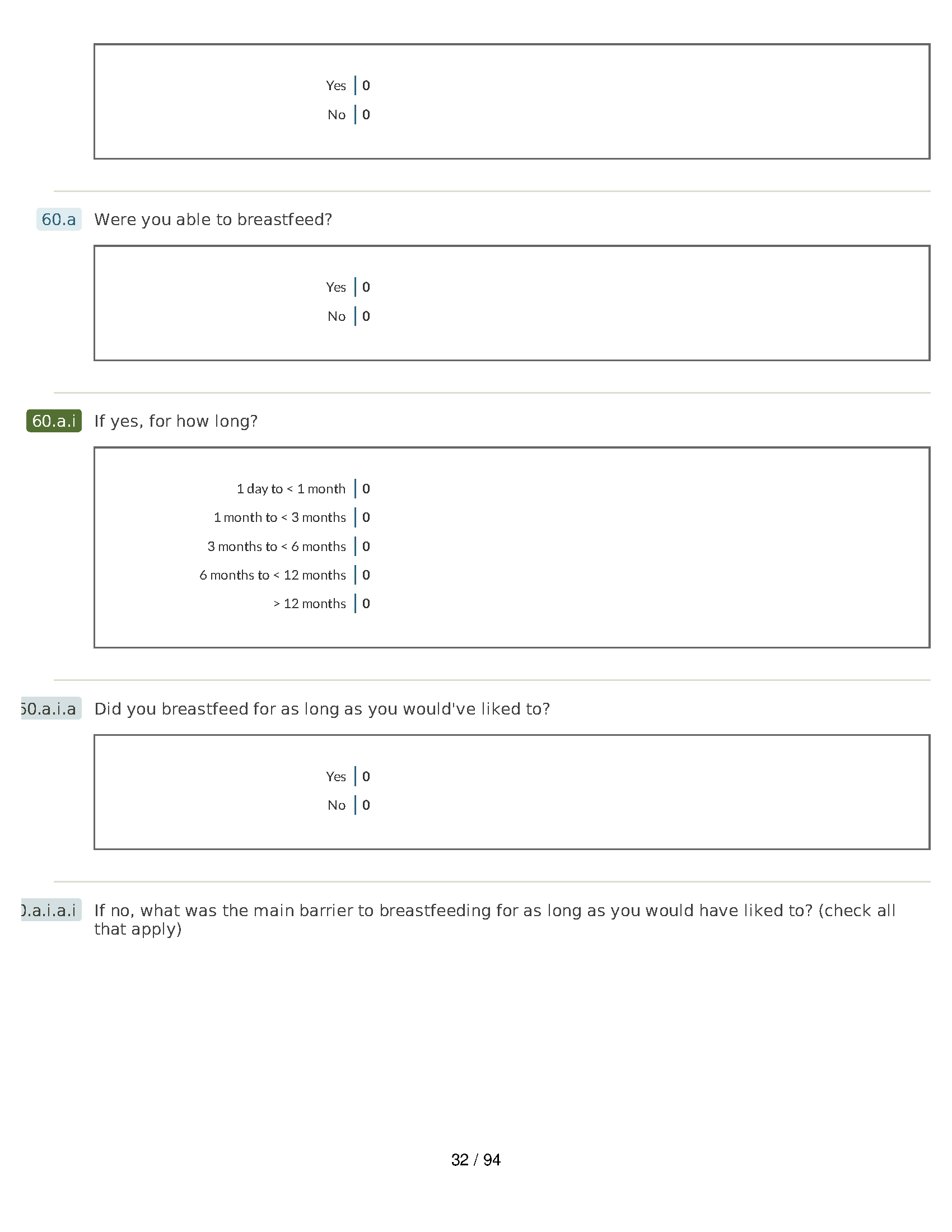

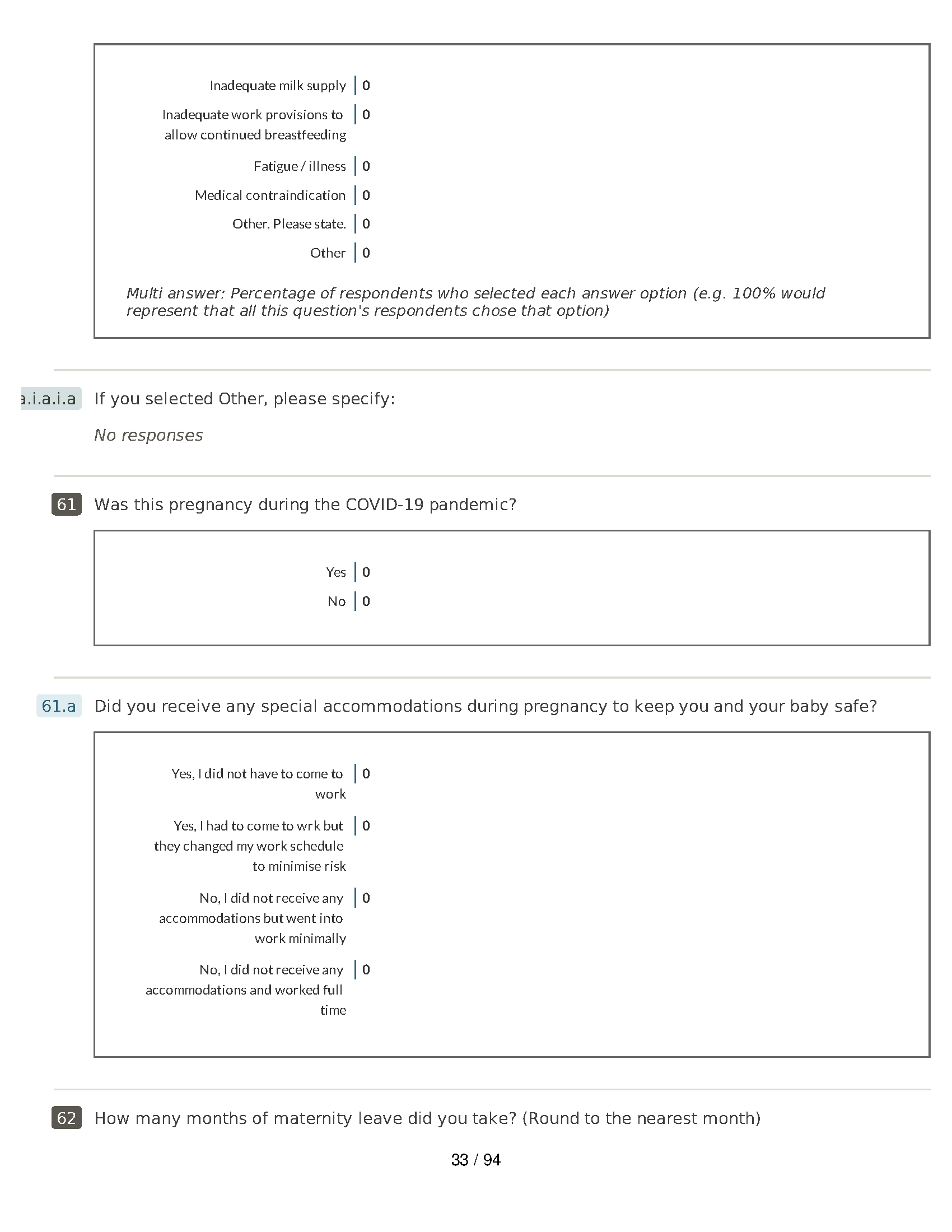

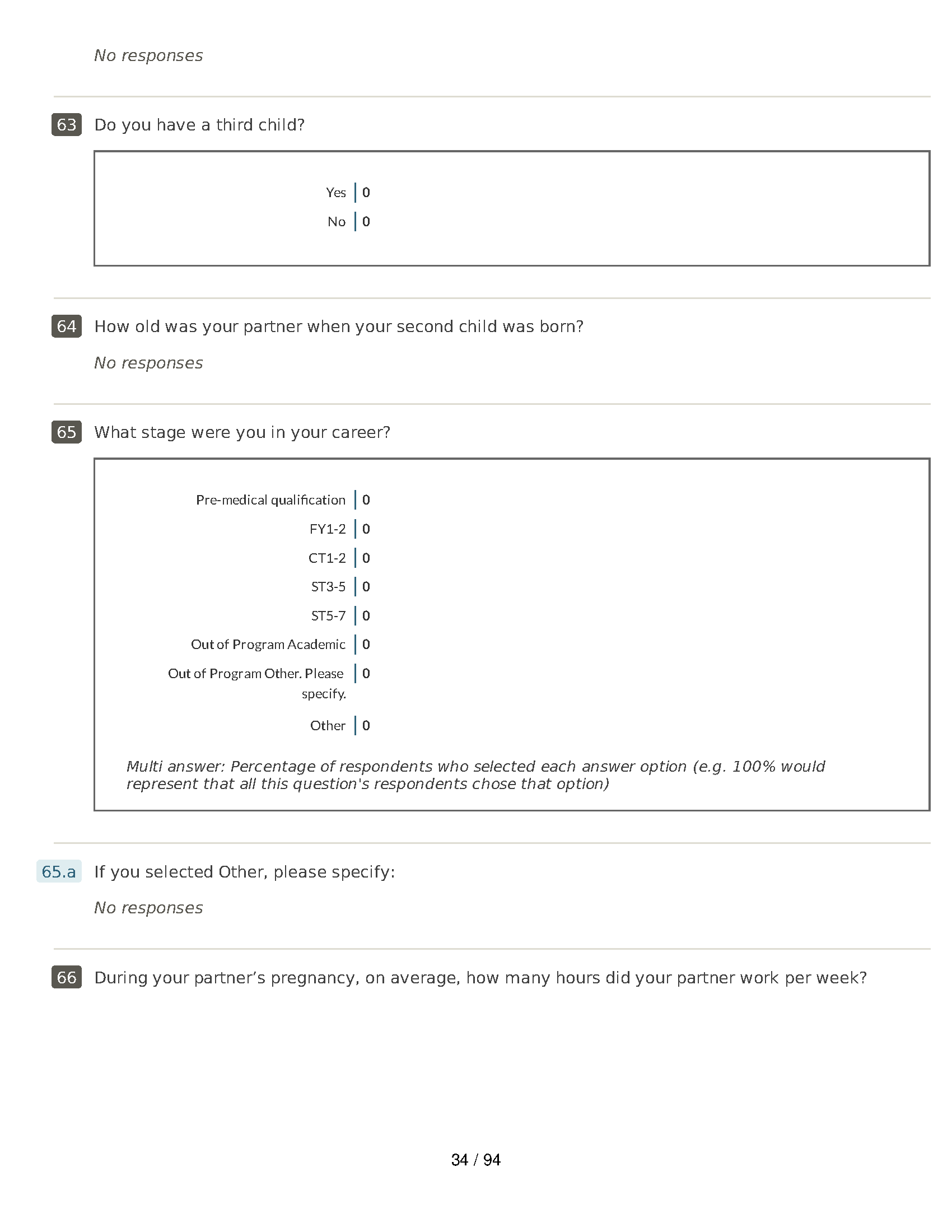

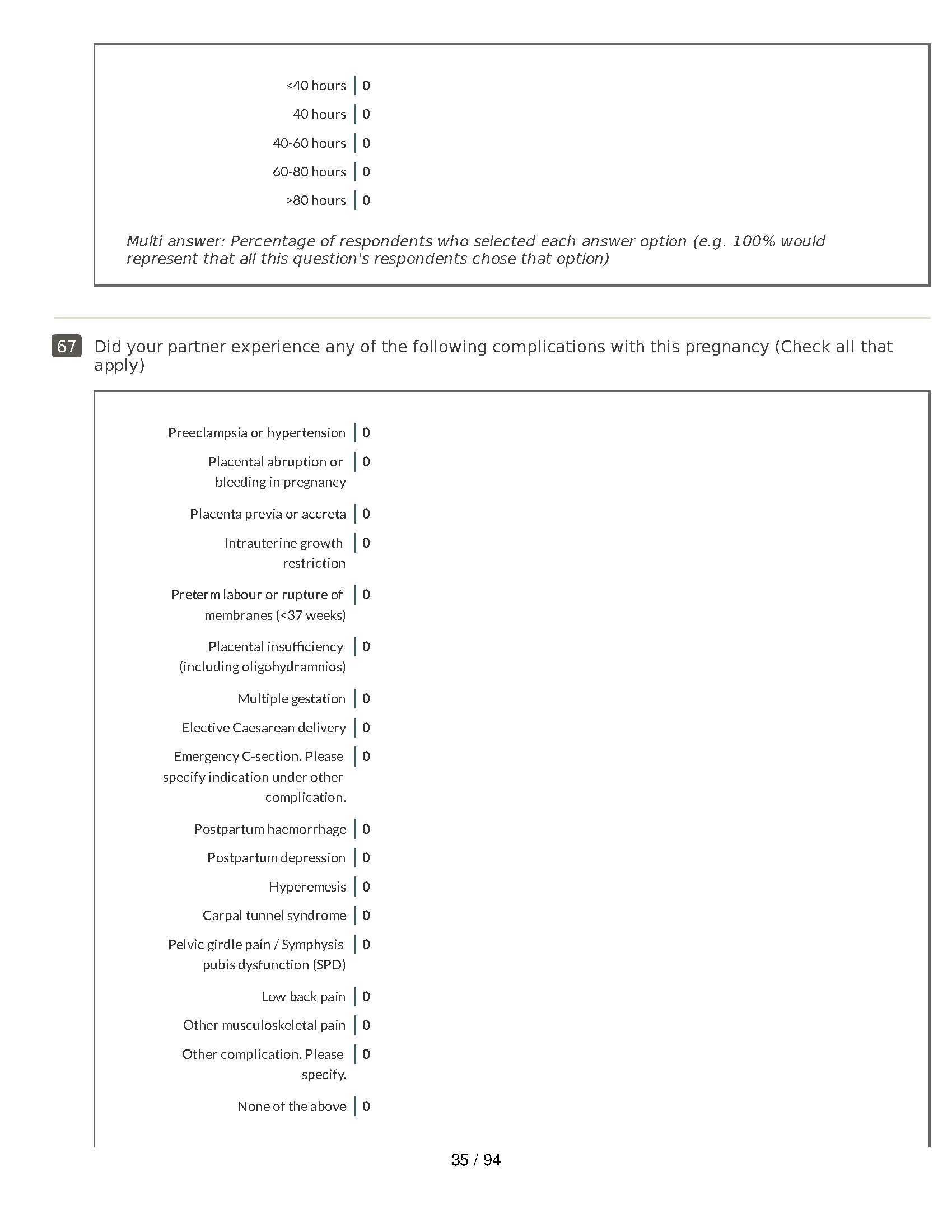

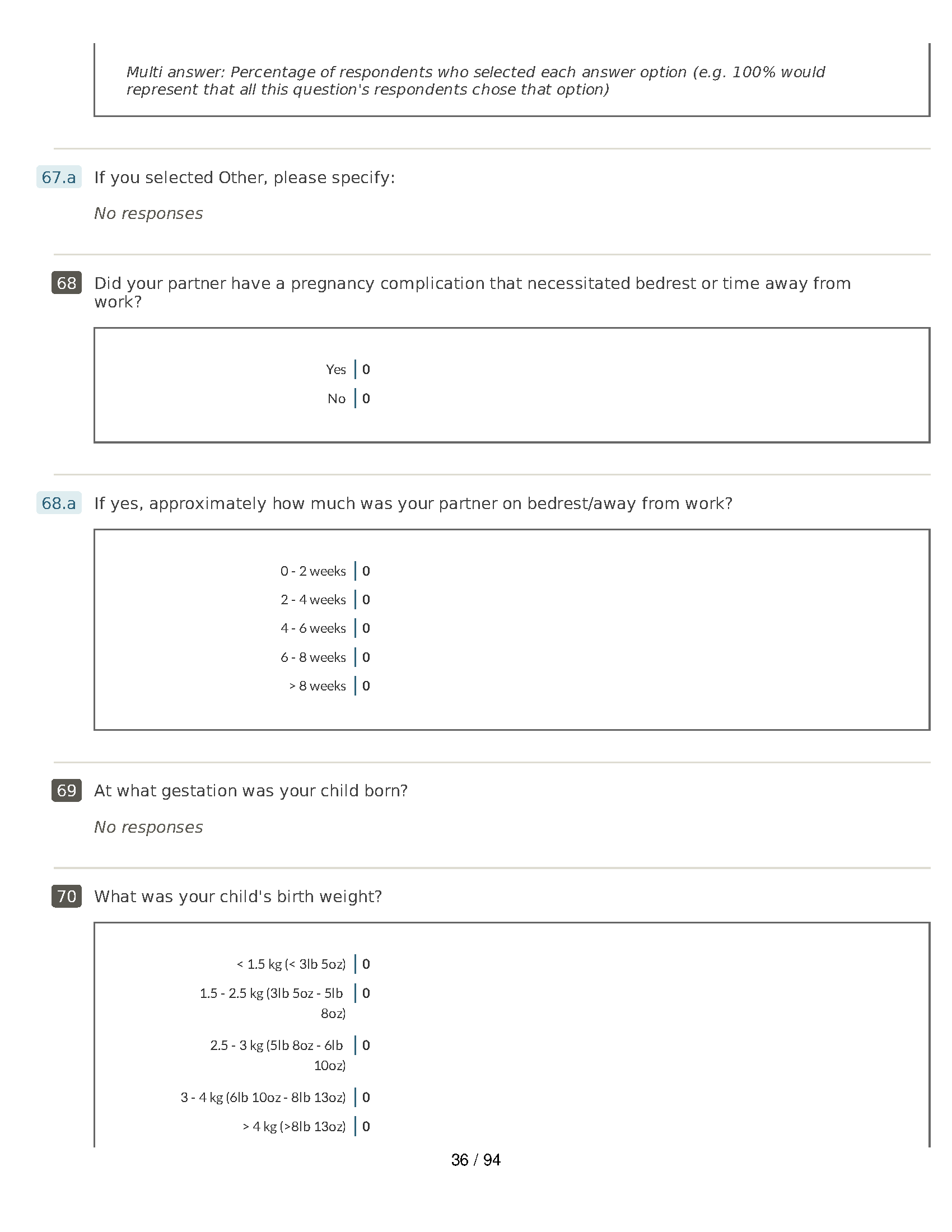

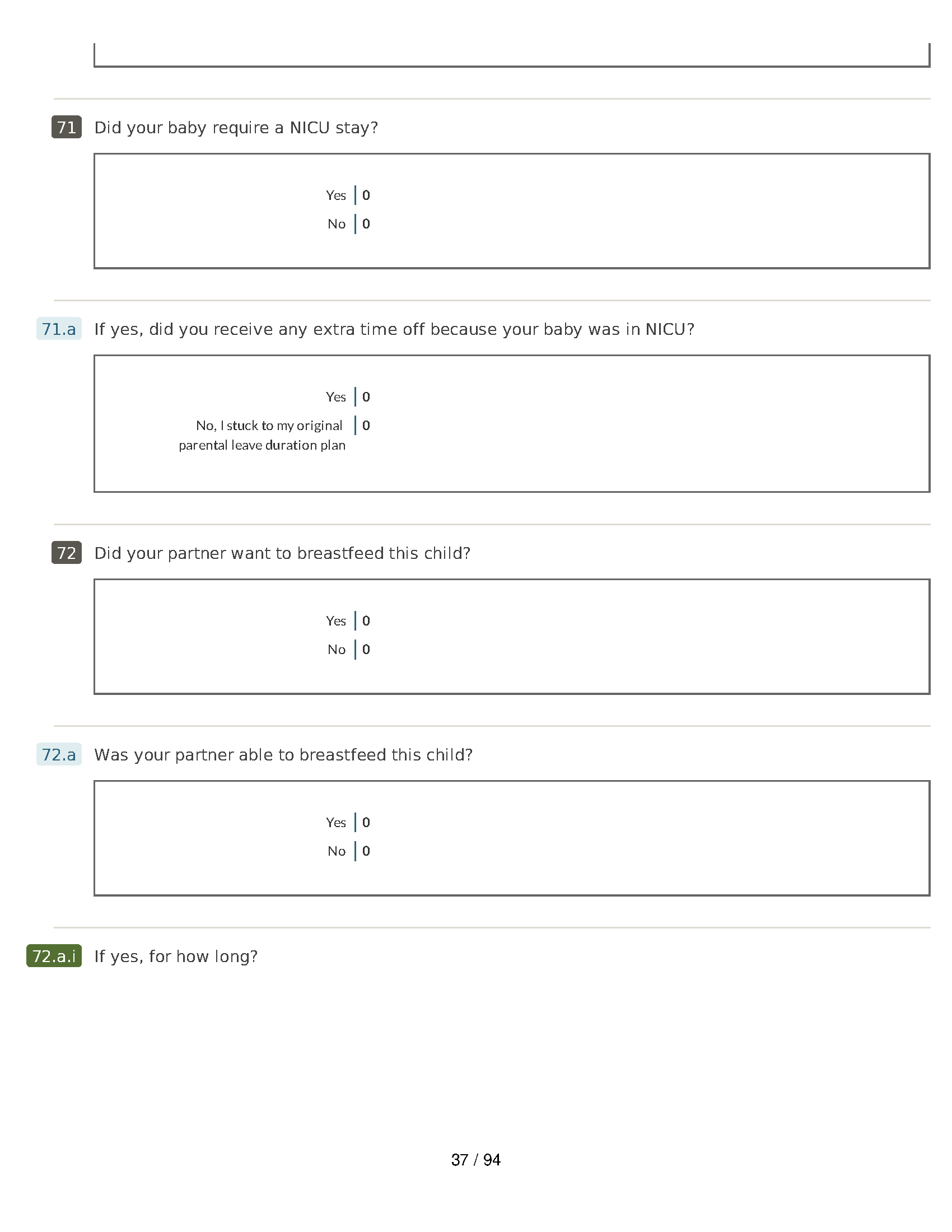

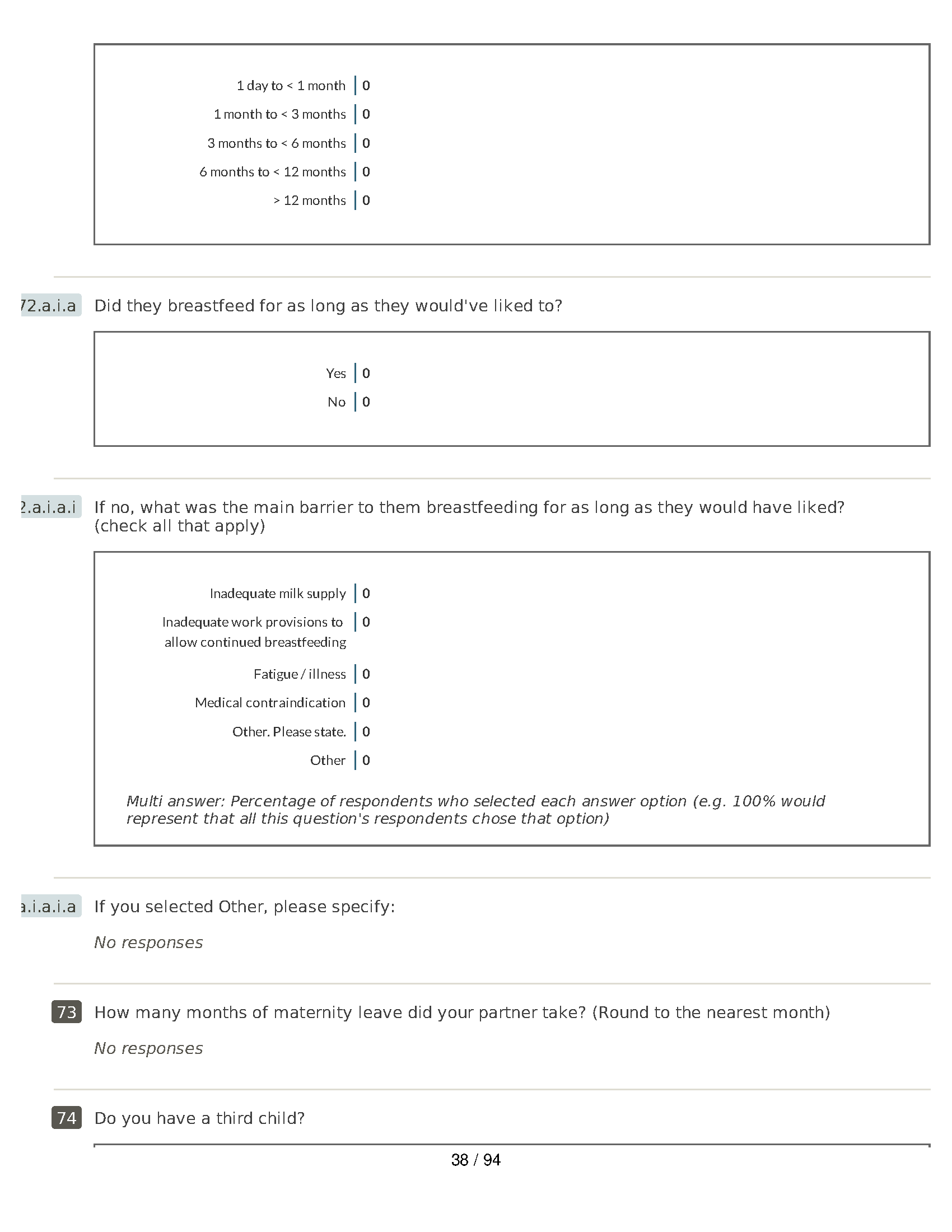

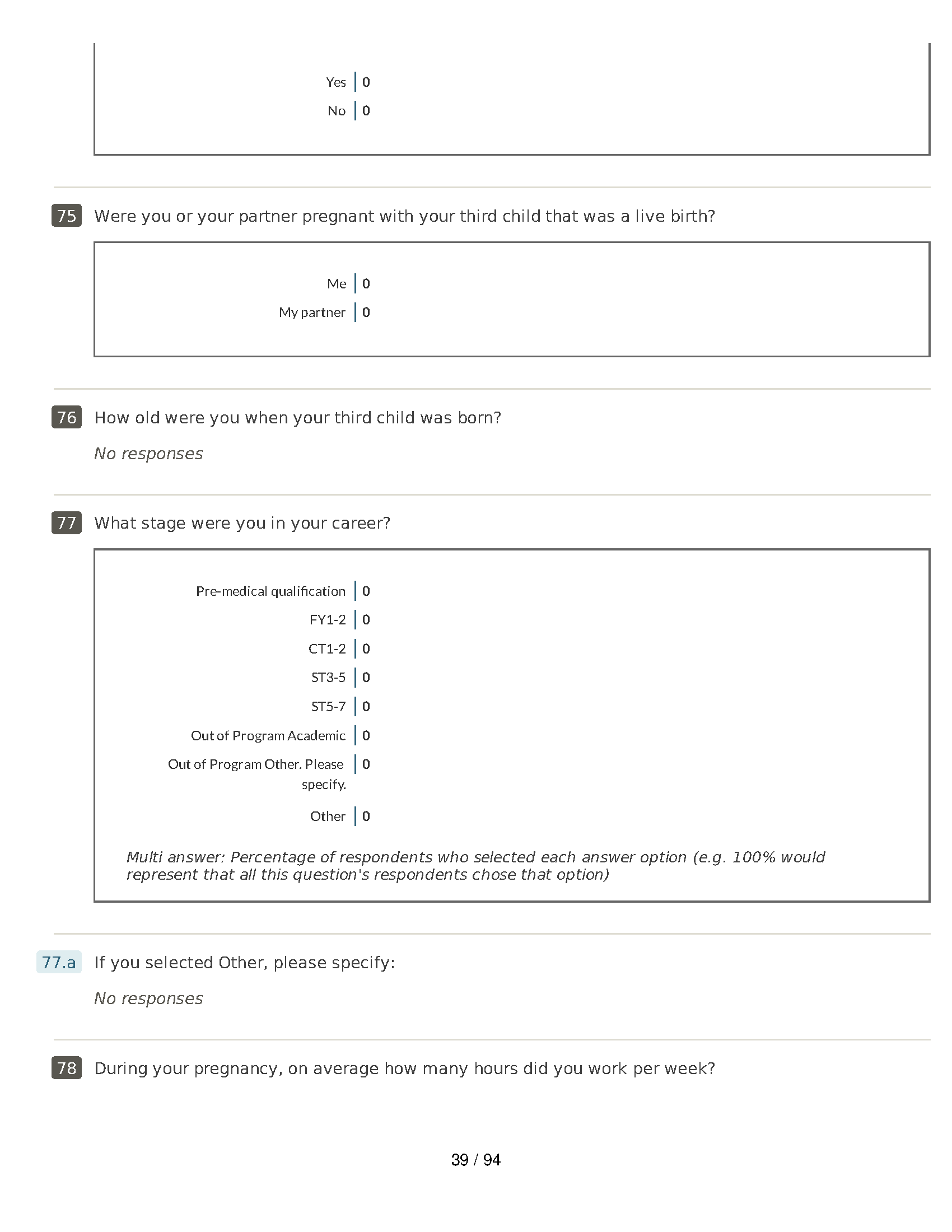

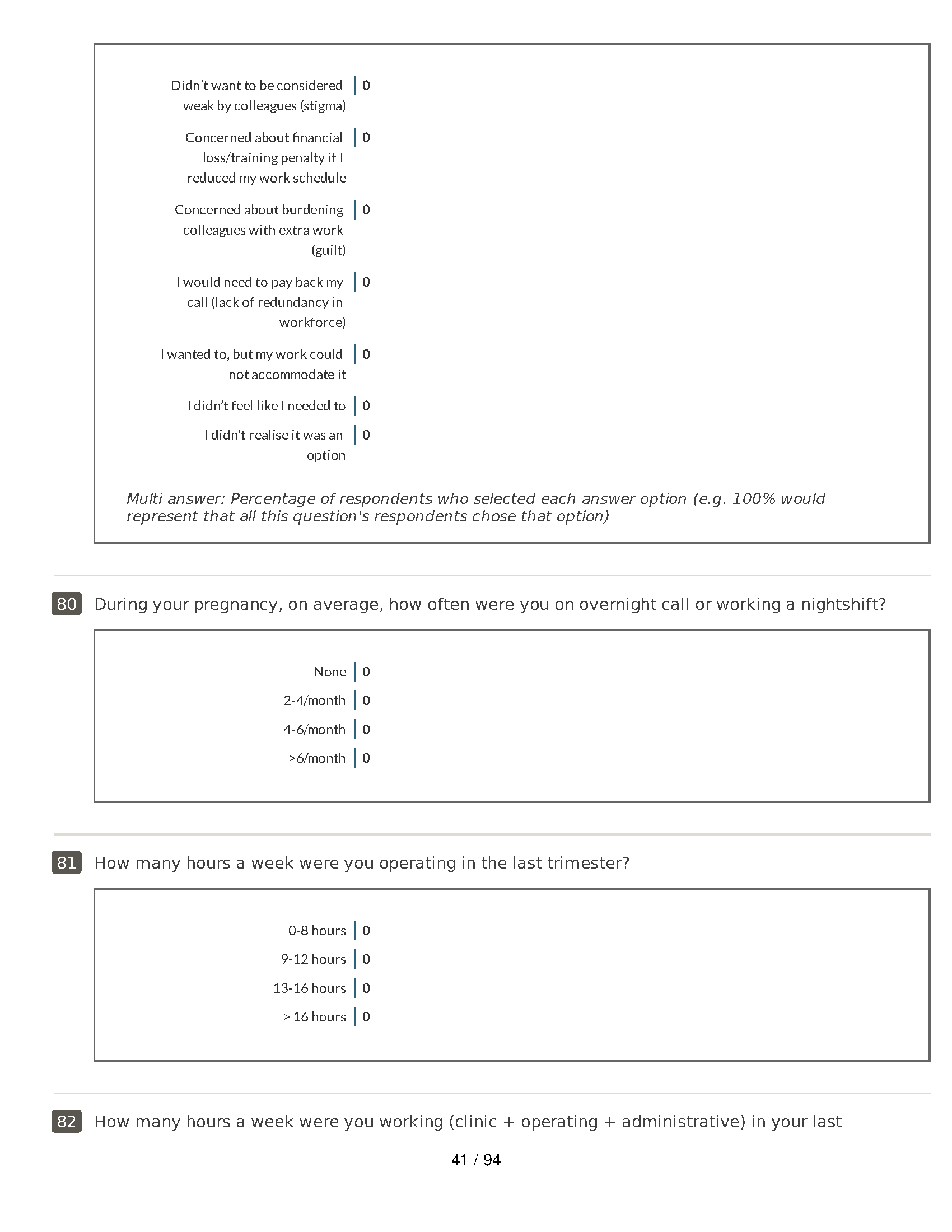

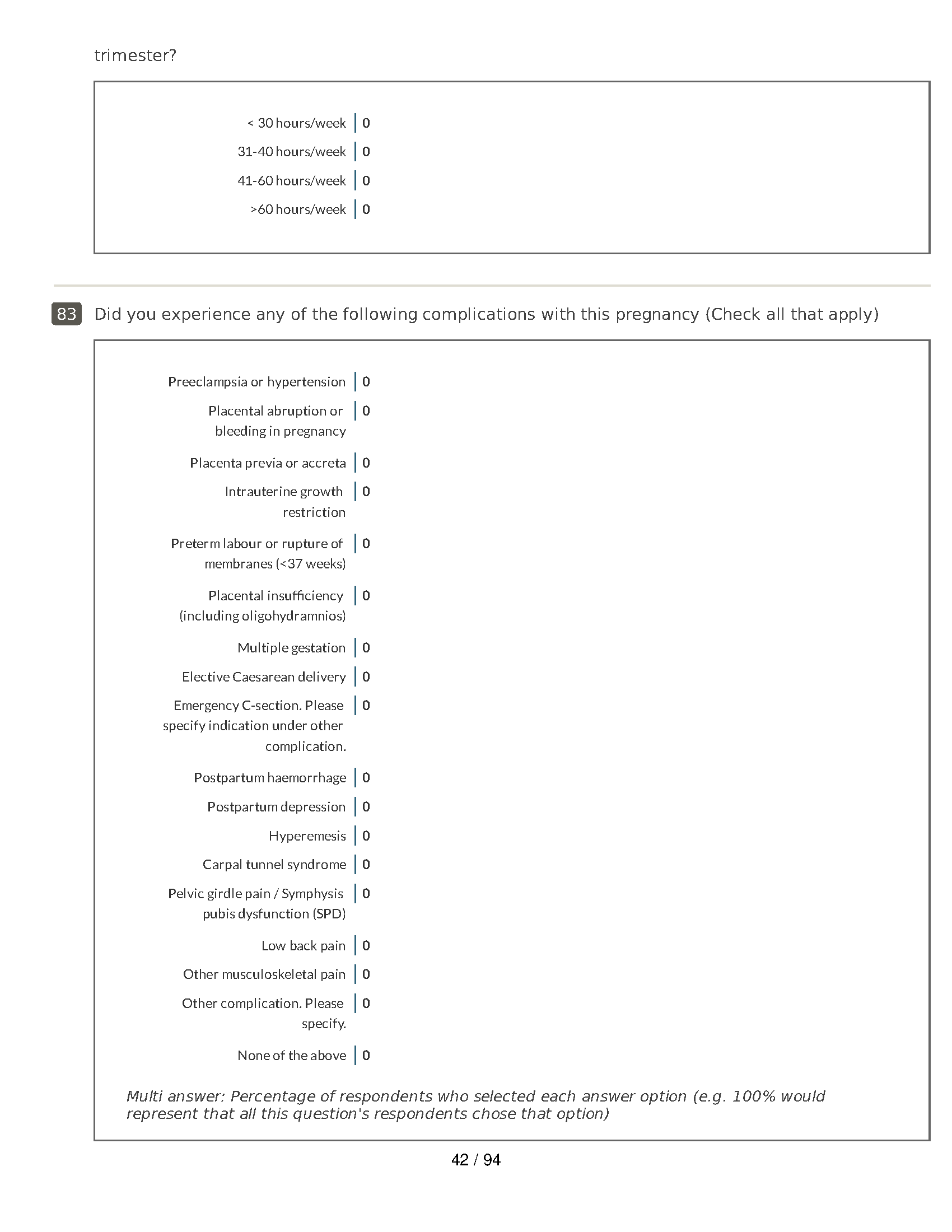

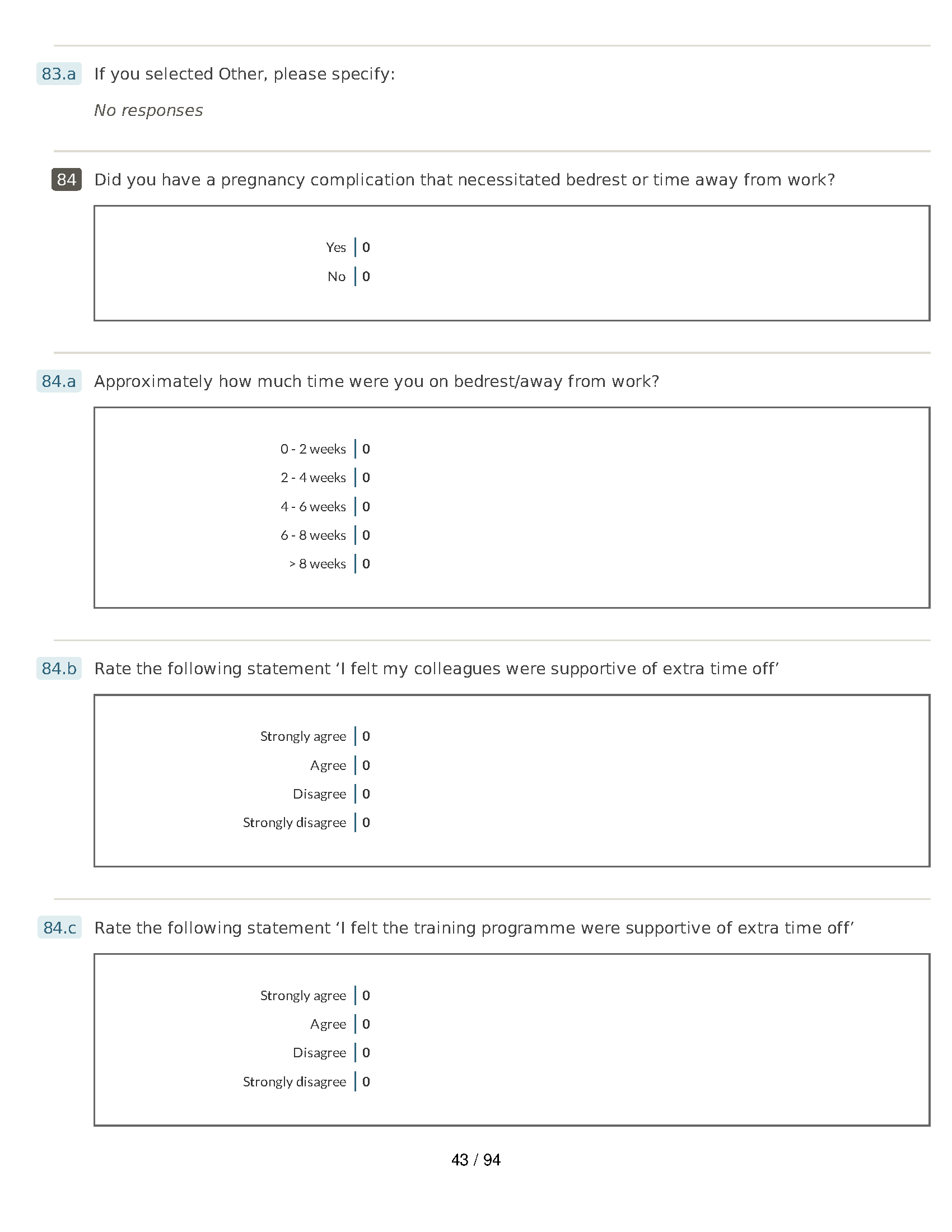

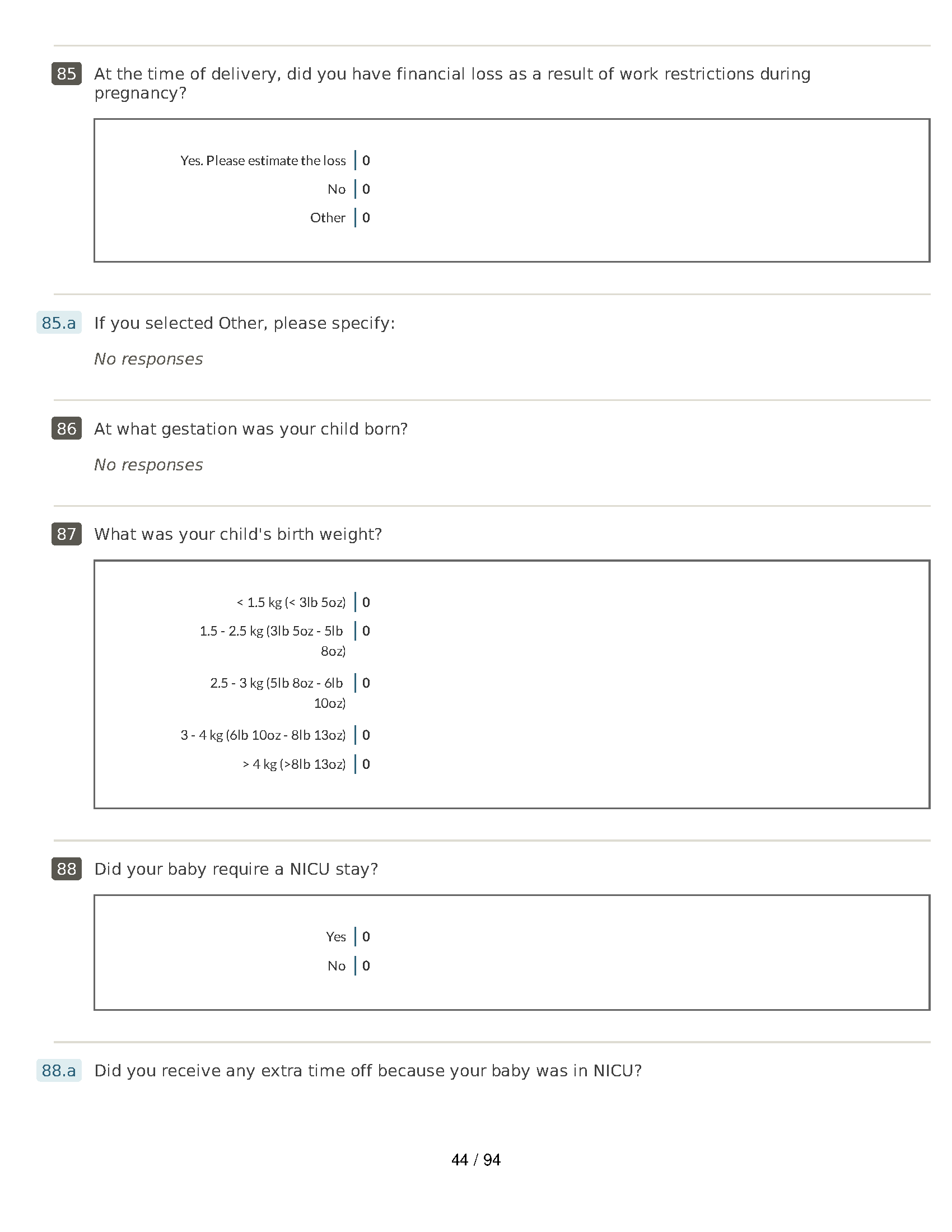

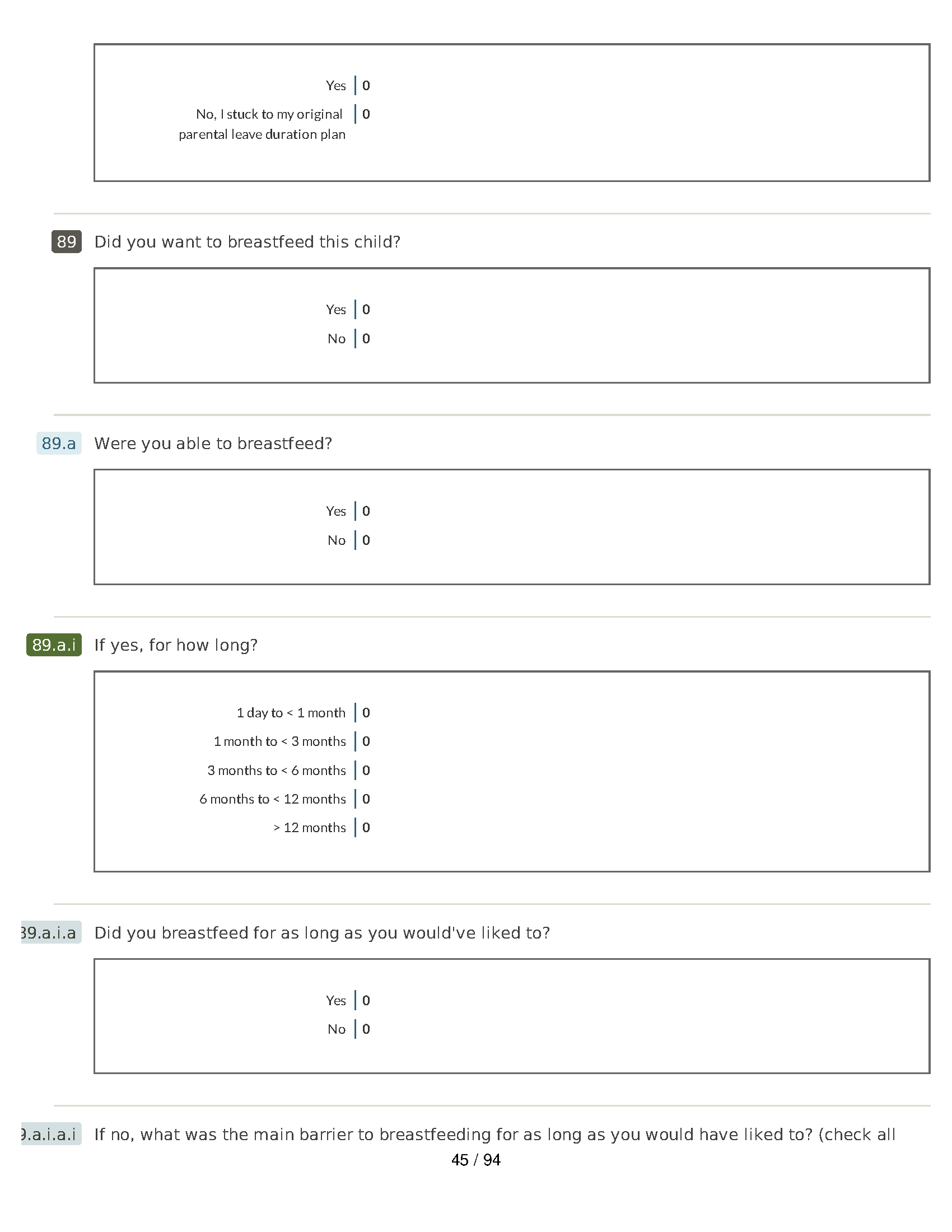

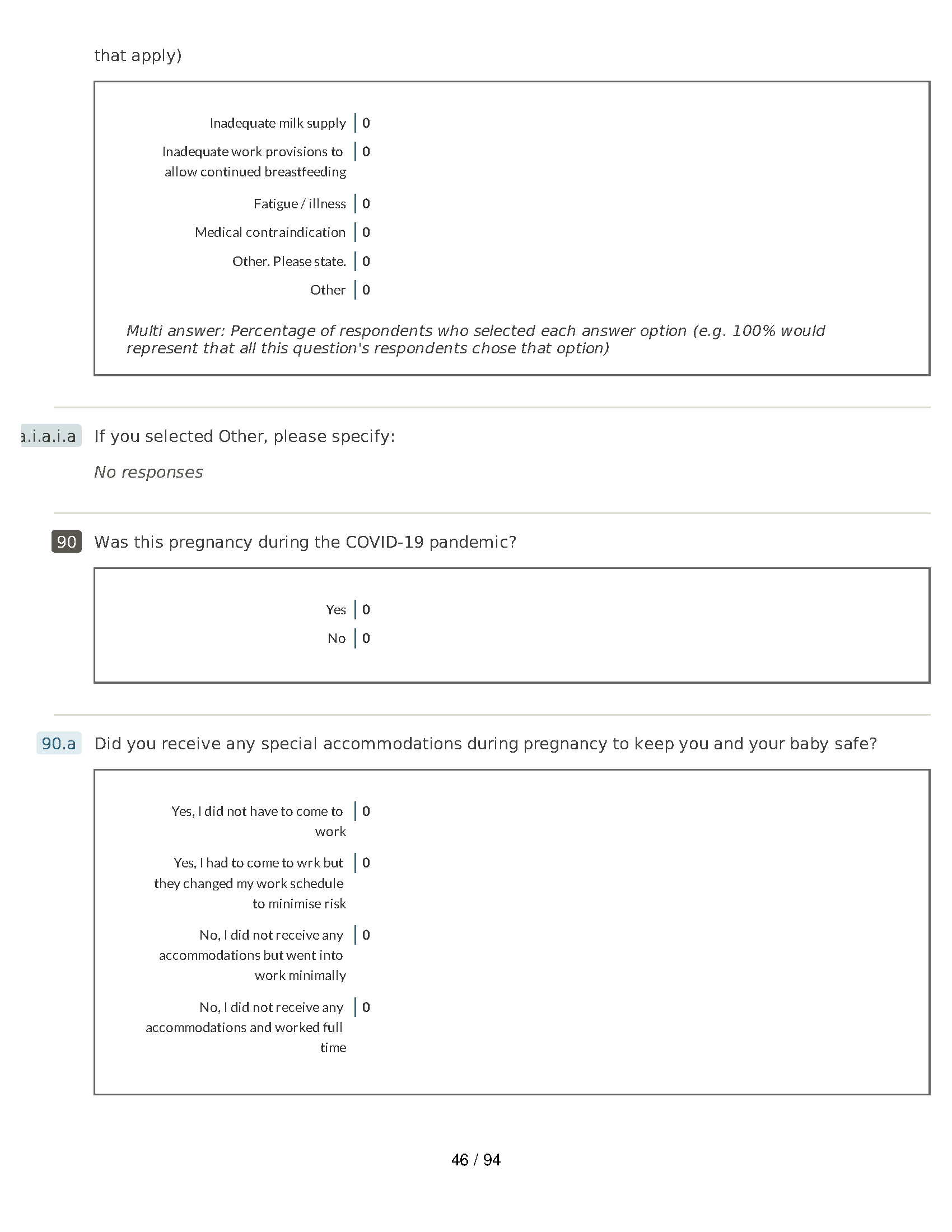

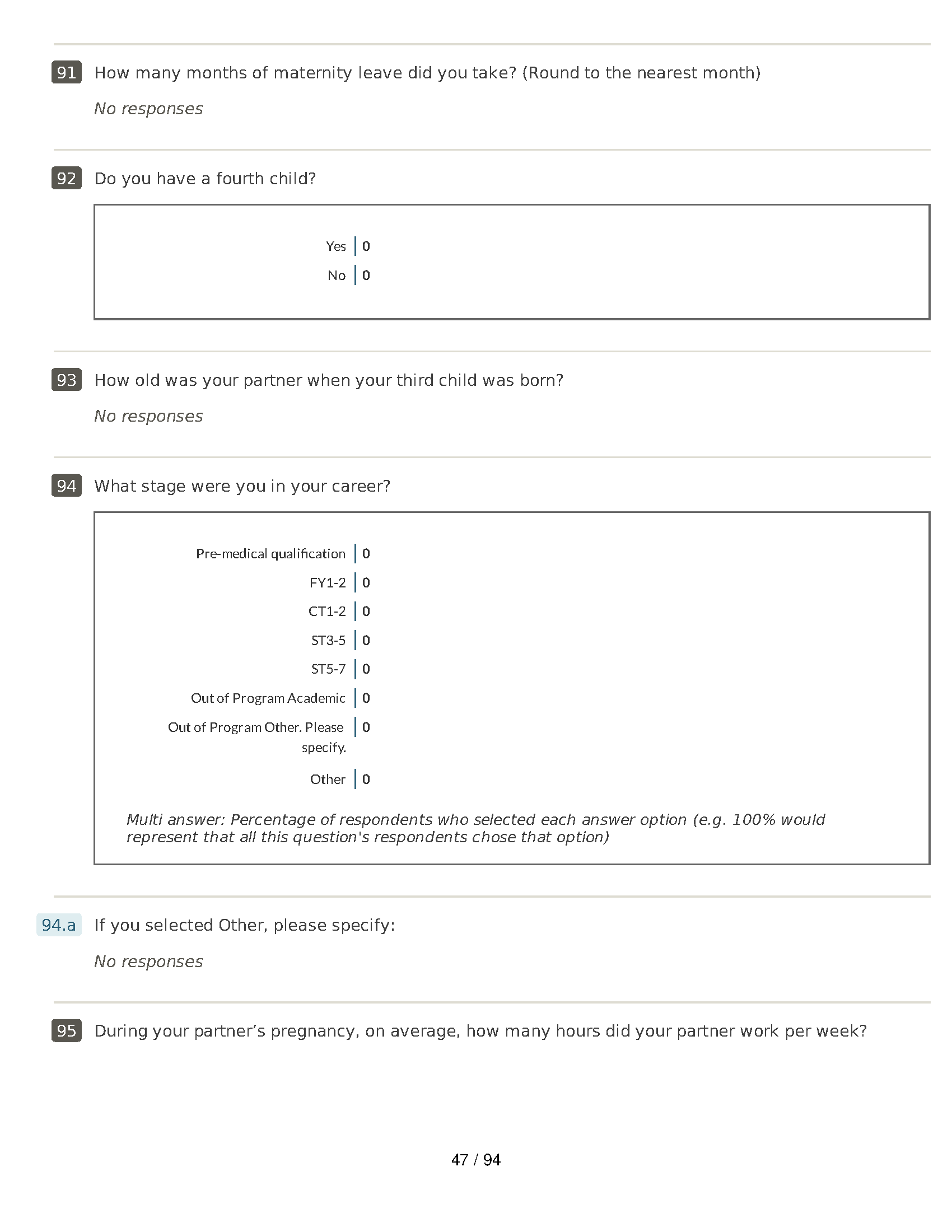

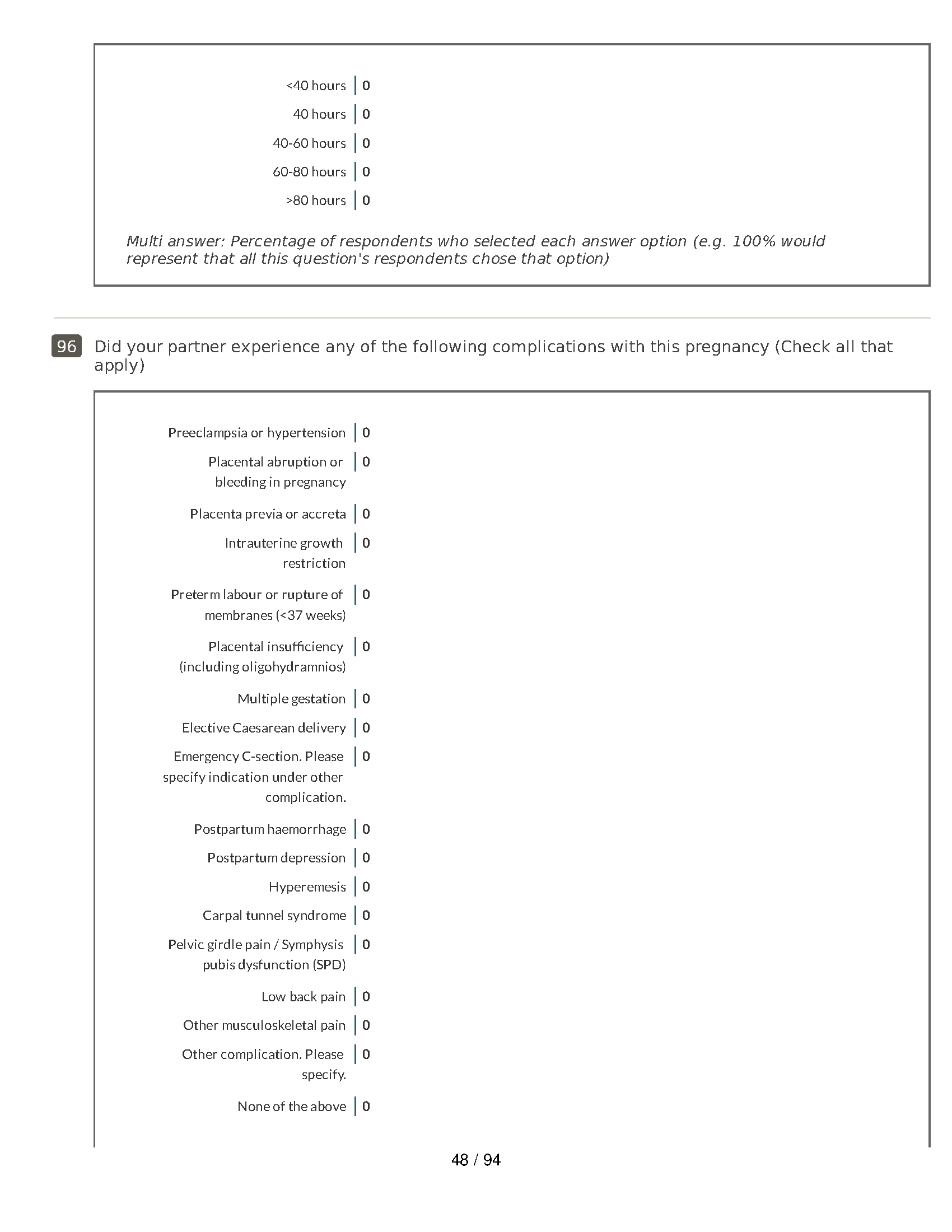

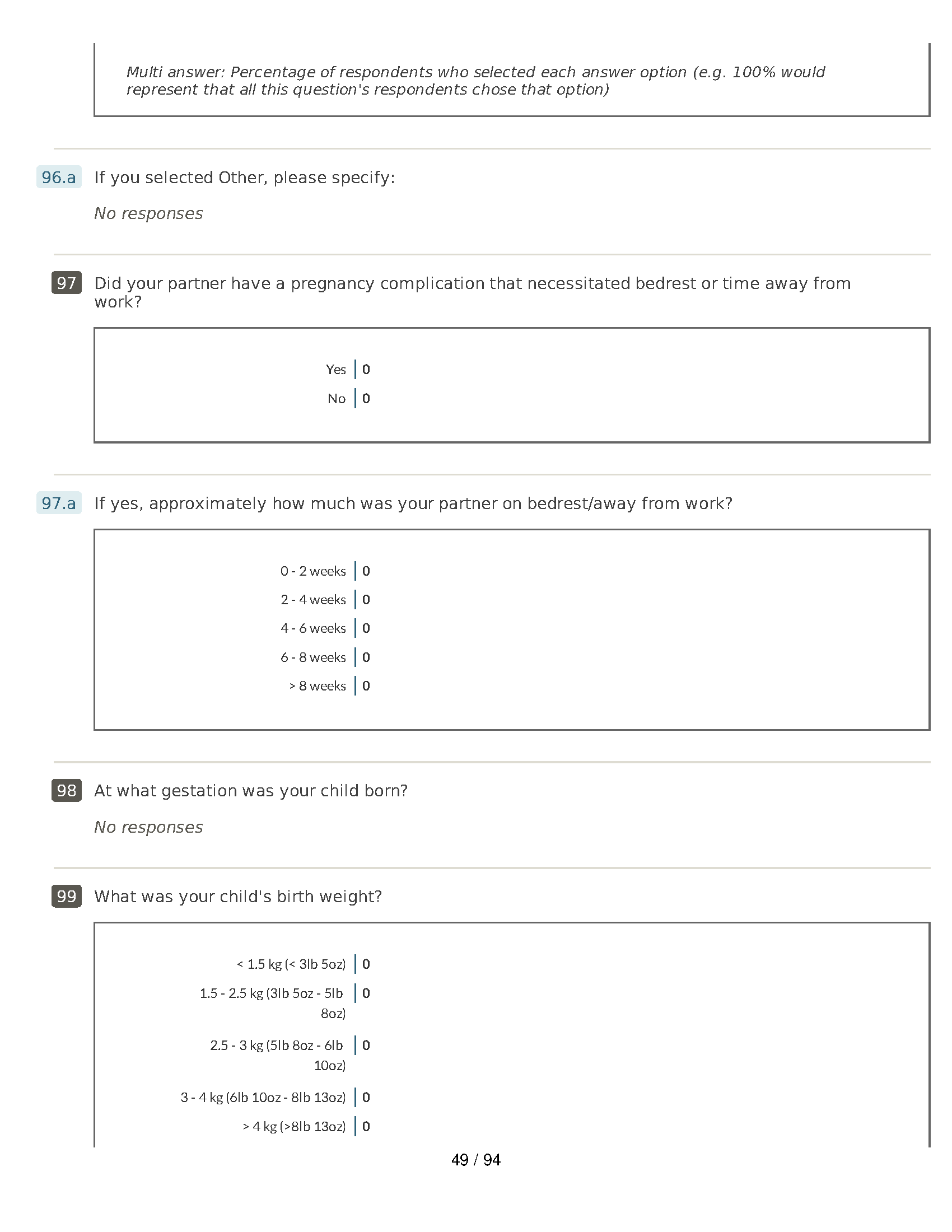

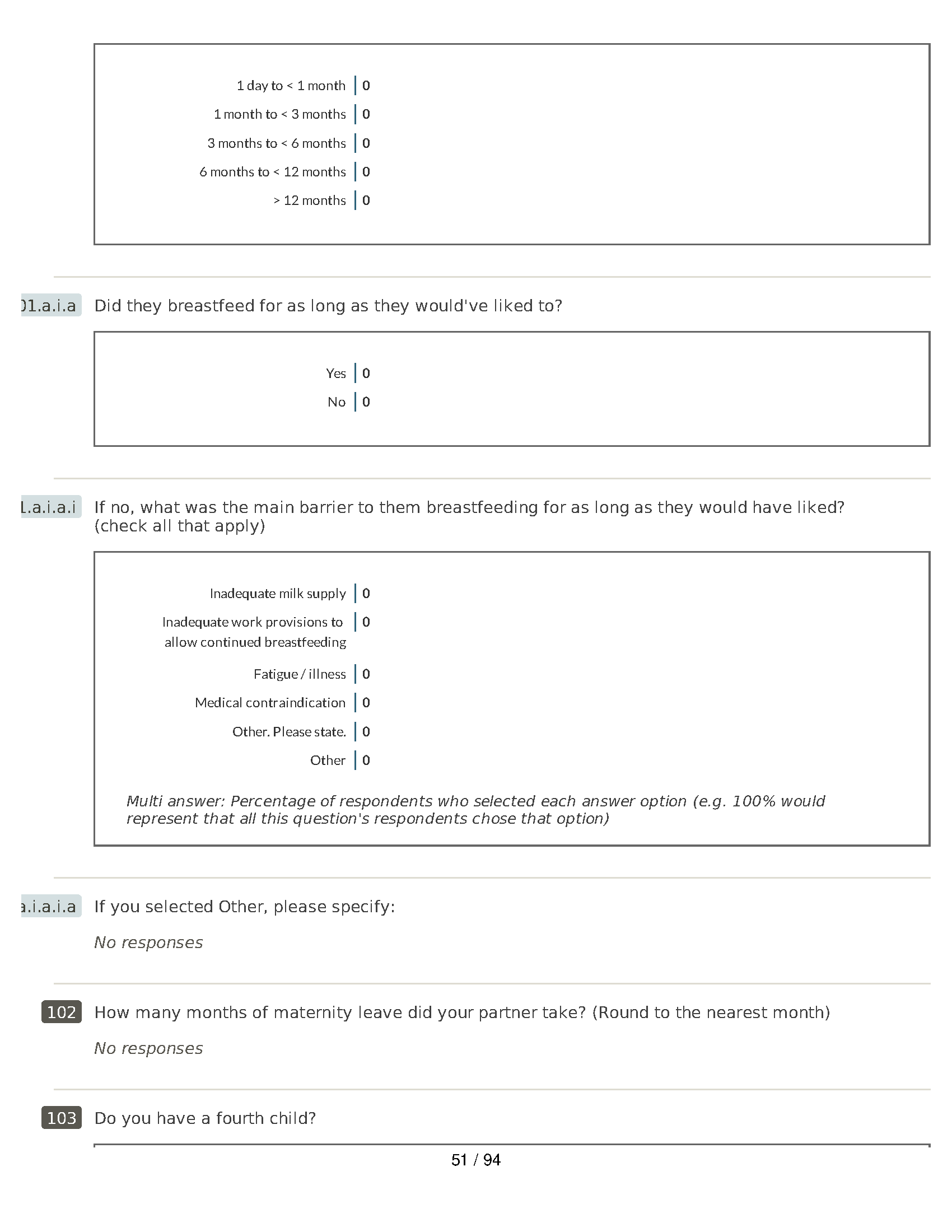

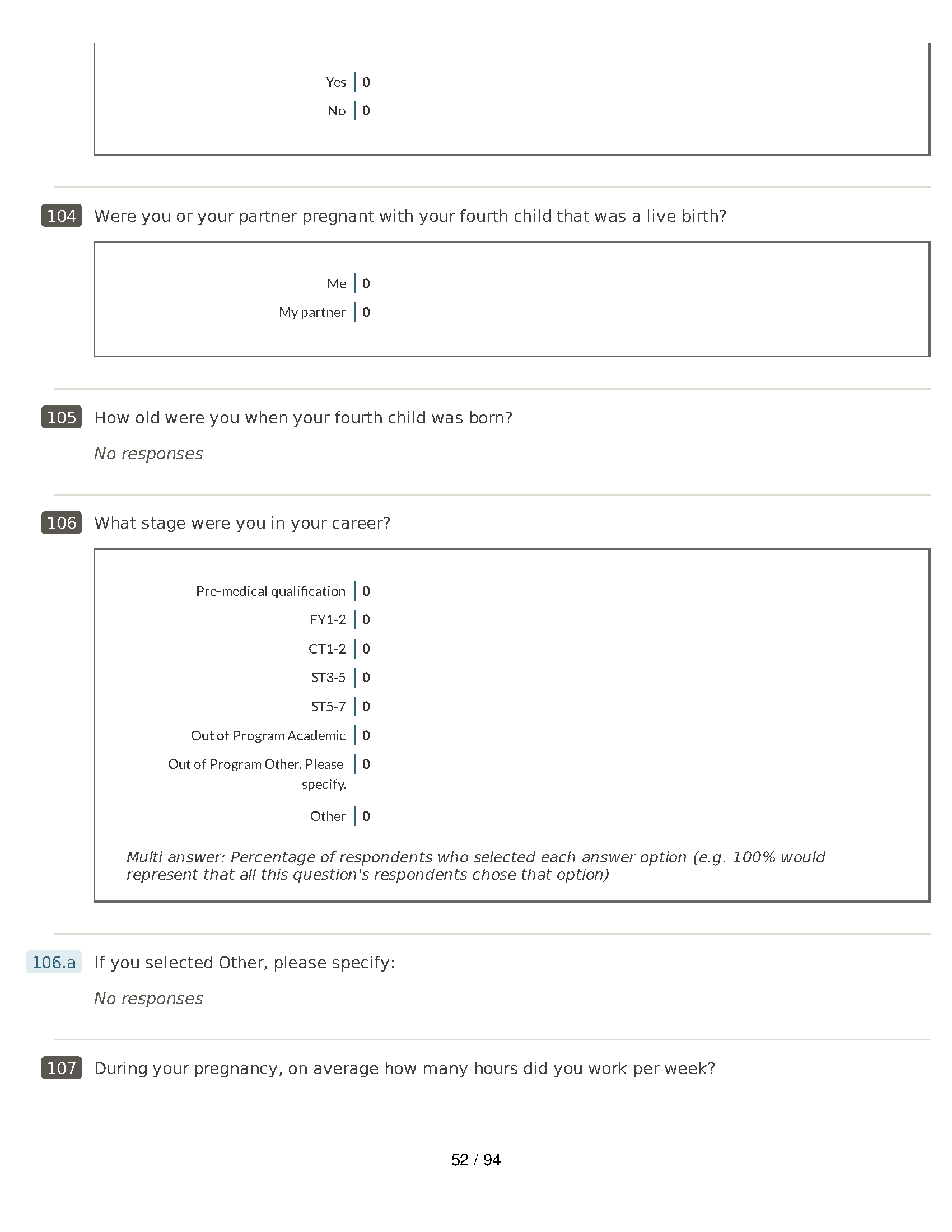

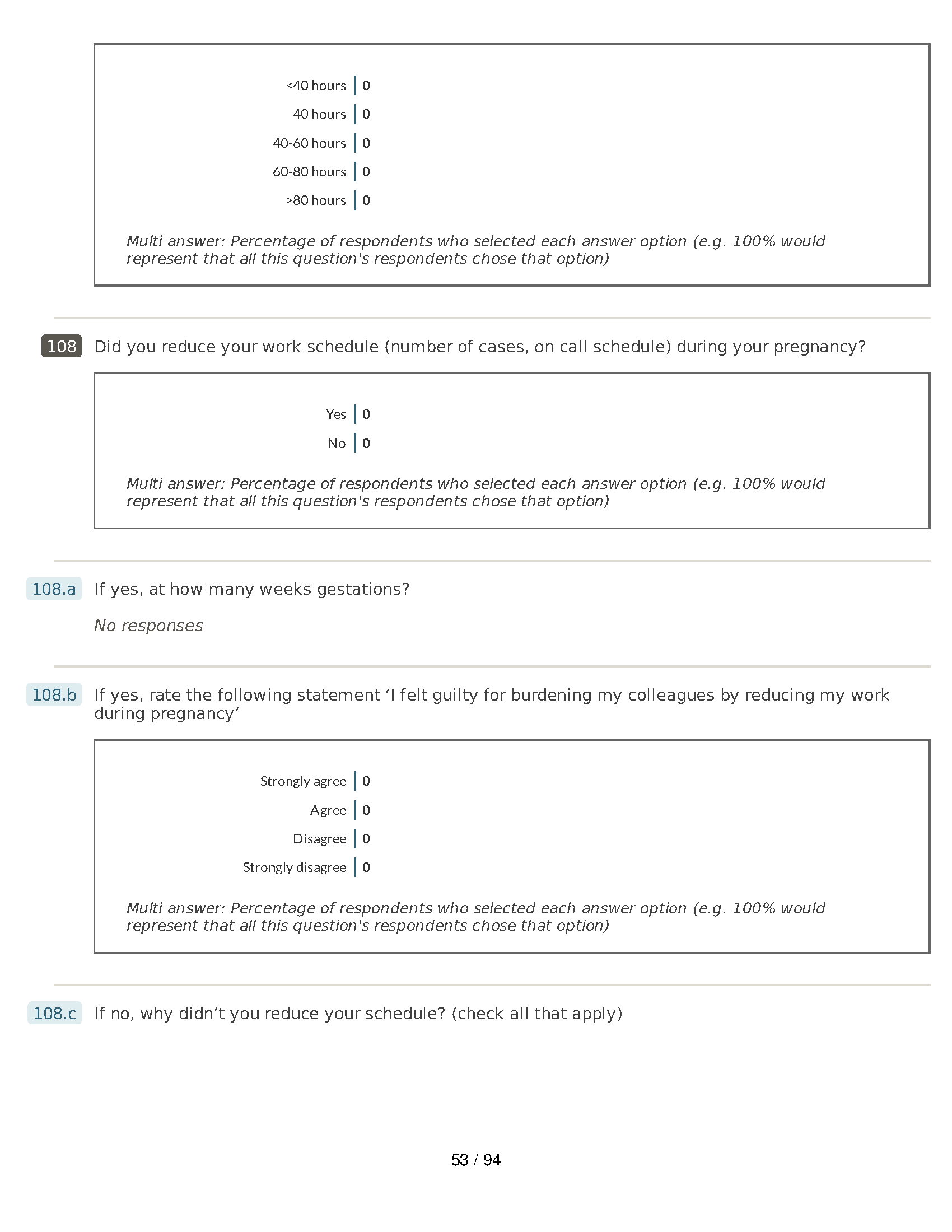

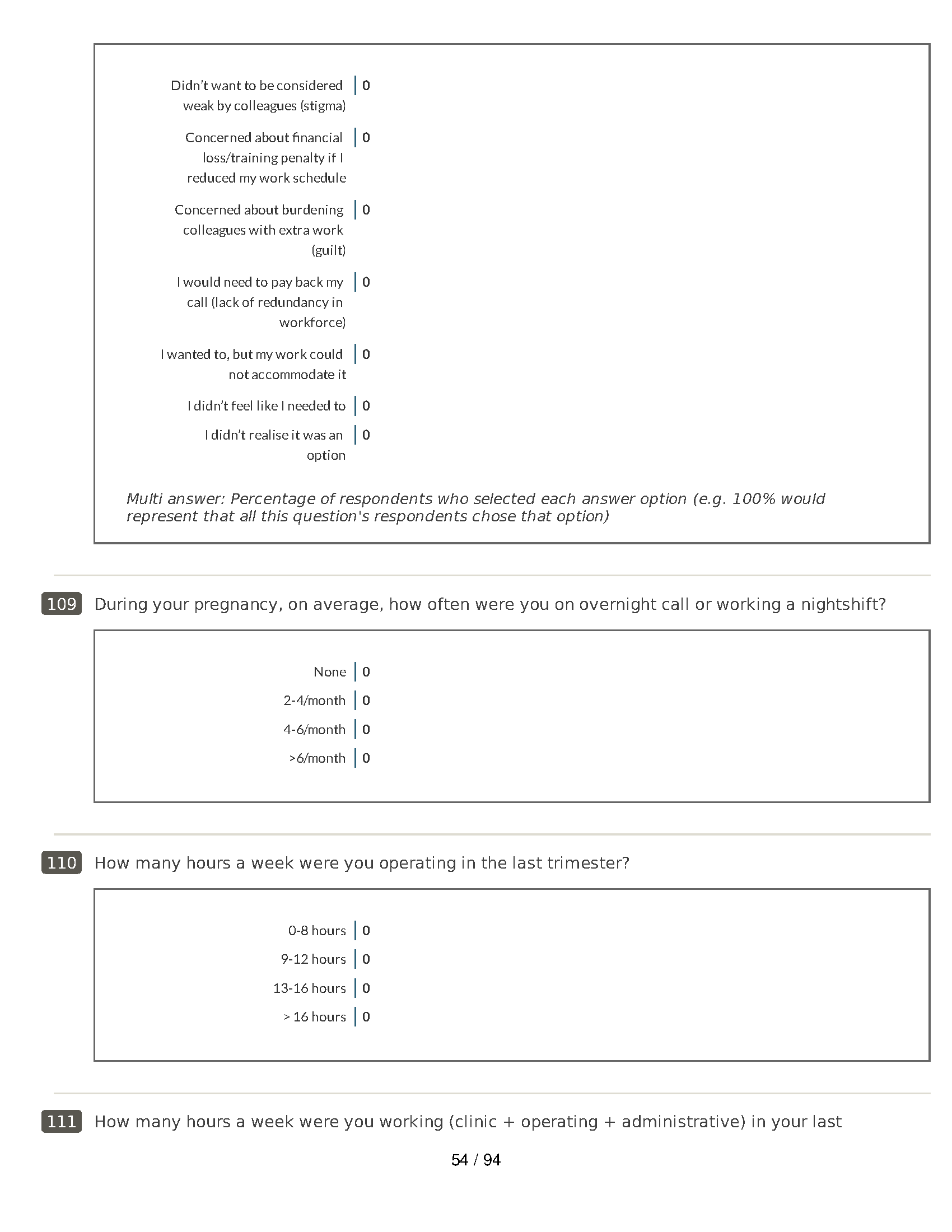

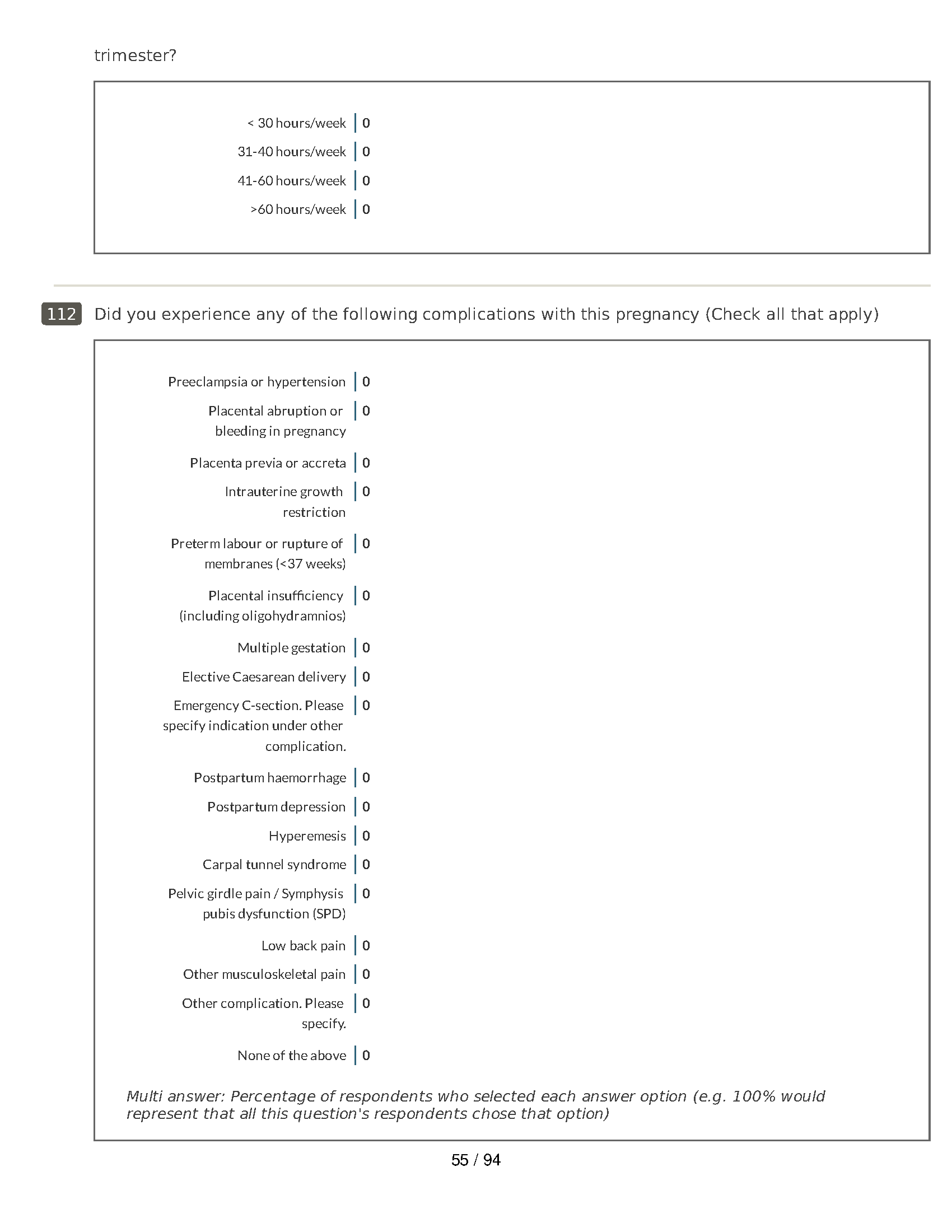

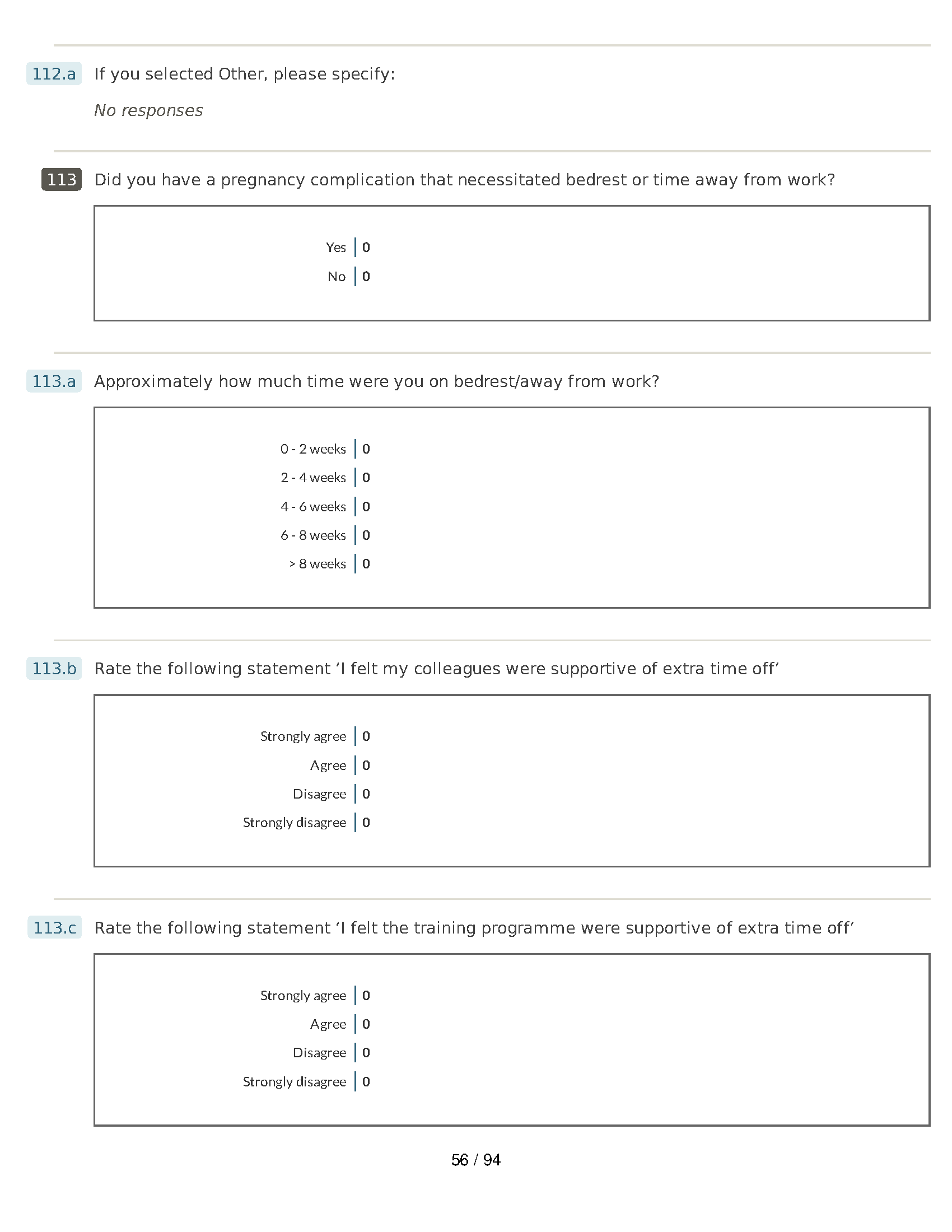

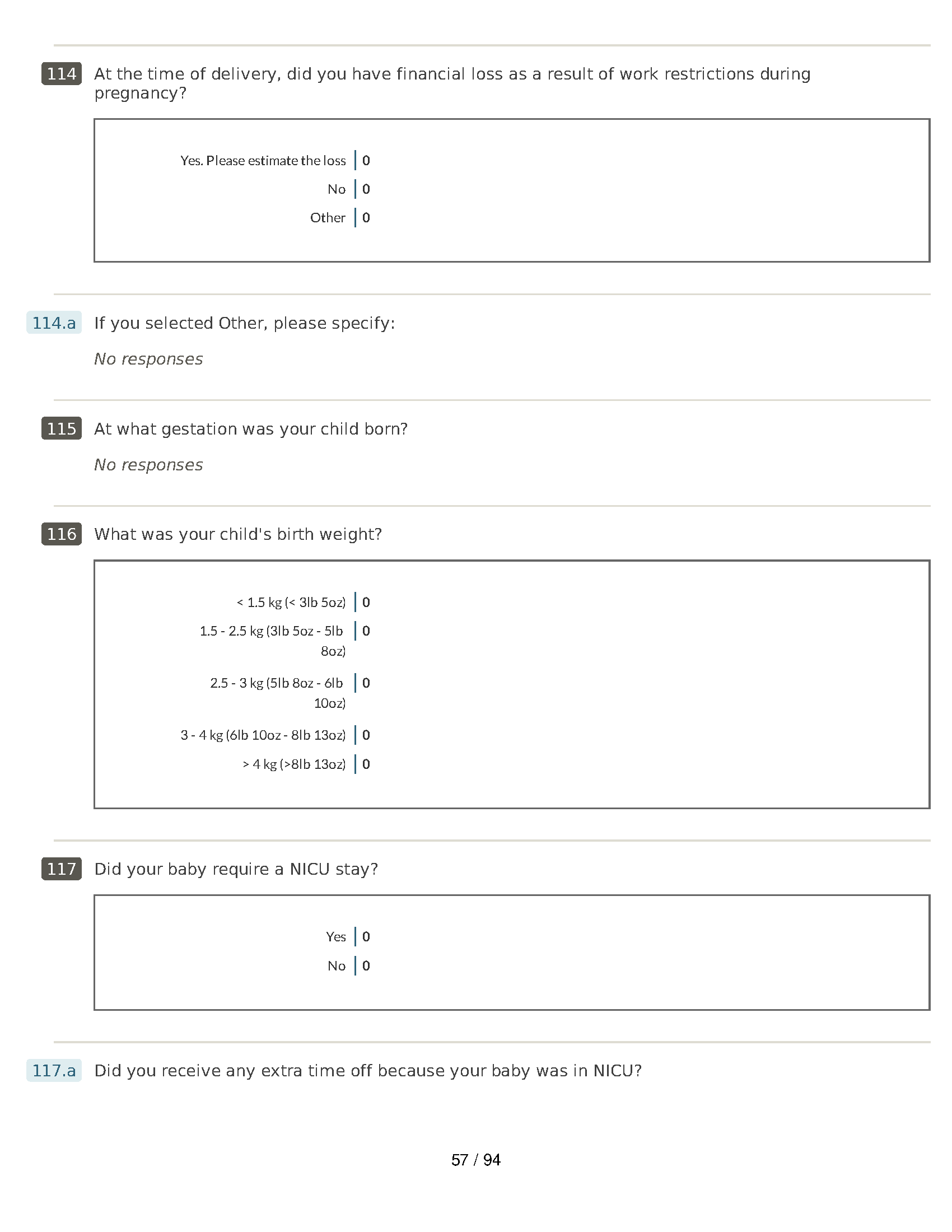

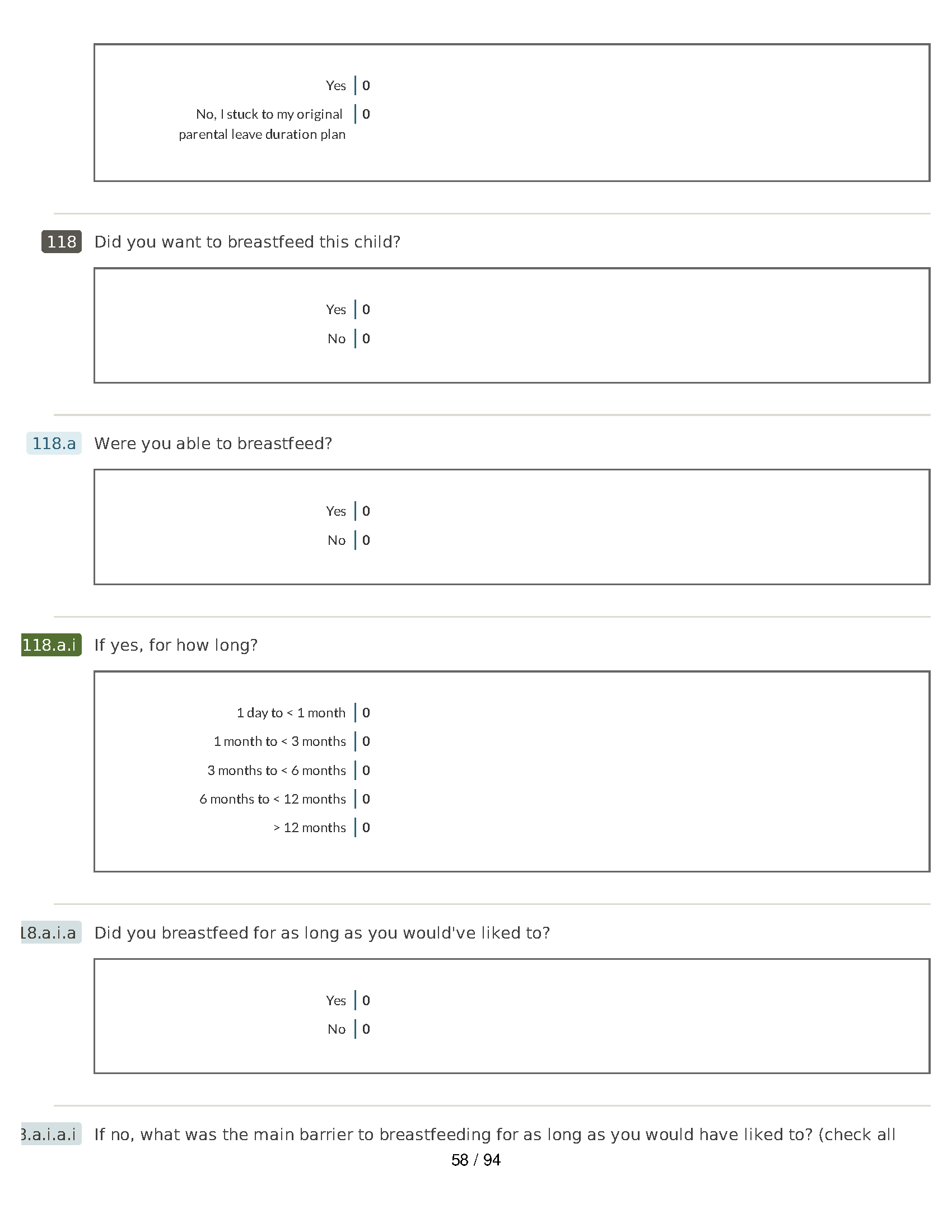

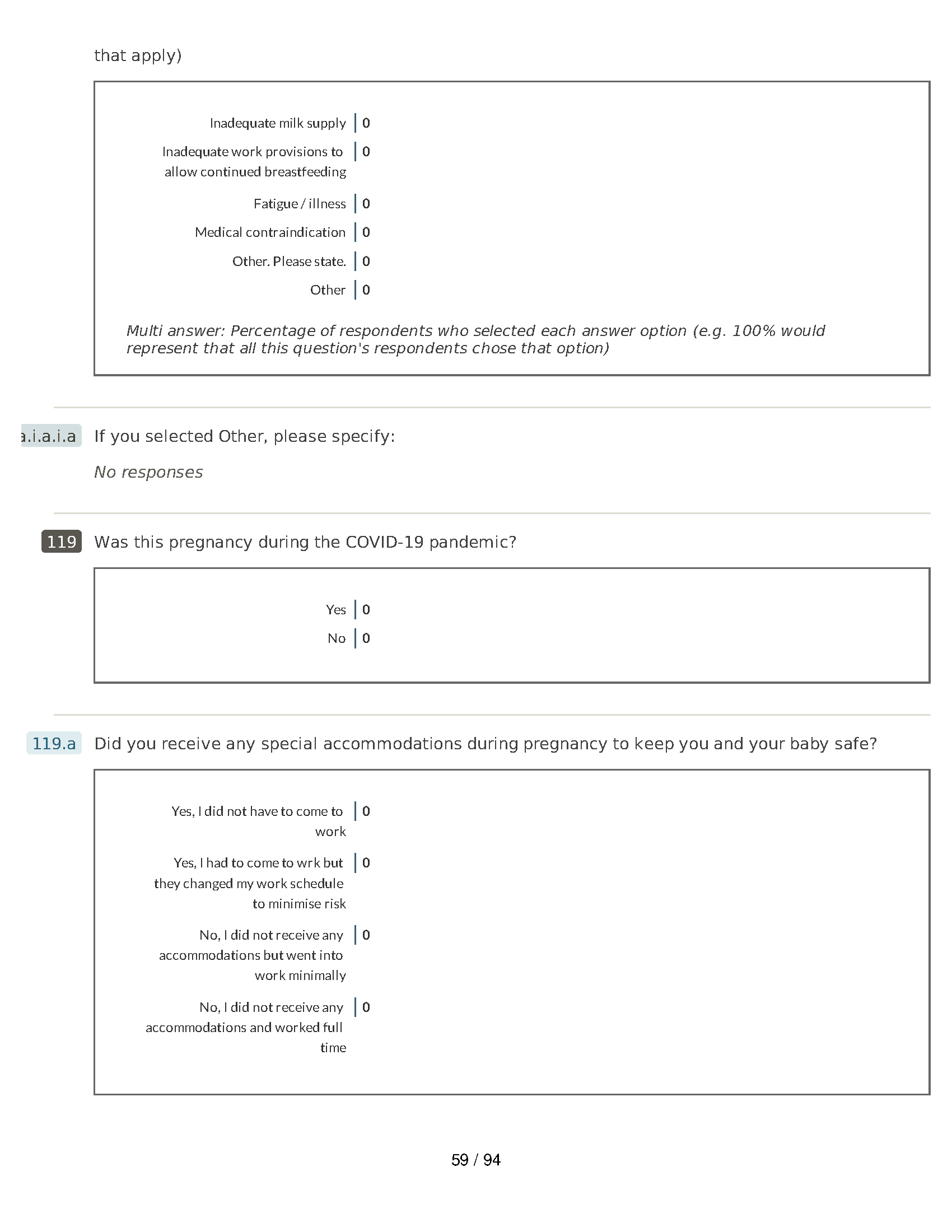

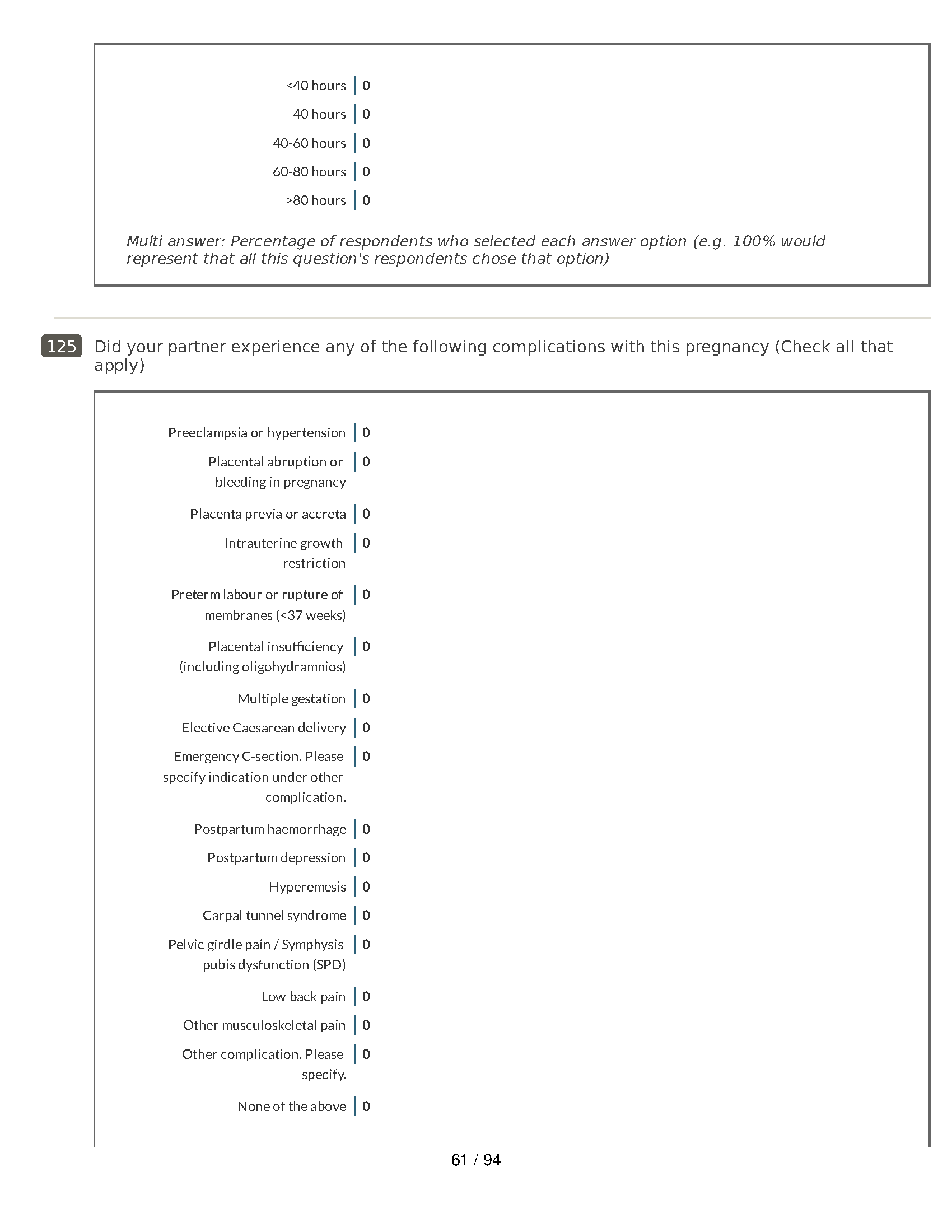

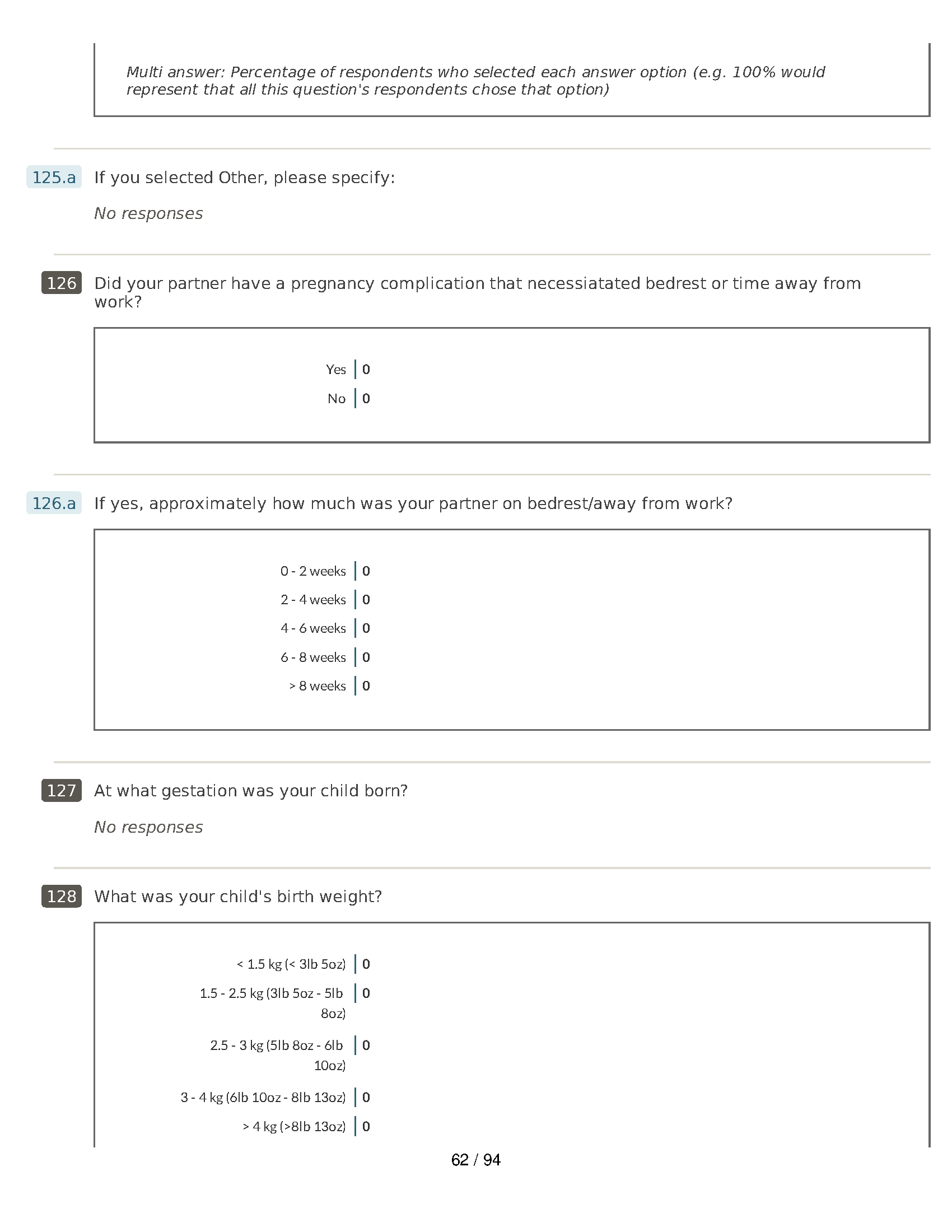

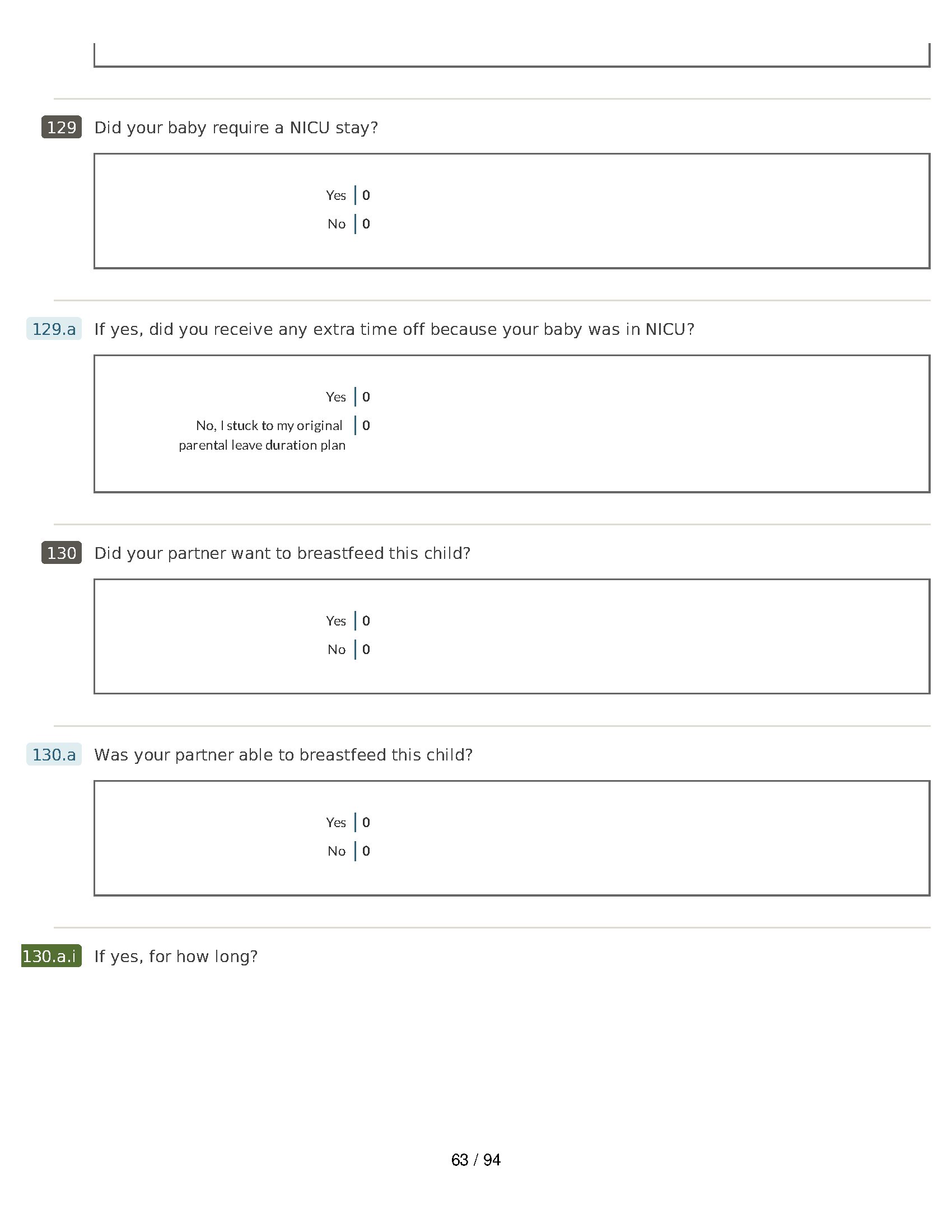

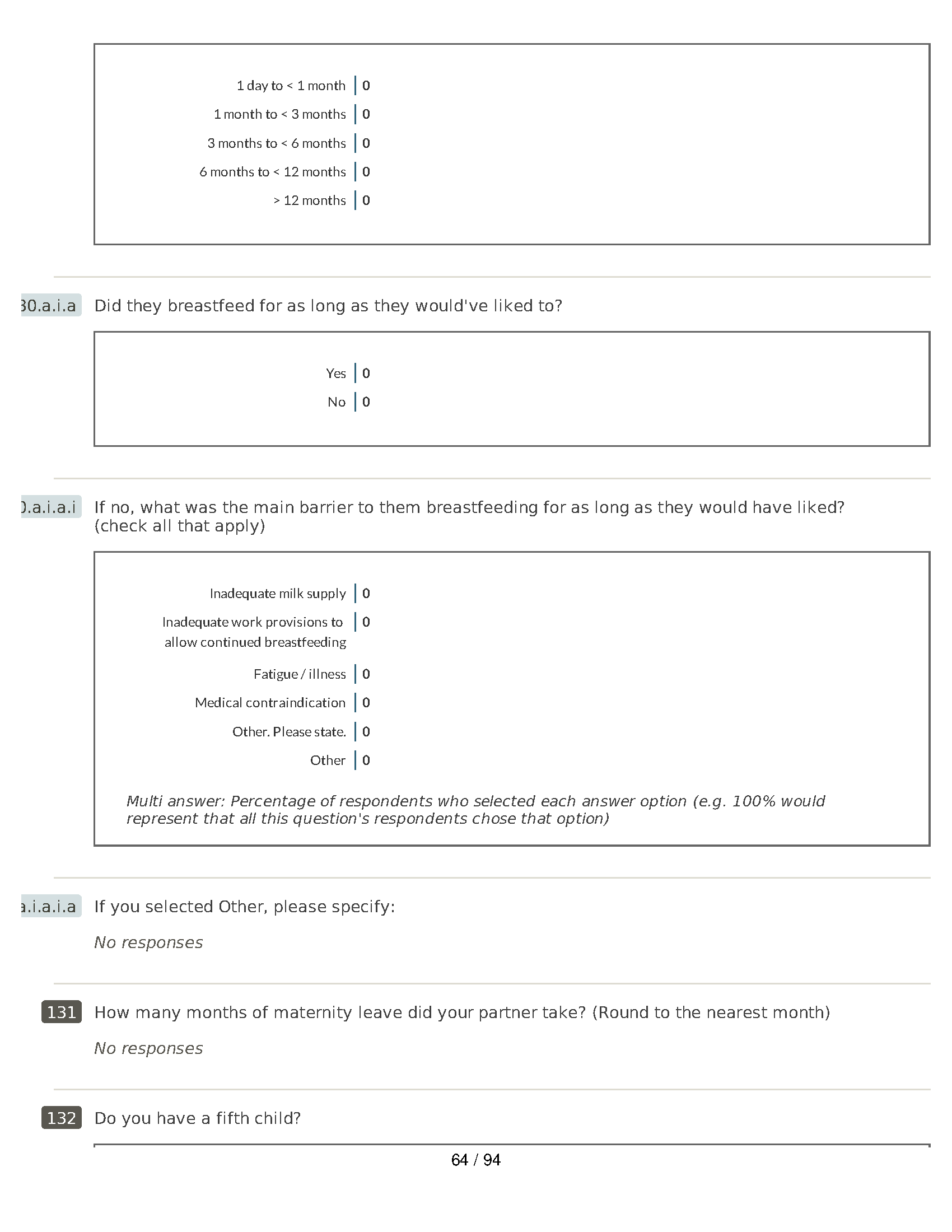

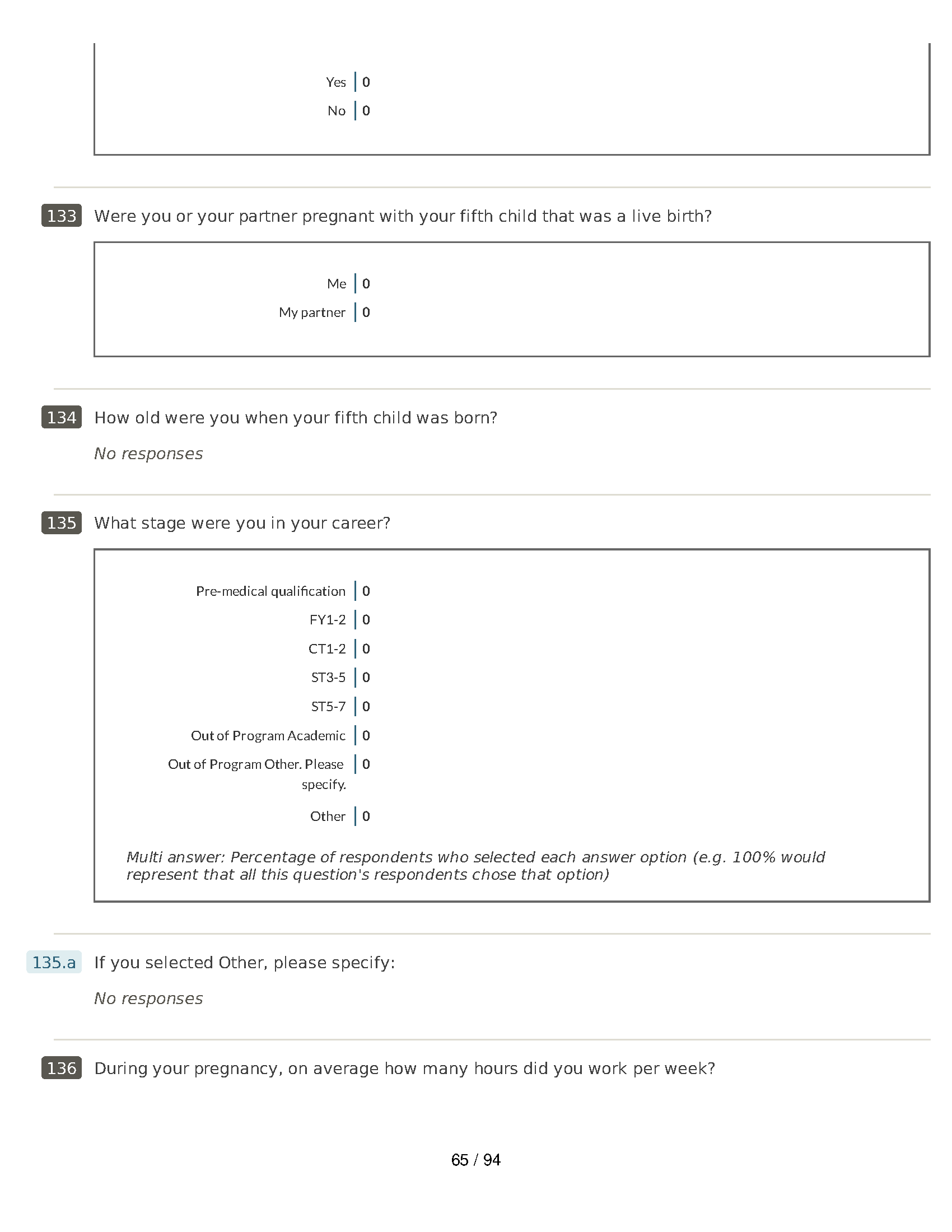

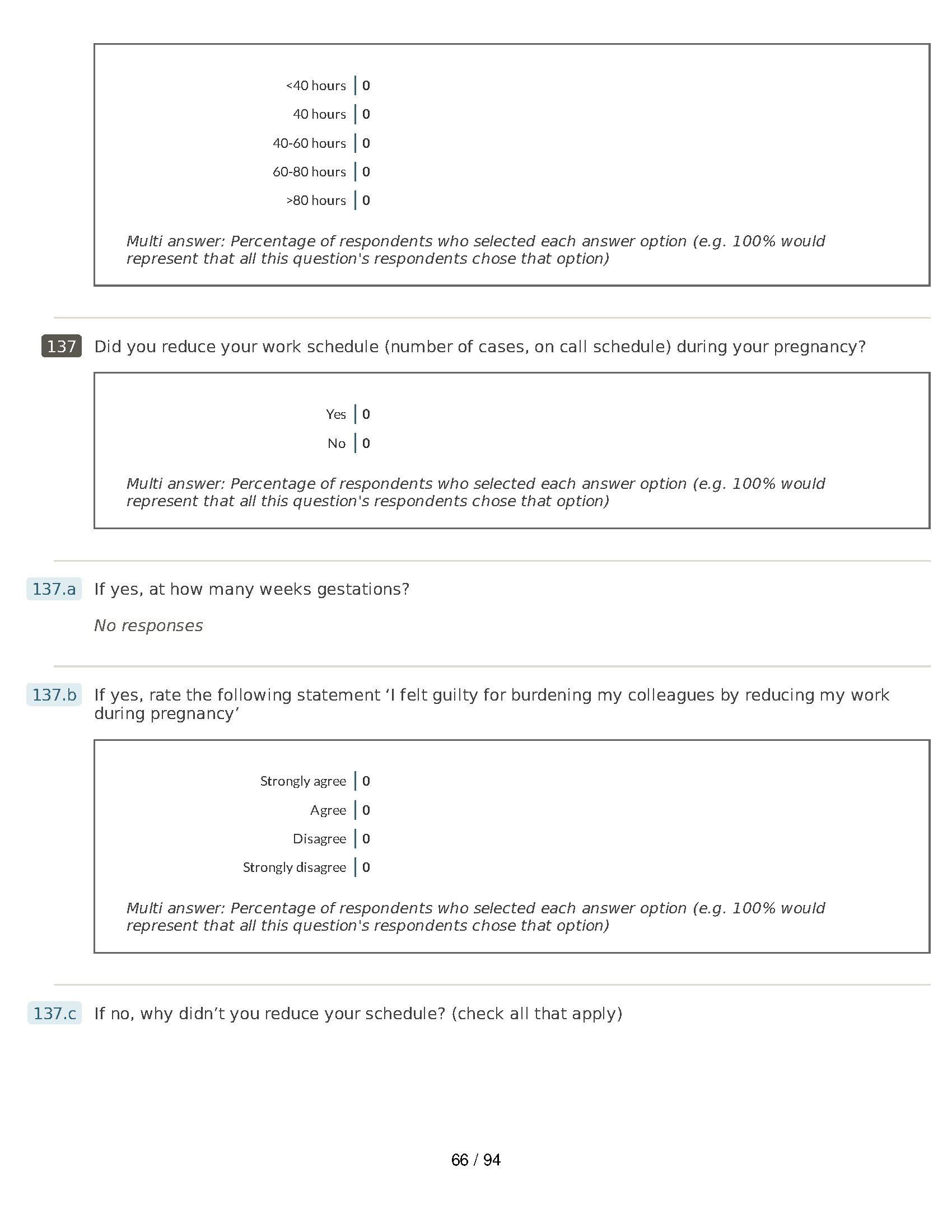

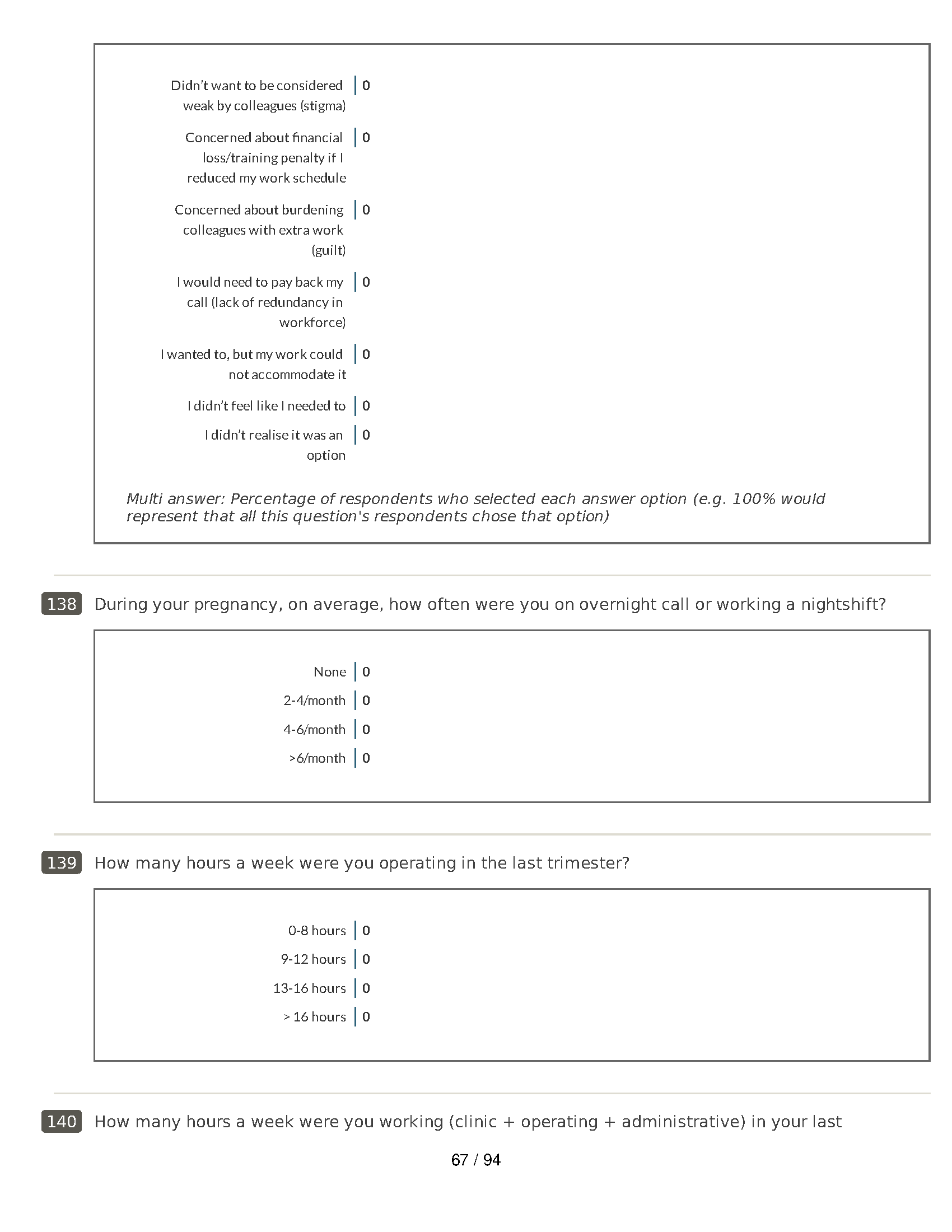

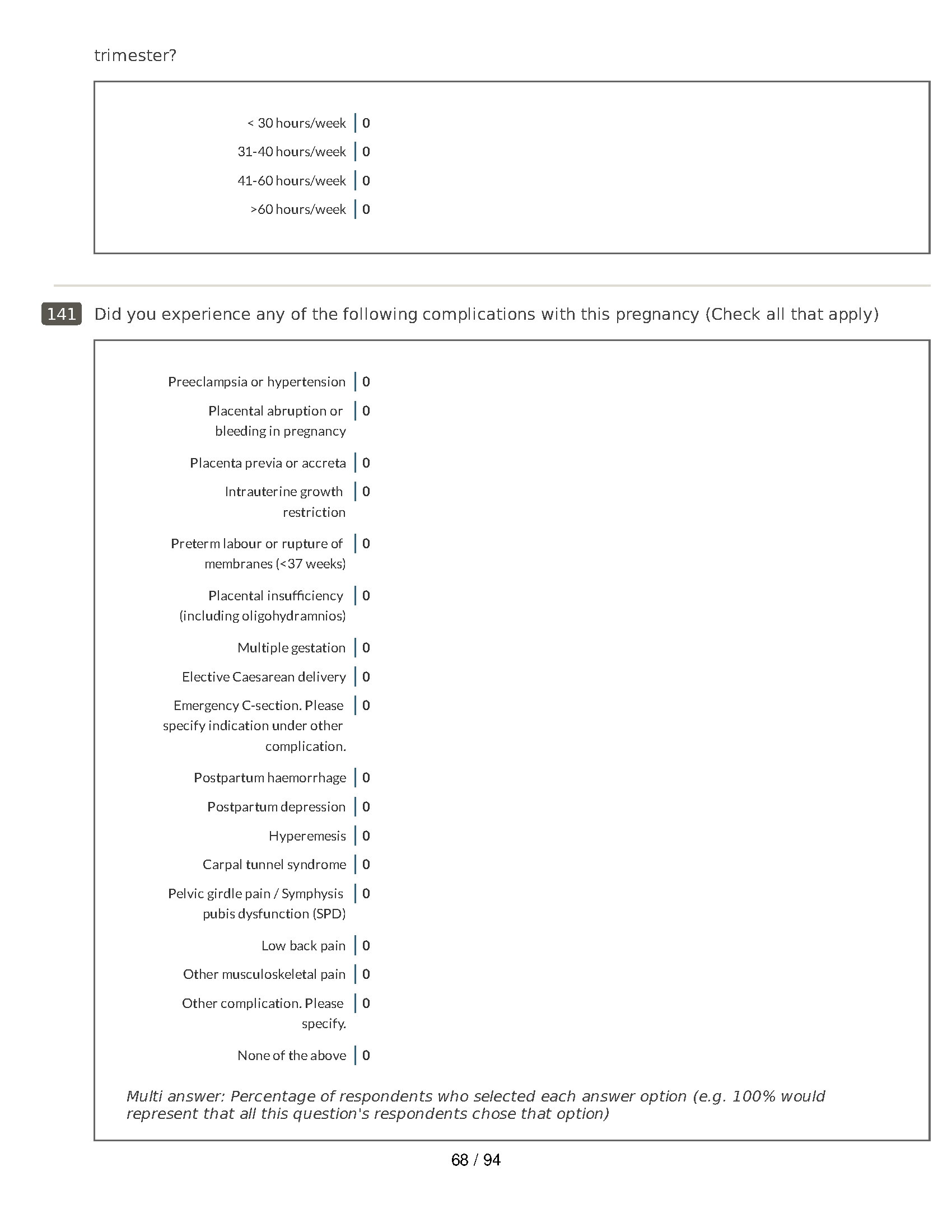

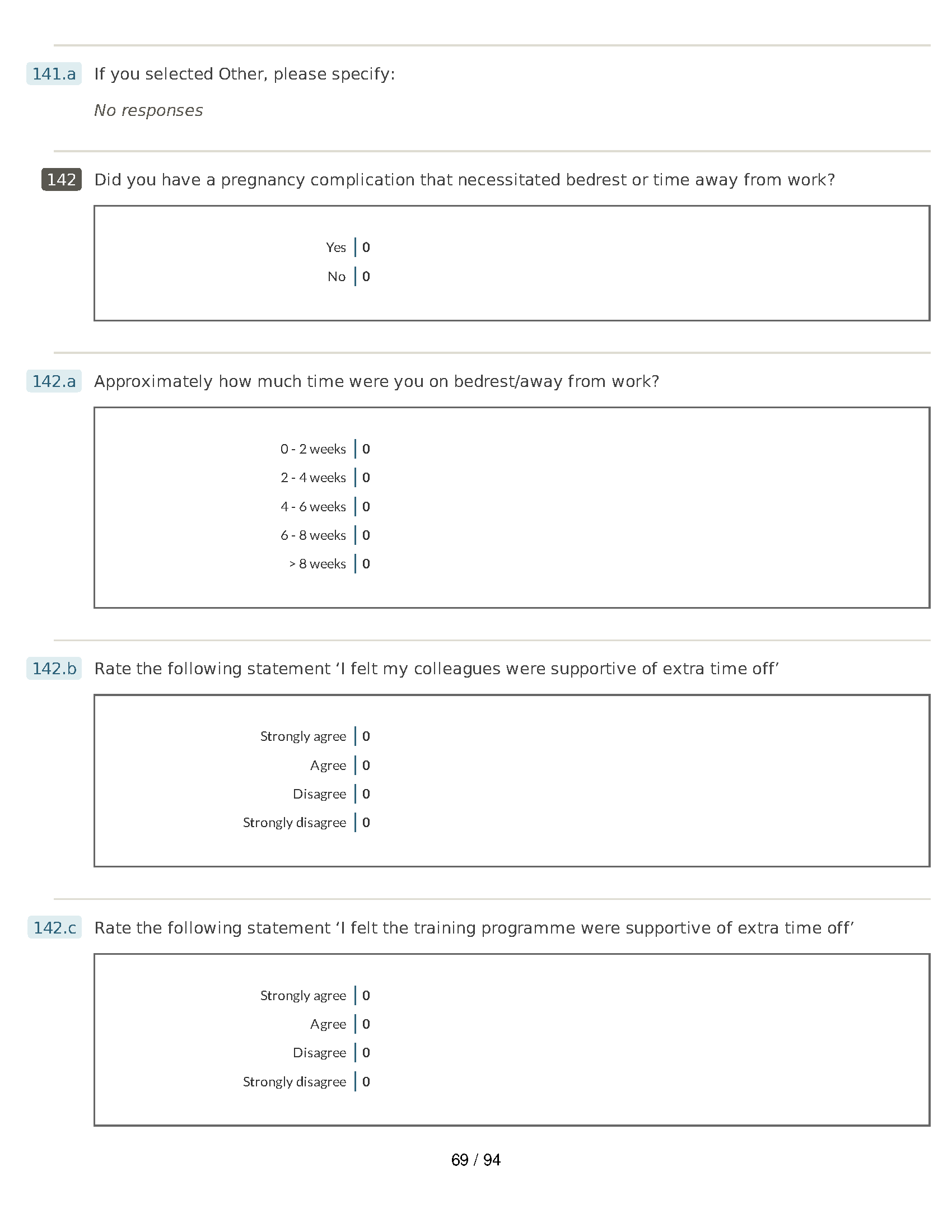

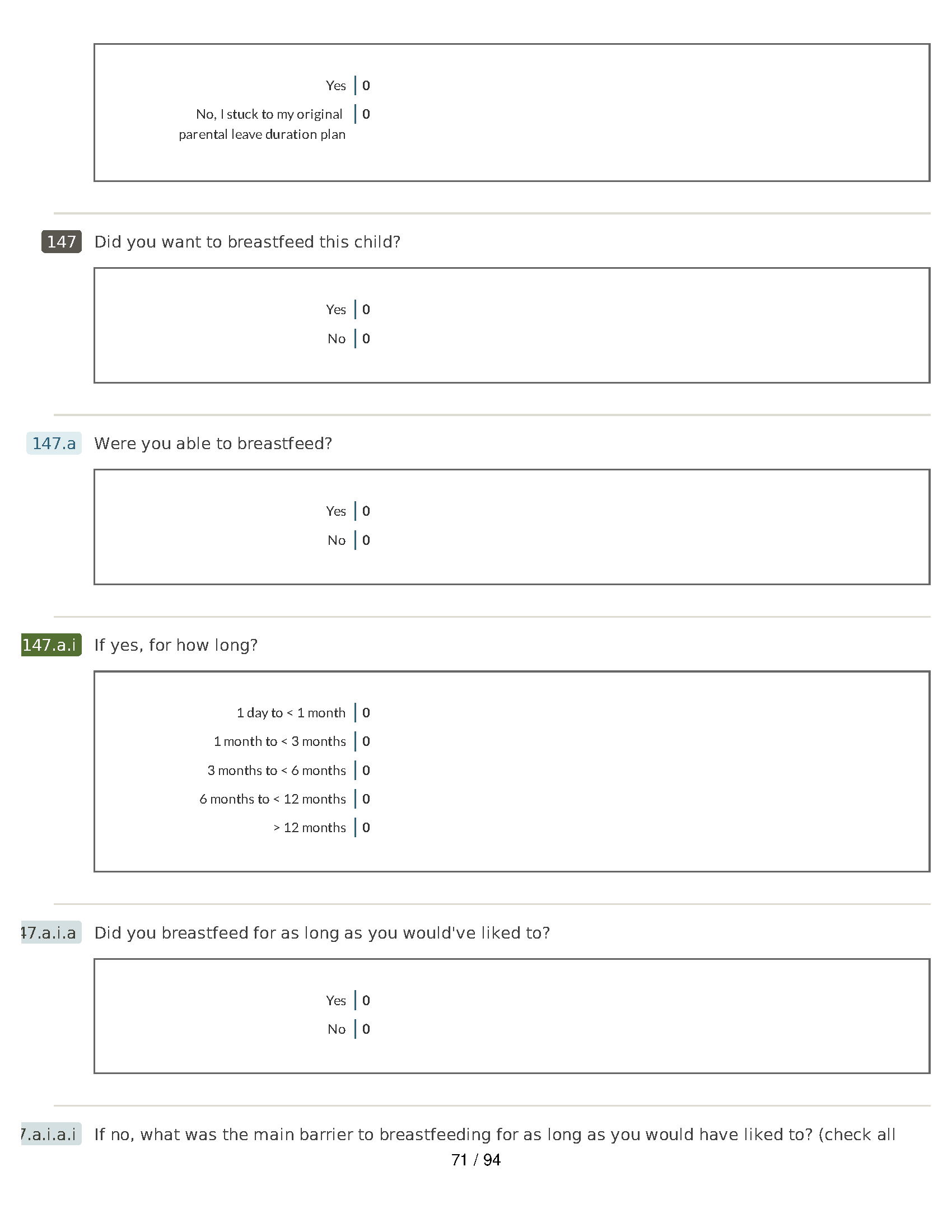

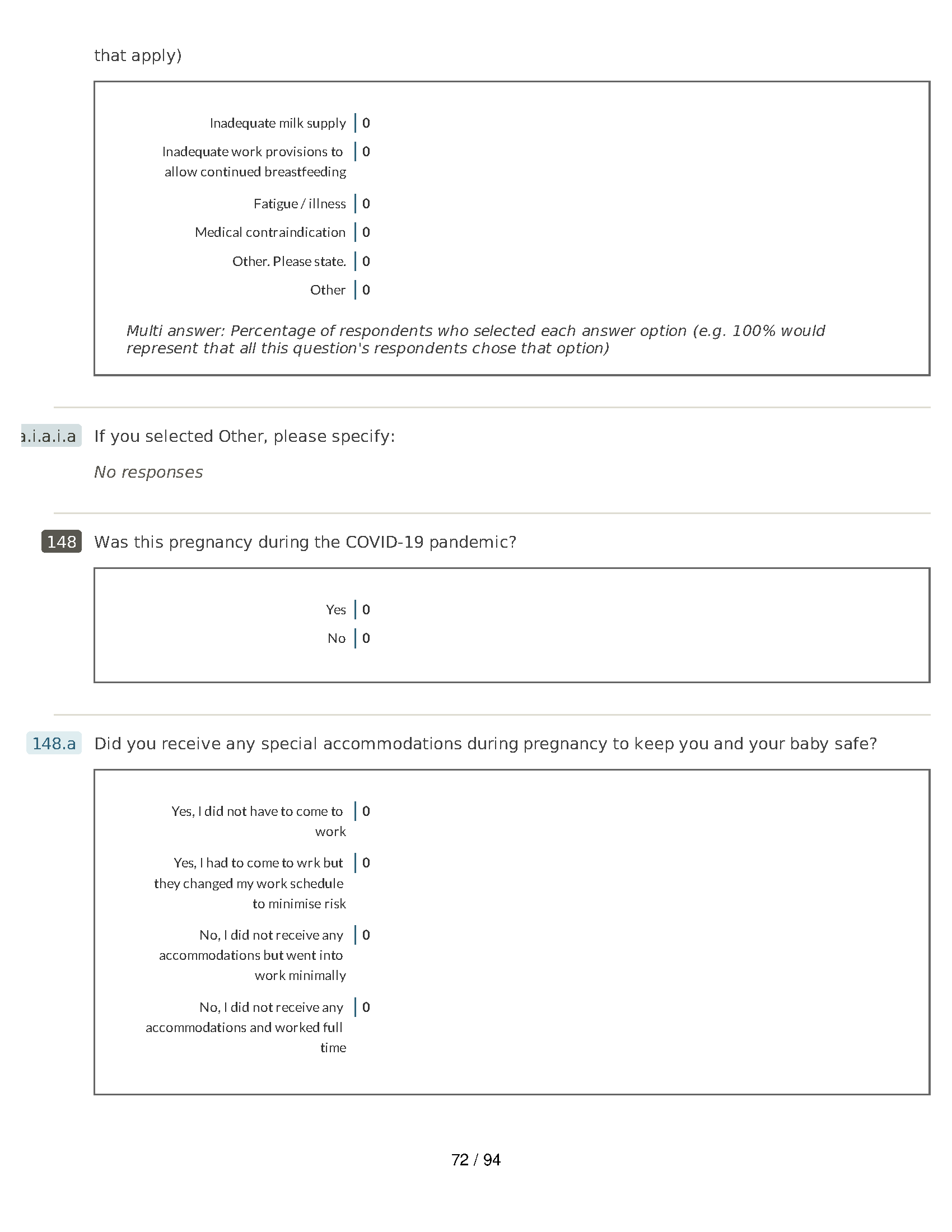

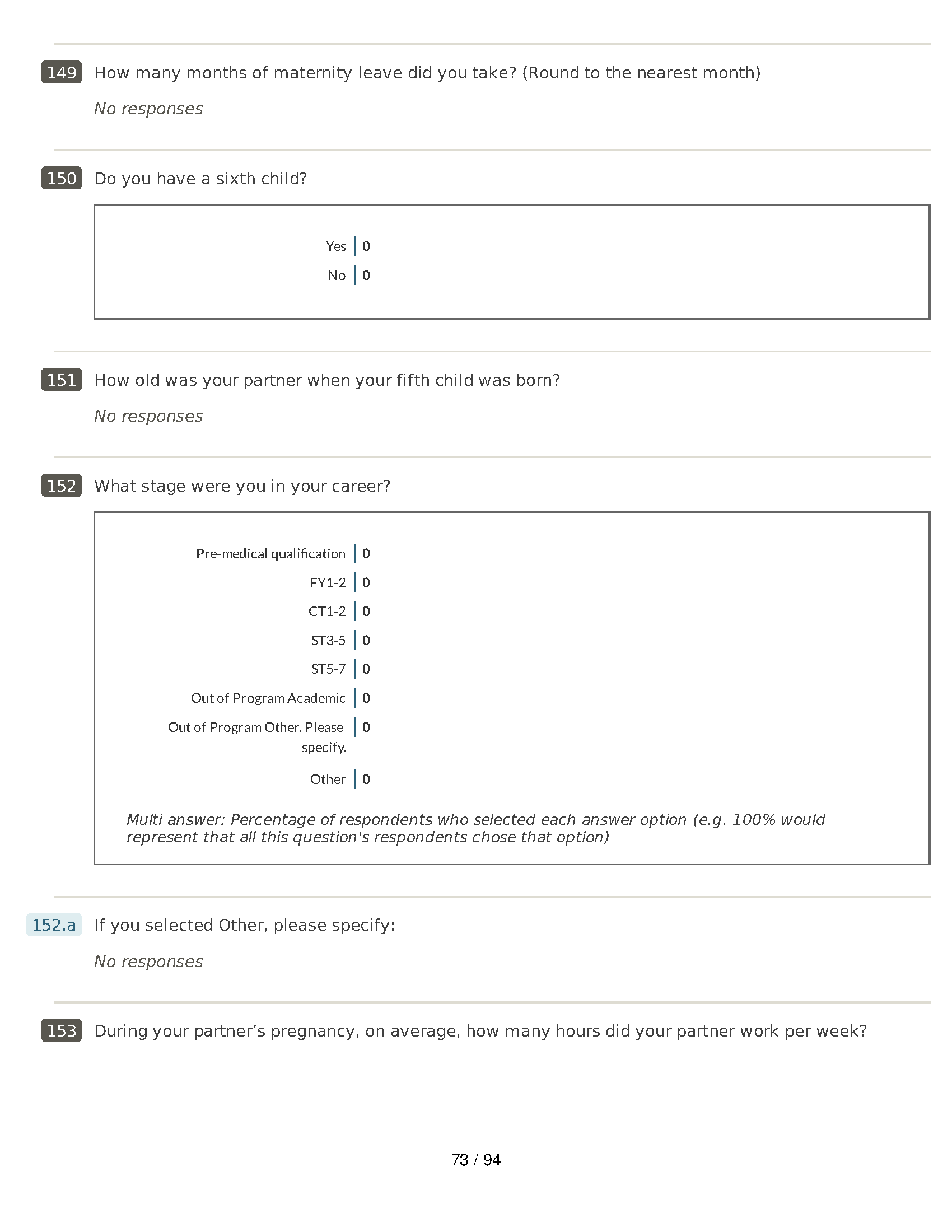

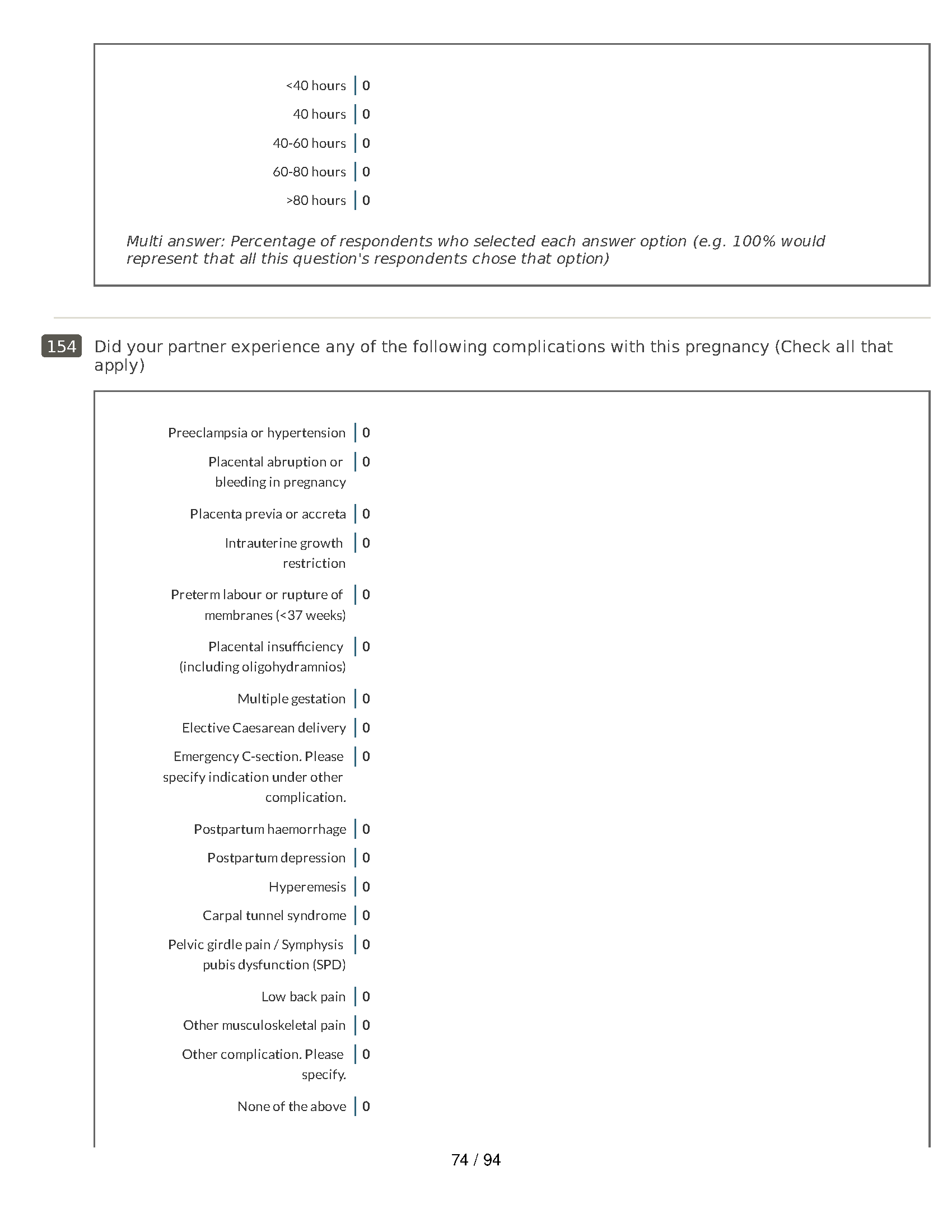

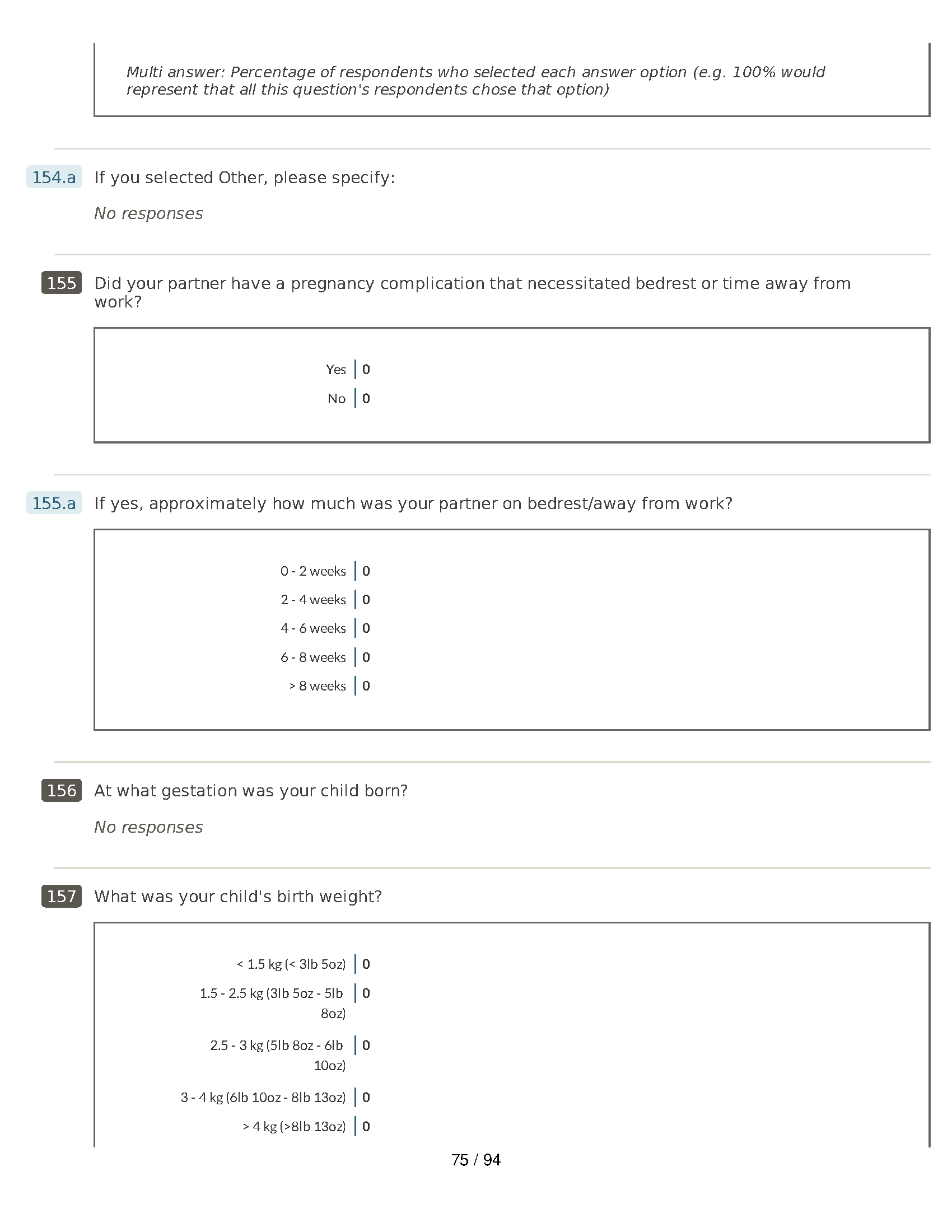

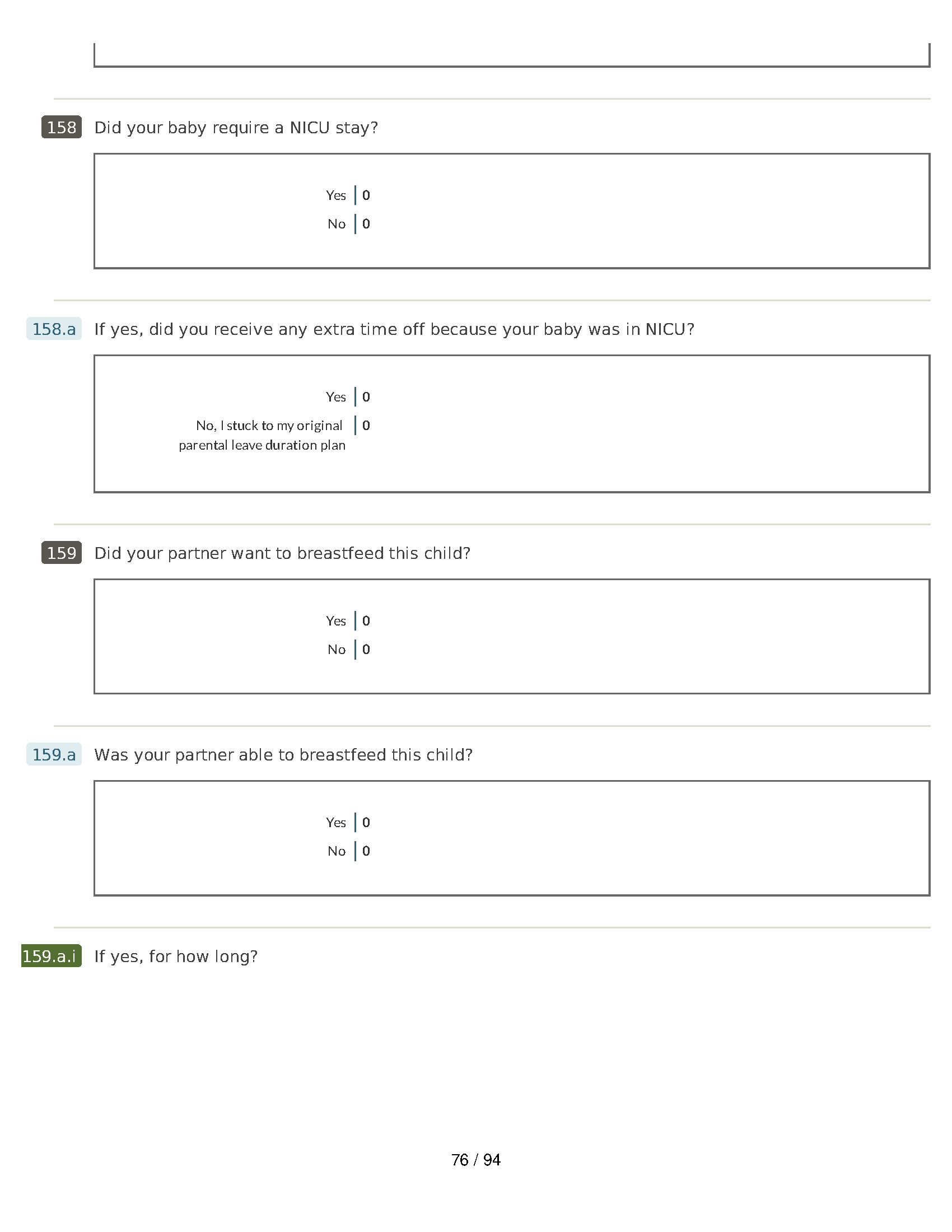

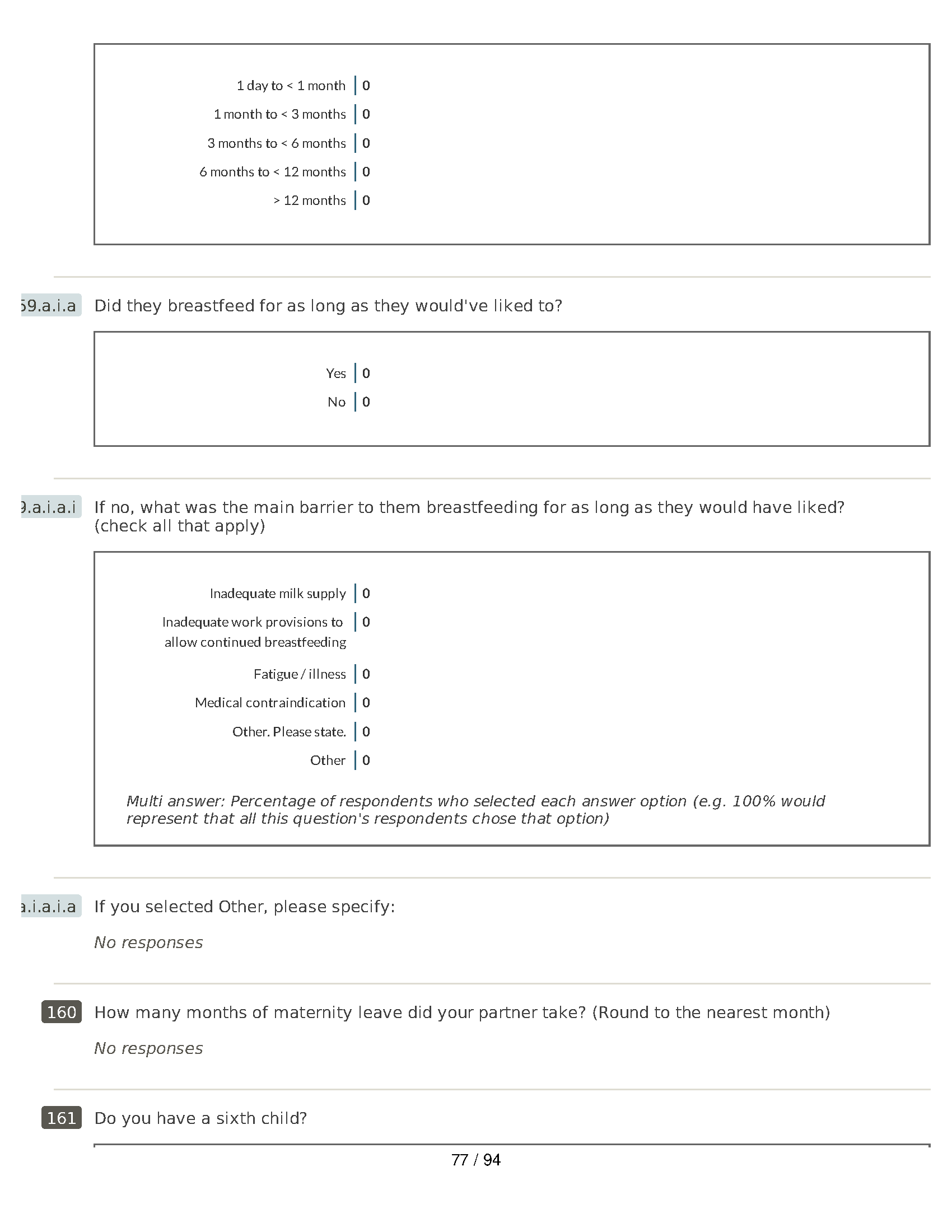

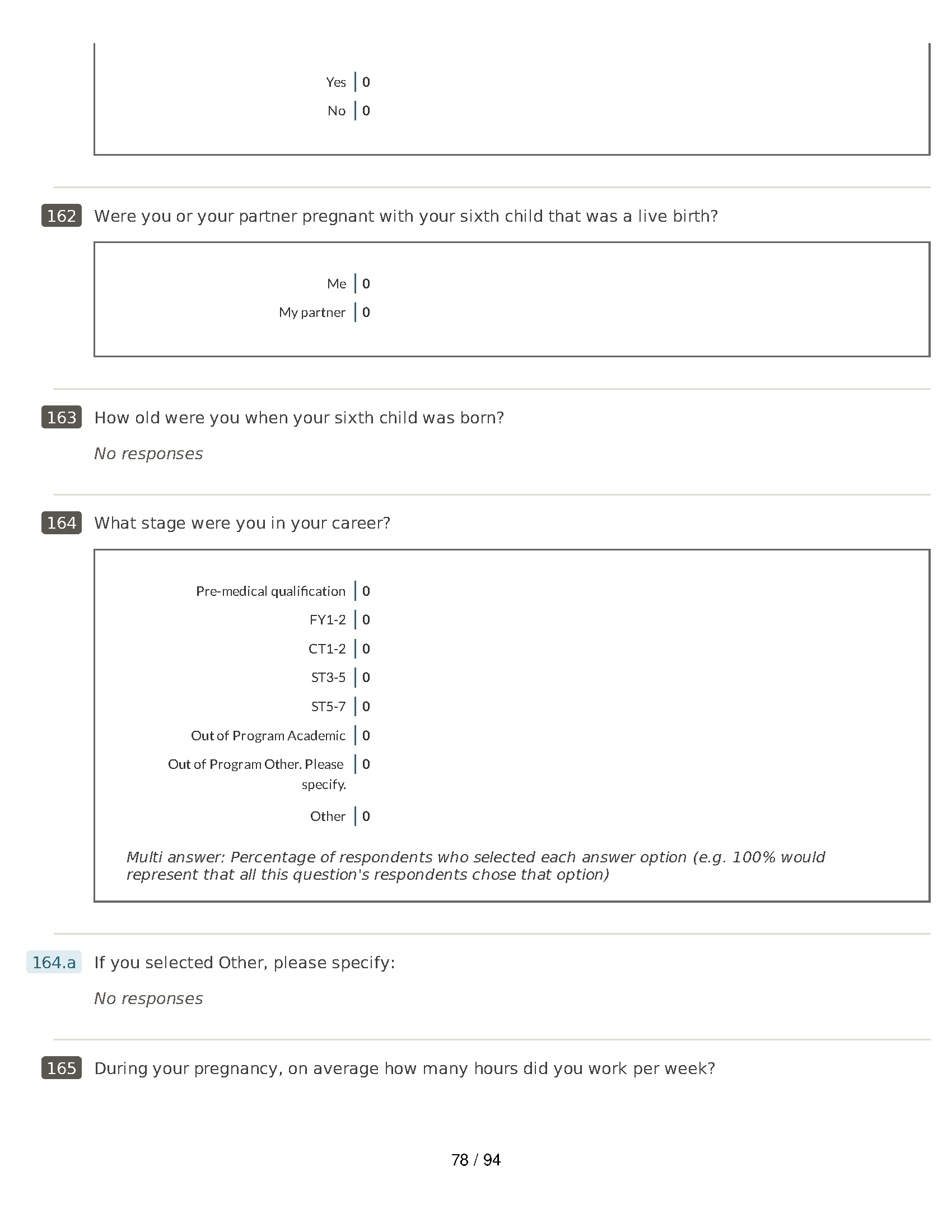

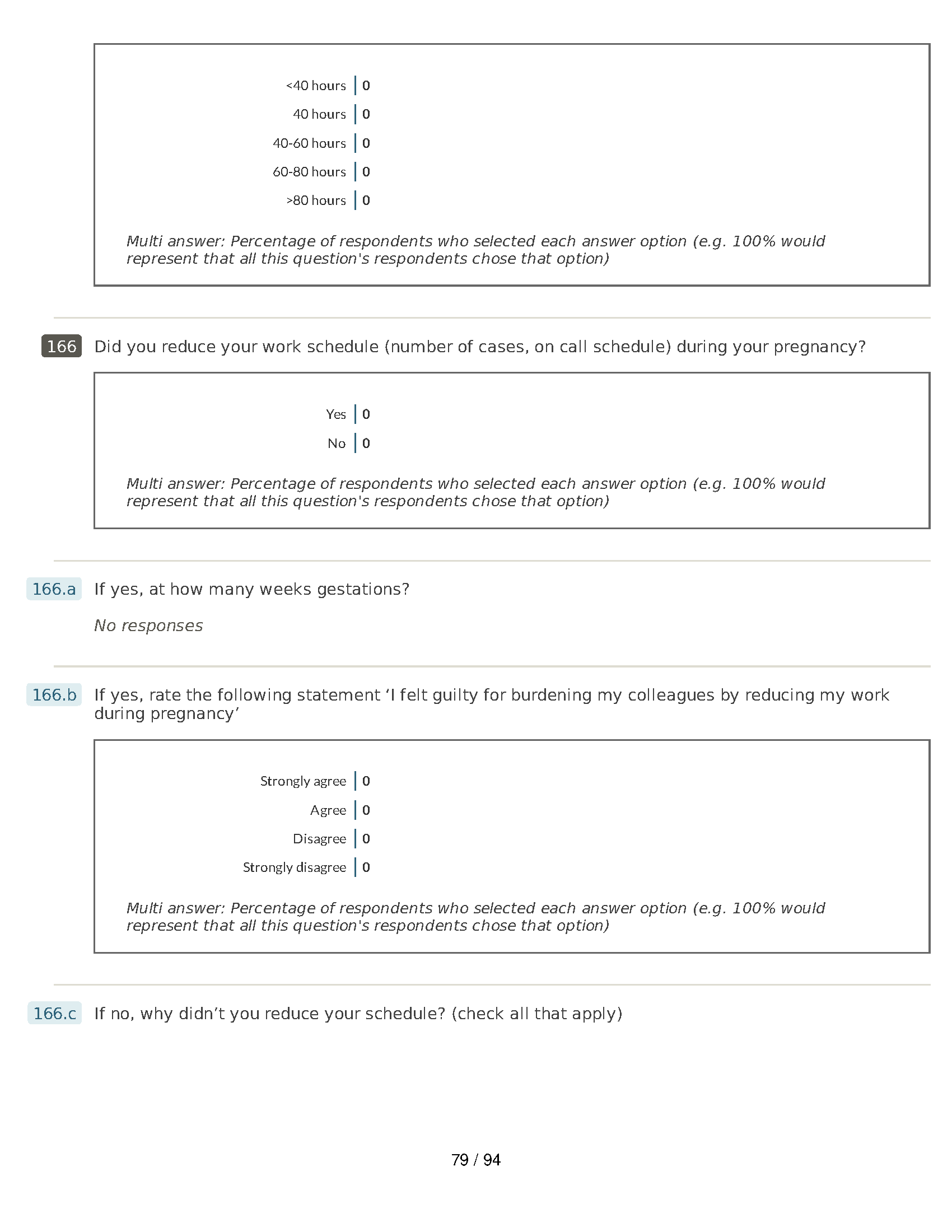

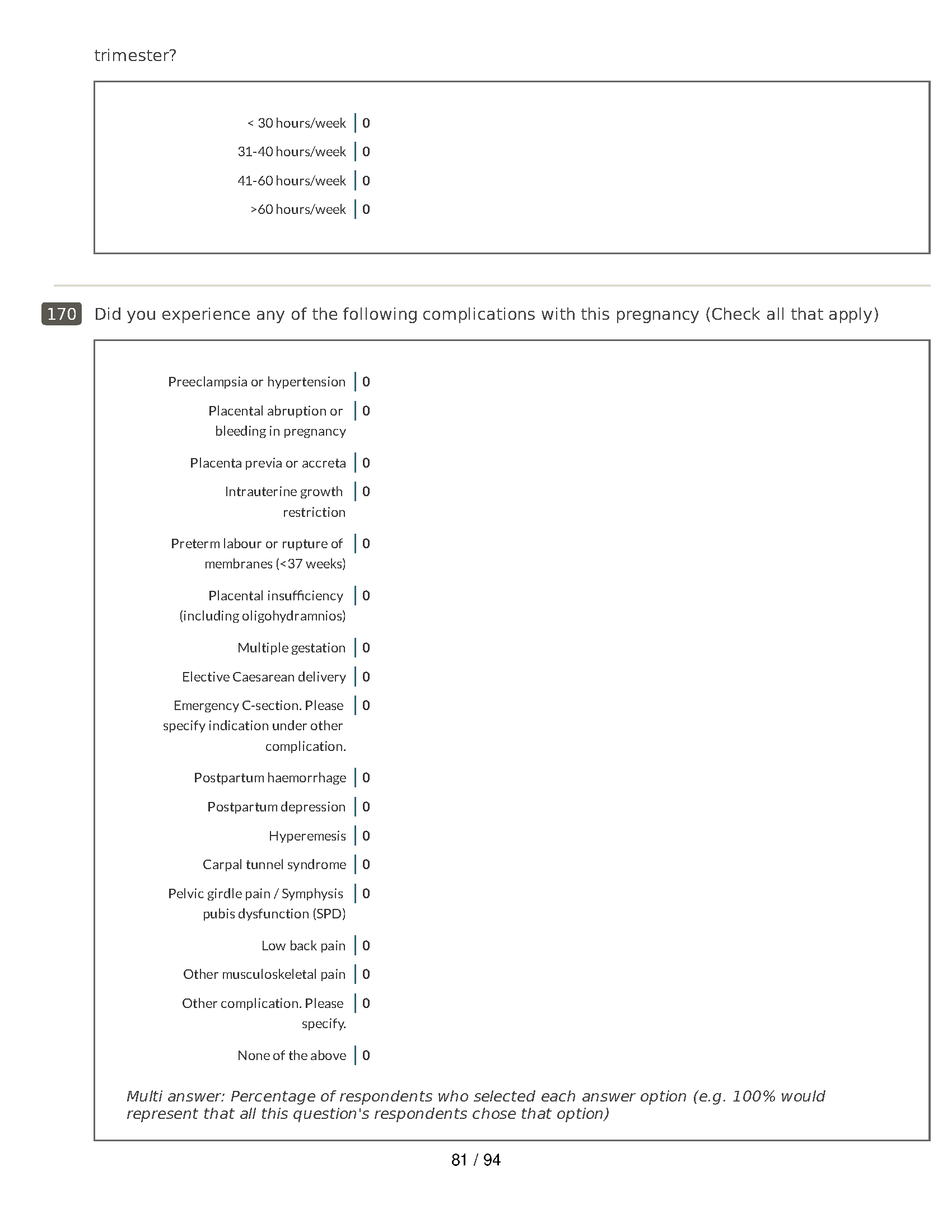

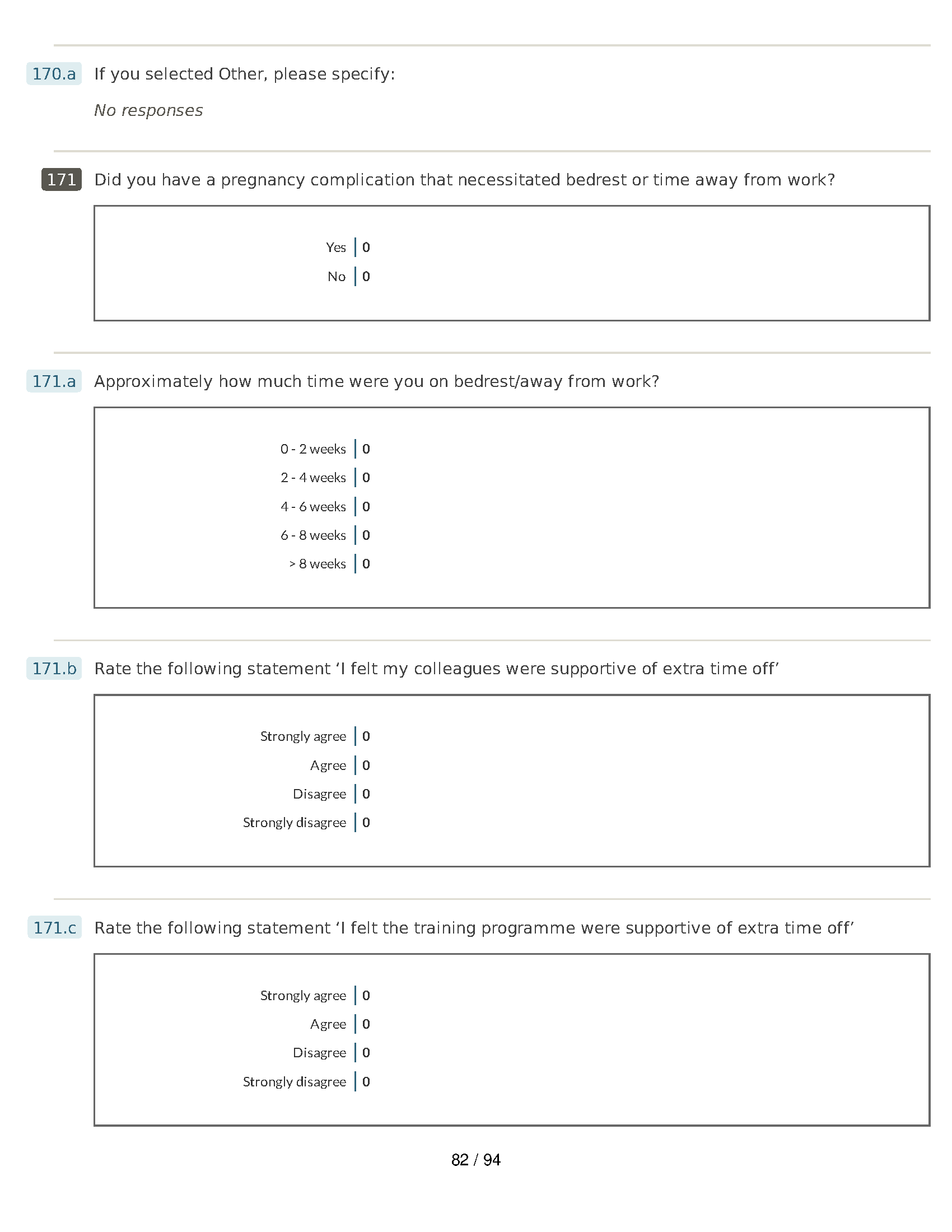

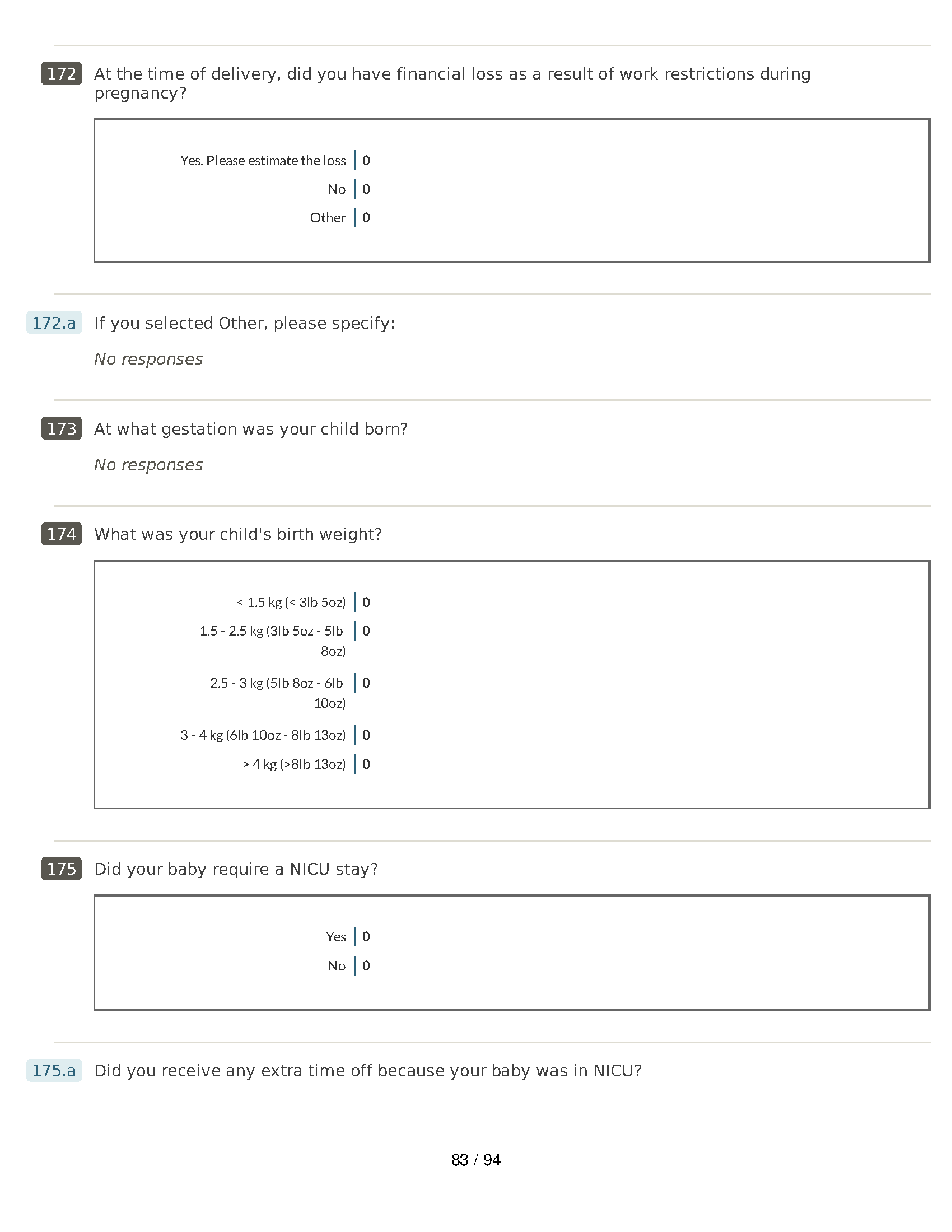

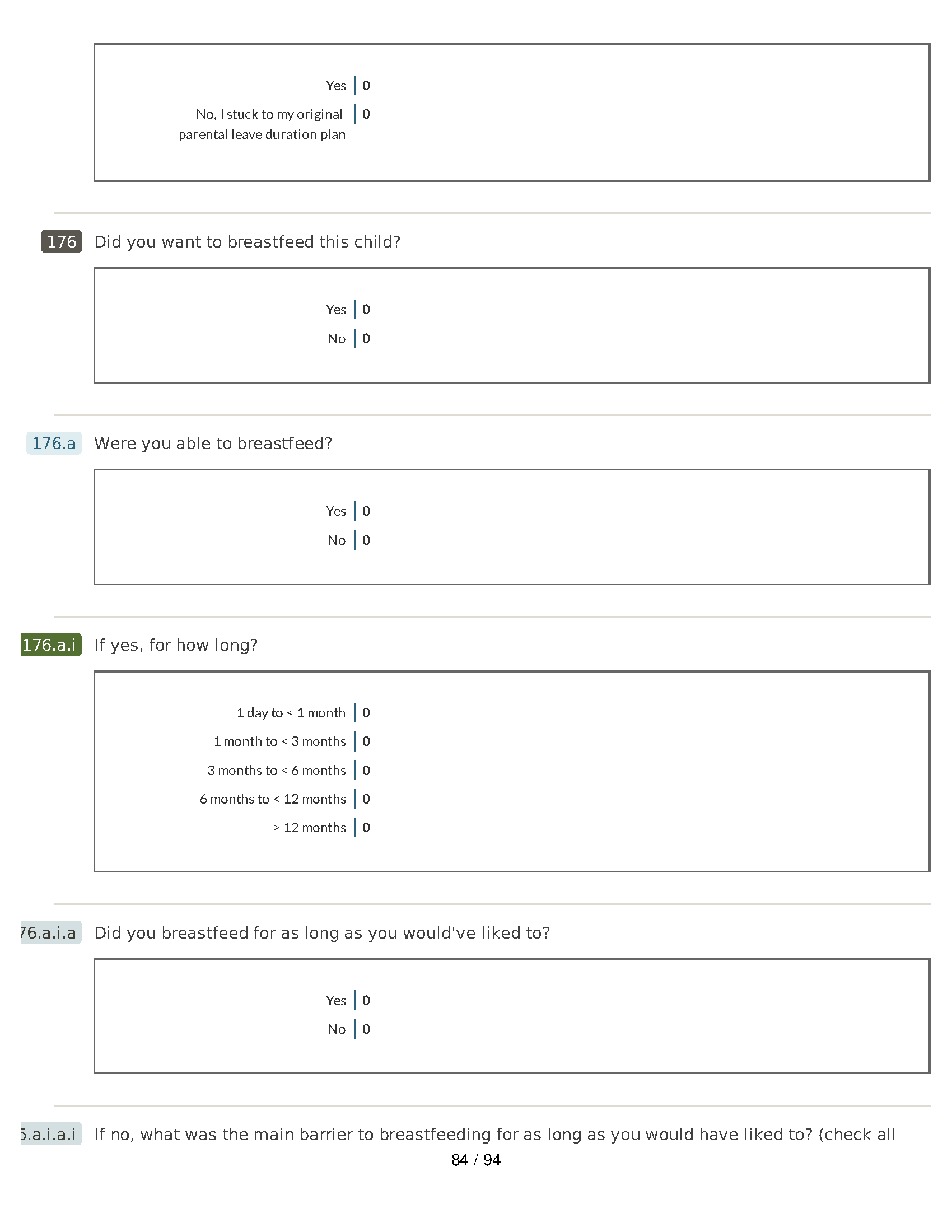

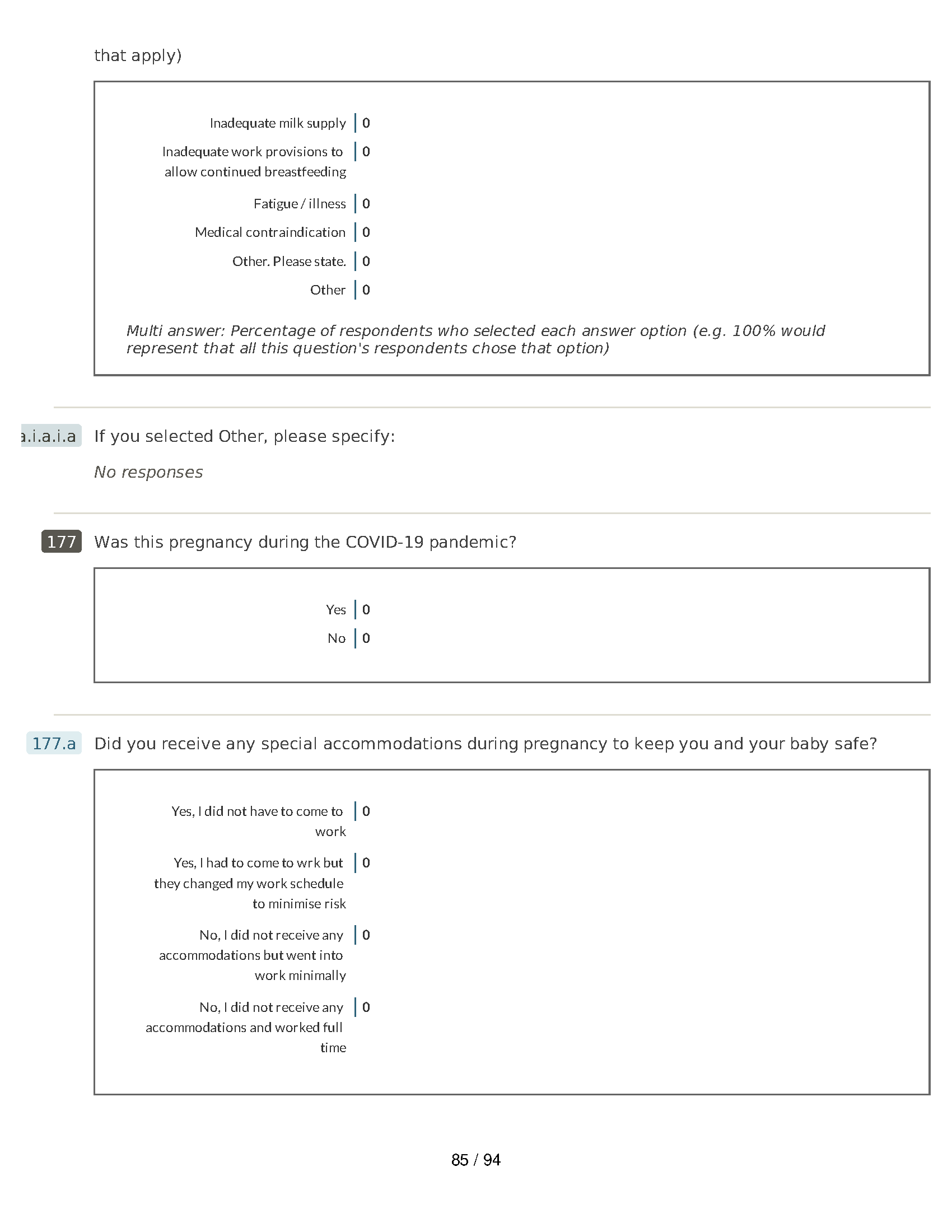

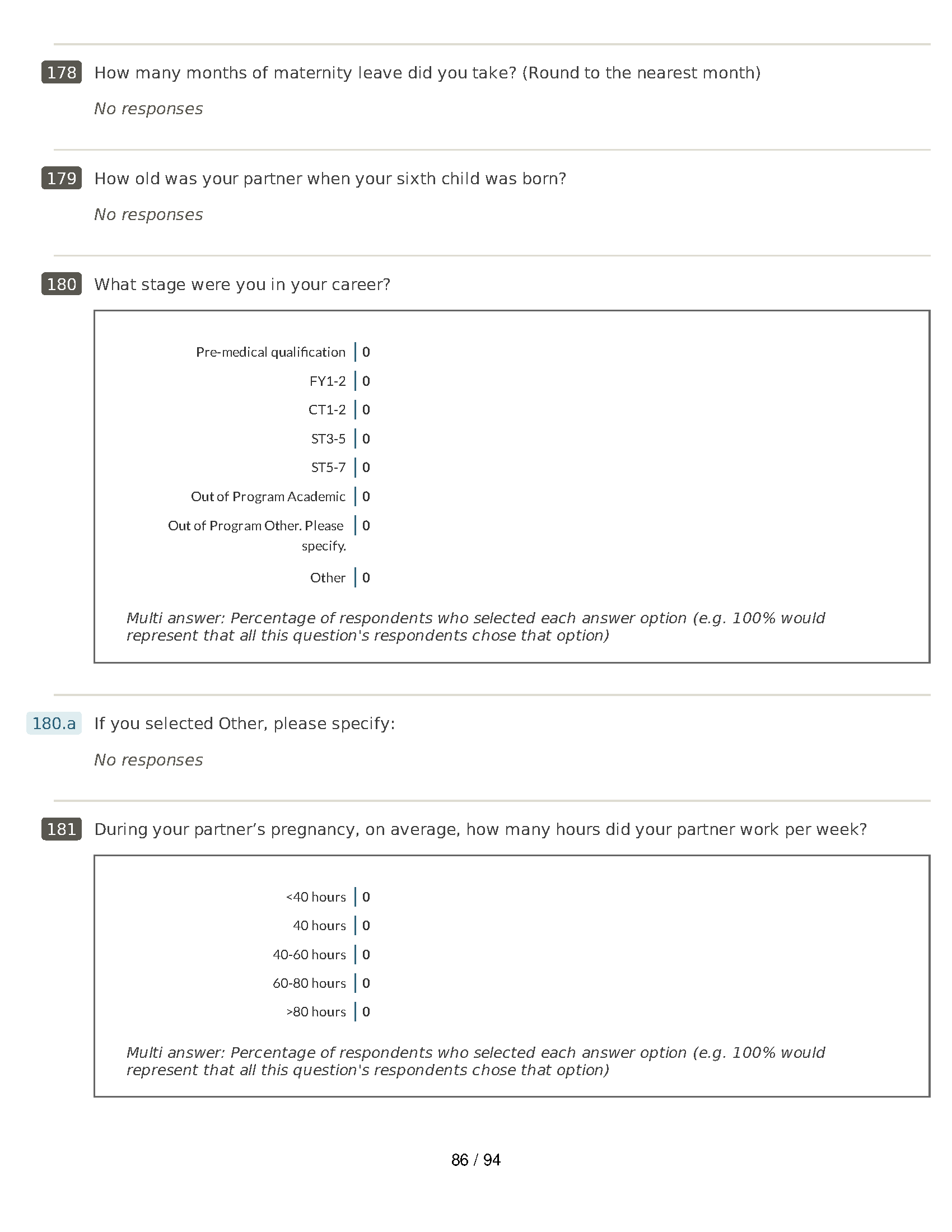

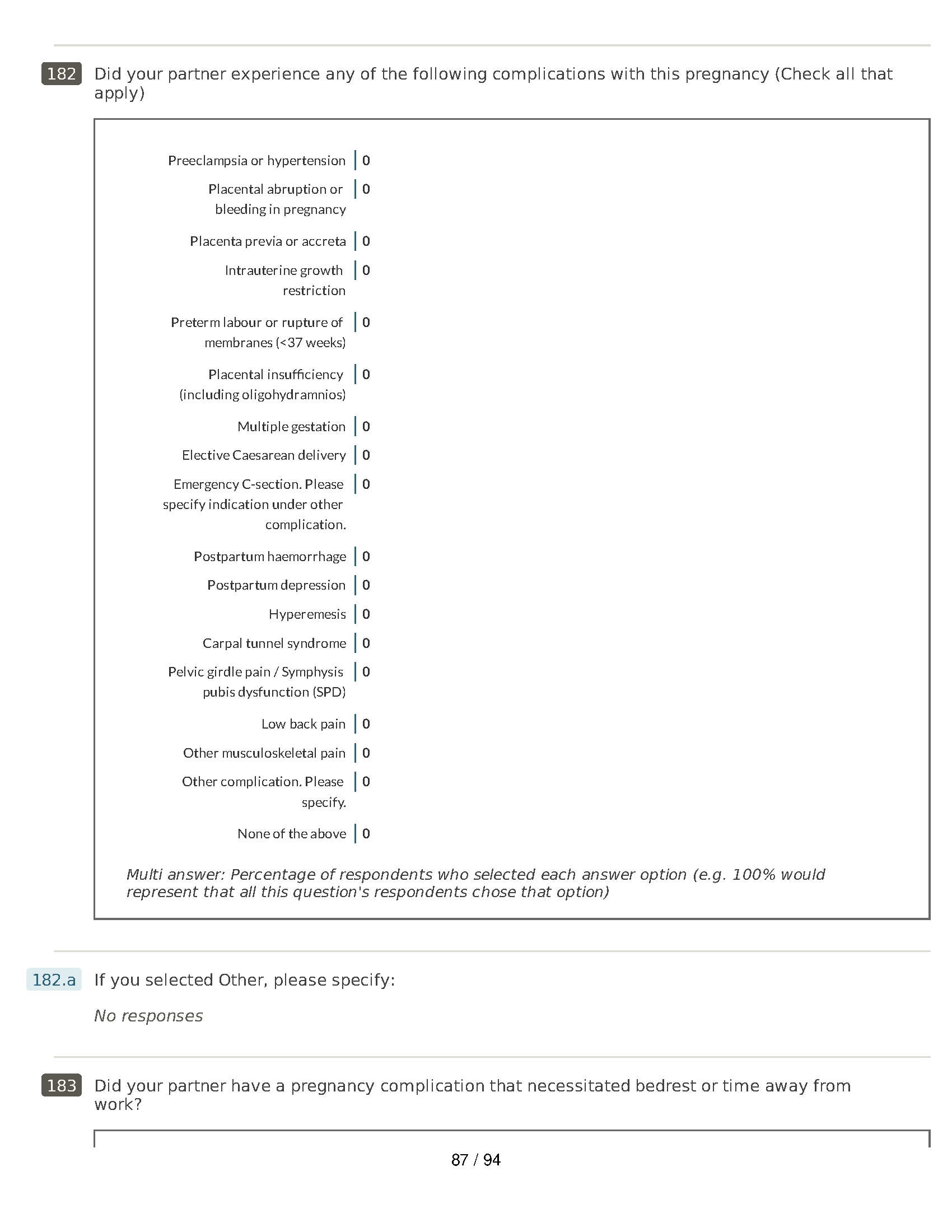

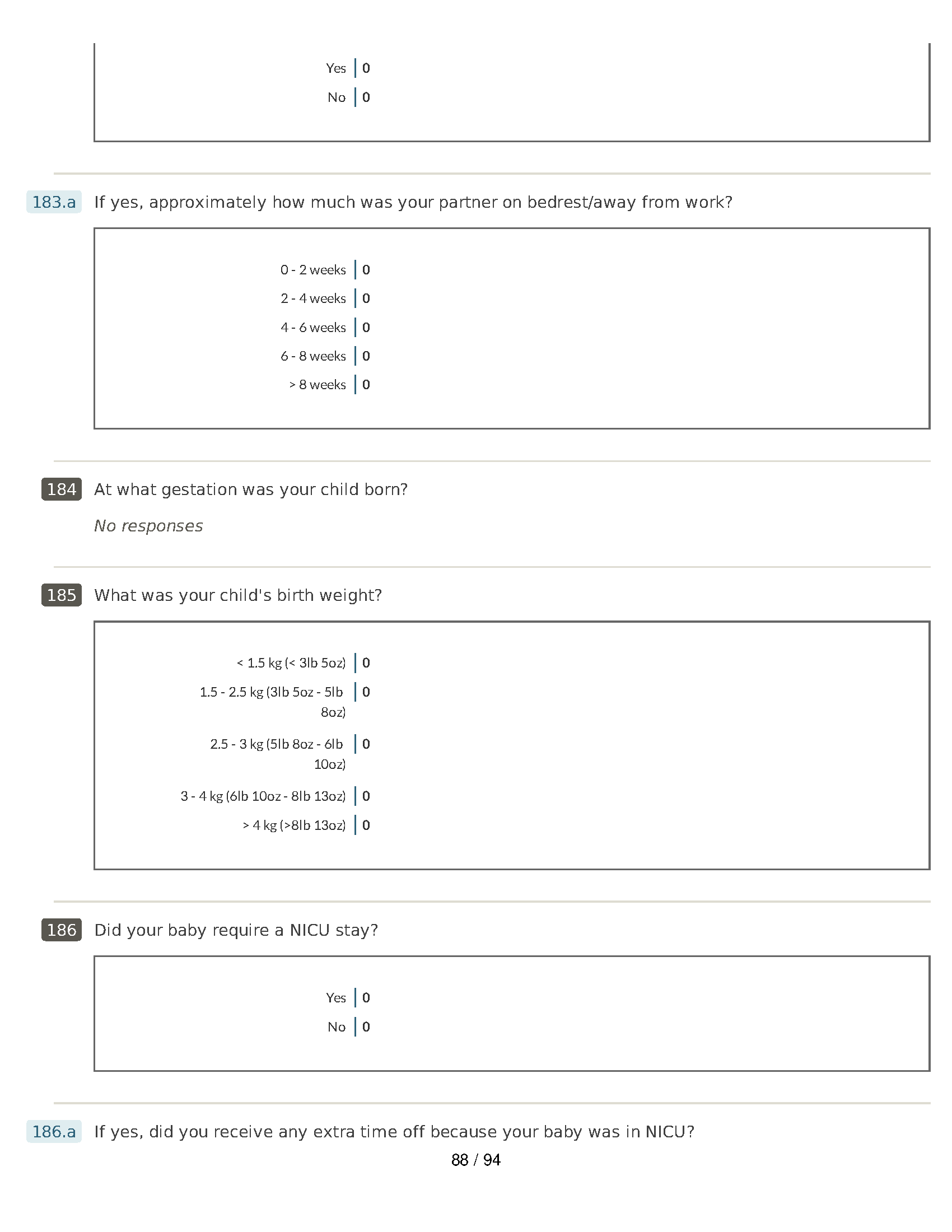

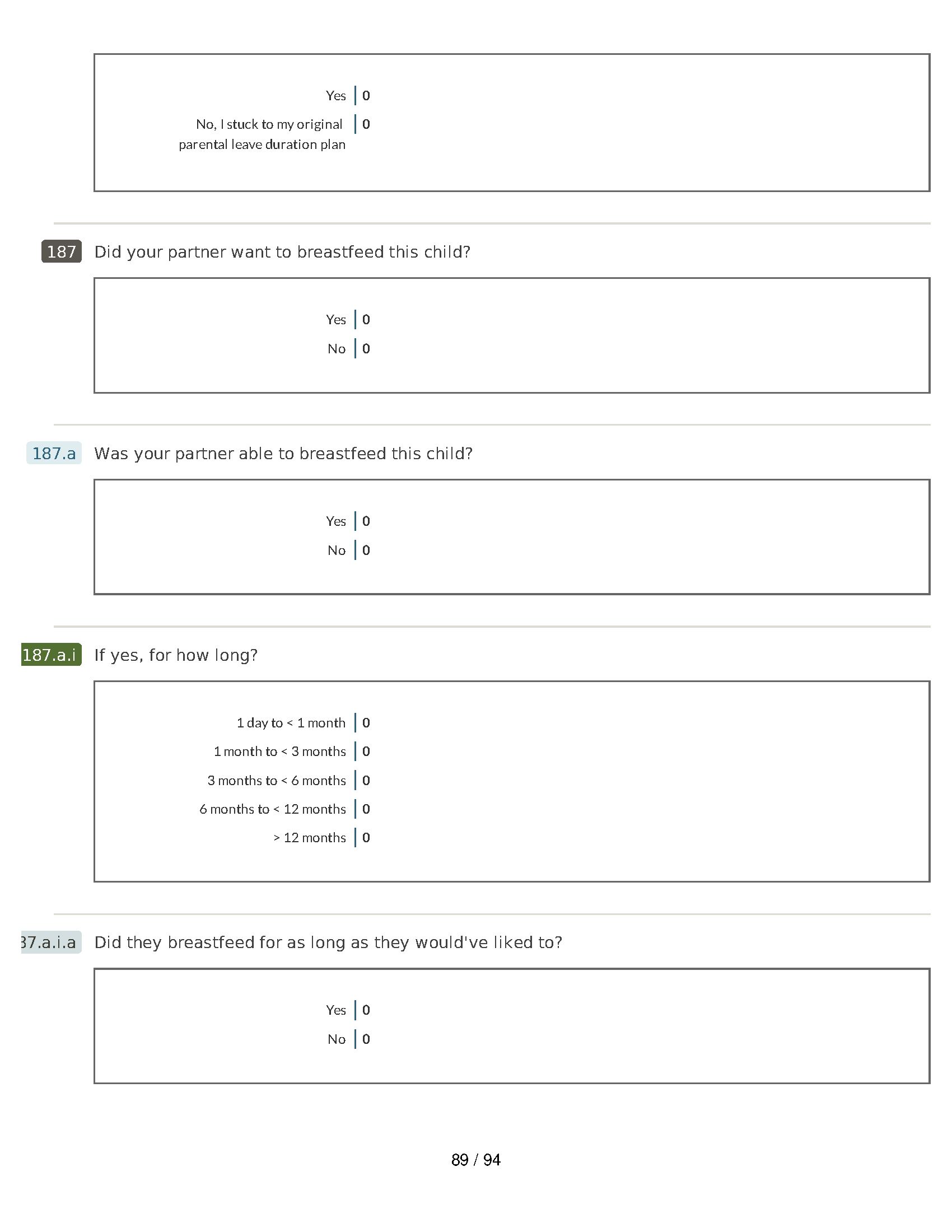

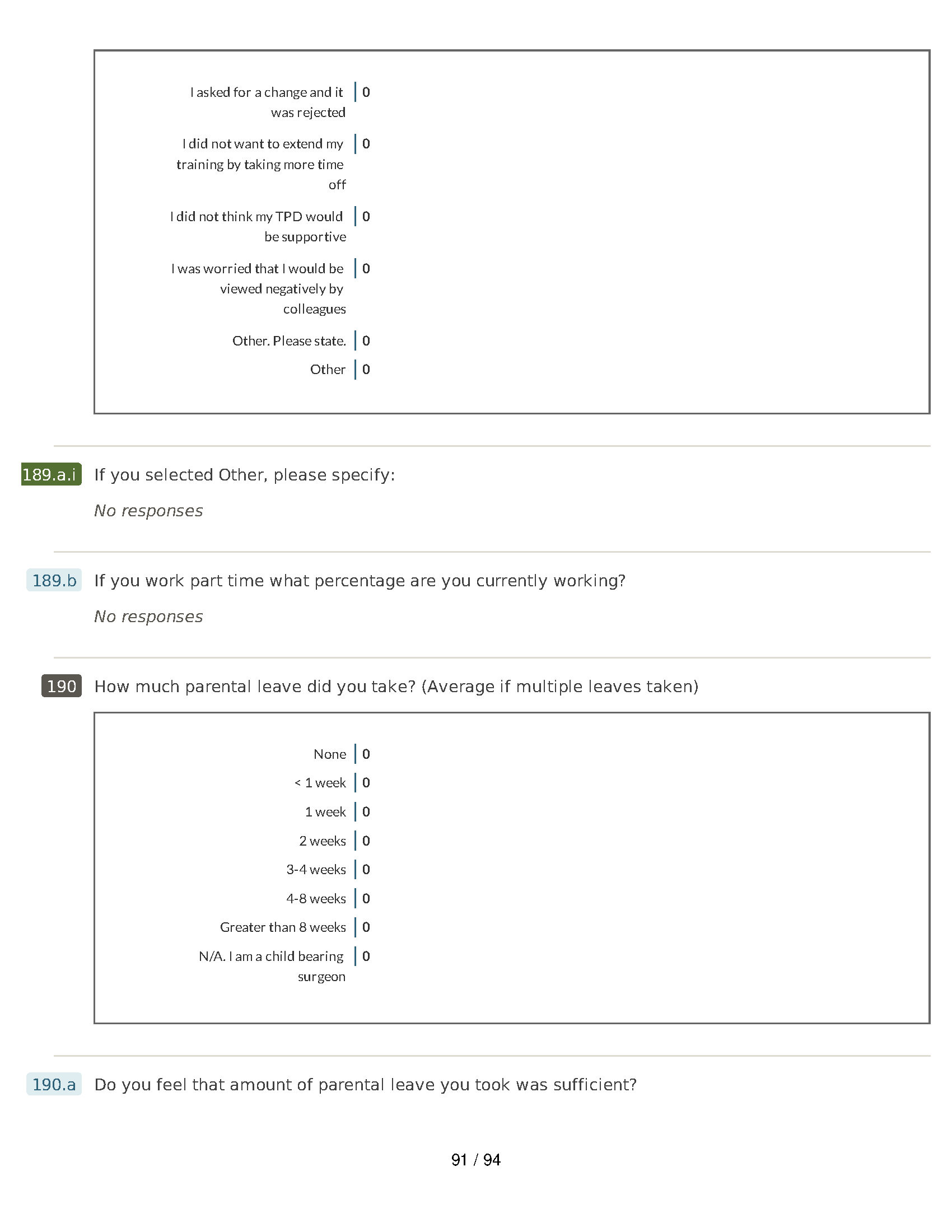

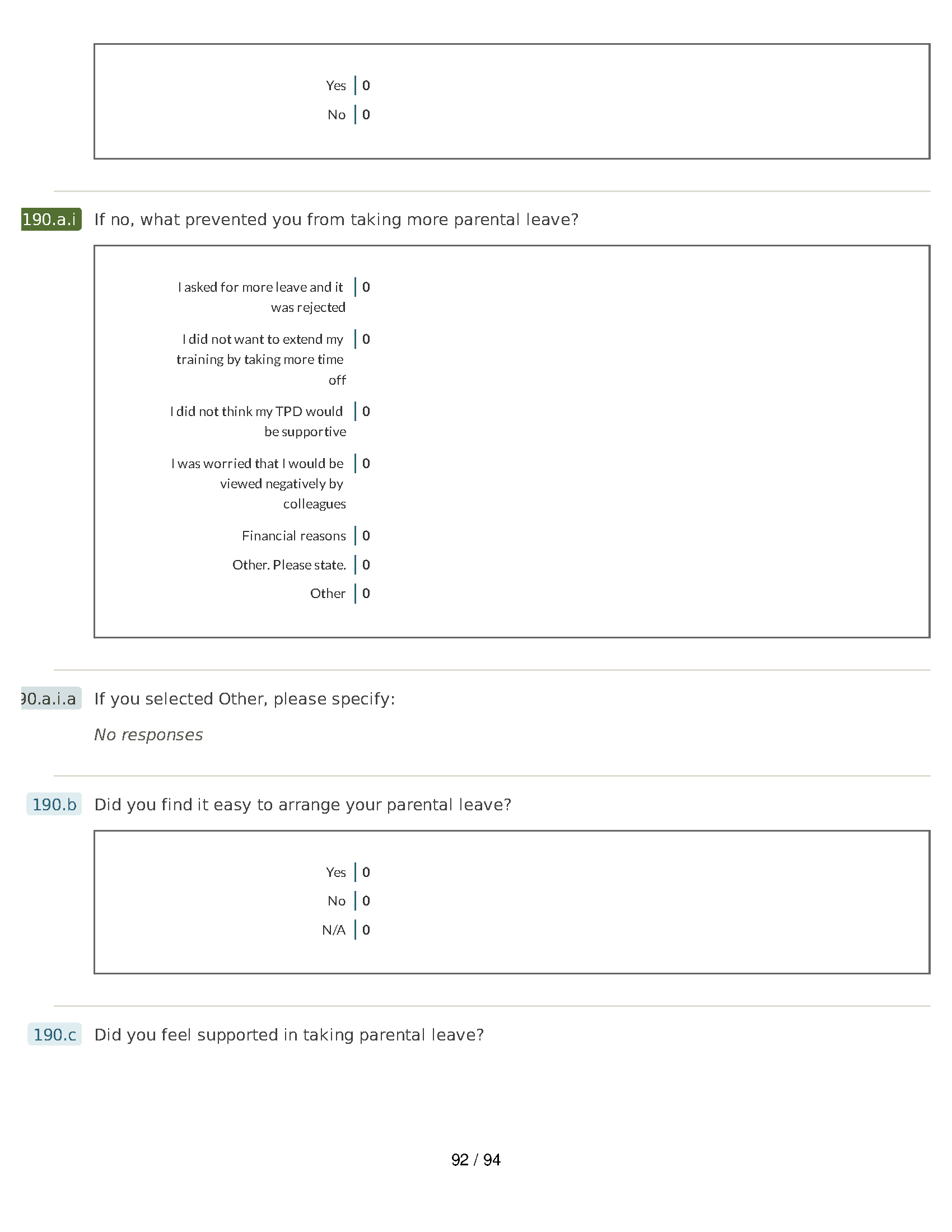

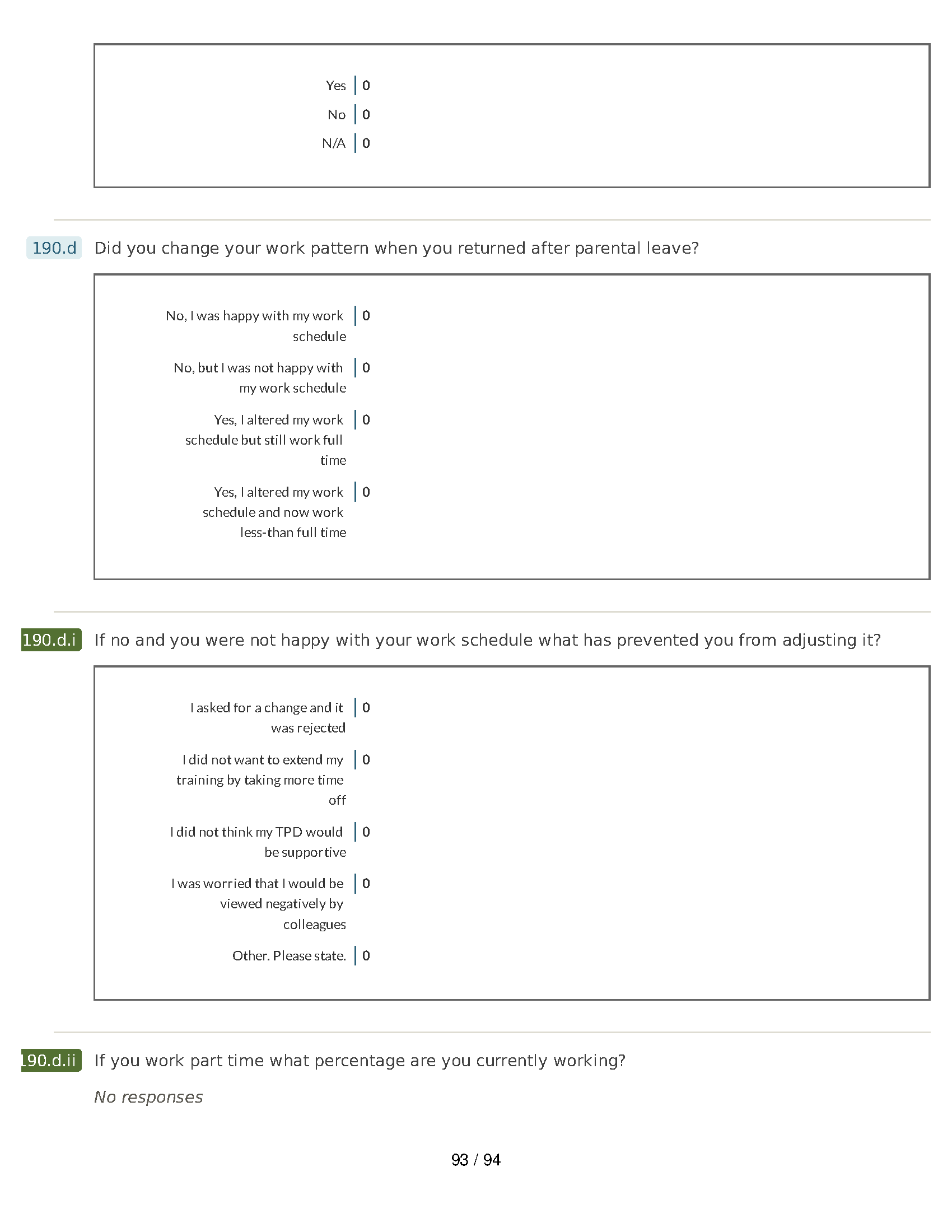

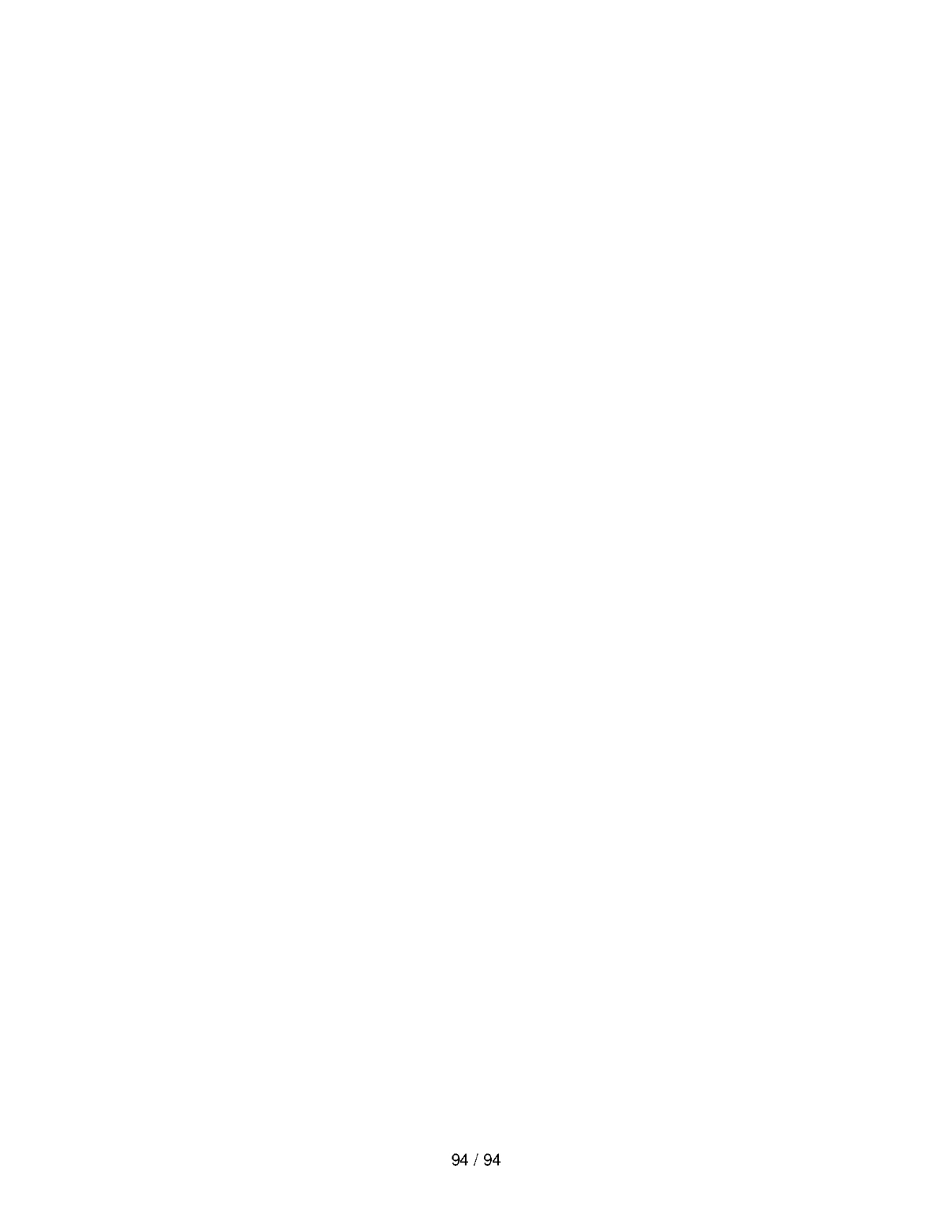
**
